# The human RPS4 paralogue on Yq11.223 encodes a structurally conserved ribosomal protein and is preferentially expressed during spermatogenesis

**DOI:** 10.1186/1471-2199-11-33

**Published:** 2010-05-07

**Authors:** Alexandra M Lopes, Ricardo N Miguel, Carole A Sargent, Peter J Ellis, António Amorim, Nabeel A Affara

**Affiliations:** 1 IPATIMUP, Instituto de Patologia e Imunologia Molecular da Universidade do Porto, R. Dr. Roberto Frias S/N, 4200-465 Porto, Portugal; 2 Department of Pathology, University of Cambridge, Tennis Court Road, Cambridge, CB2 1QP, UK; 3 Department of Biochemistry, University of Cambridge, Tennis Court Road, Cambridge, CB2 1QP, UK; 4 Faculdade de Ciências, Universidade do Porto, 4099-002 Porto, Portugal

## Abstract

**Background:**

The Y chromosome of mammals is particularly prone to accumulate genes related to male fertility. However, the high rate of molecular evolution on this chromosome predicts reduced power to the across-species comparative approach in identifying male-specific genes that are essential for sperm production in humans. We performed a comprehensive analysis of expression of Y-linked transcripts and their X homologues in several human tissues, and in biopsies of infertile patients, in an attempt to identify new testis-specific genes involved in human spermatogenesis.

**Results:**

We present evidence that one of the primate-specific Y-linked ribosomal protein genes, *RPS4Y2*, has restricted expression in testis and prostate, in contrast with its X-linked homologue, which is ubiquitously expressed. Moreover, we have determined by highly specific quantitative real time PCR that *RPS4Y2 *is more highly expressed in testis biopsies containing germ cells. The *in silico *analysis of the promoter region of *RPS4Y2 *revealed several differences relative to *RPS4Y1*, the more widely expressed paralogue from which Y2 has originated through duplication. Finally, through comparative modelling we obtained the three dimensional models of the human S4 proteins, revealing a conserved structure. Interestingly, RPS4Y2 shows different inter-domain contacts and the potential to establish specific interactions.

**Conclusions:**

These results suggest that one of the Y-linked copies of the ribosomal protein S4 is preferentially expressed during spermatogenesis and might be important for germ cell development. Even though *RPS4Y2 *has accumulated several amino acid changes following its duplication from *RPS4Y1*, approximately 35 million years ago, the evolution of the Y-encoded RPS4 proteins is structurally constrained. However, the exclusive expression pattern of *RPS4Y2 *and the novelties acquired at the C-terminus of the protein may indicate some degree of functional specialisation of this protein in spermatogenesis.

## Background

The sex chromosomes of mammals shared a common evolutionary history before acquiring different roles in sex determination but have been shaped differently by divergent selective pressures in the last 180 MYR. The human Y chromosome bears many genes essential for male fertility. In fact, testicular differentiation, the primary event in sex determination, is triggered by the Y-linked gene *SRY*. Moreover, the association of spermatogenic impairment with the presence of microscopic deletions on the long arm of the Y chromosome [1], contributed to the definition of three different regions on Yq that are crucial for spermatogenesis - the Azoospermia Factor regions (AZFa, AZFb and AZFc). The AZFb and AZFc regions are arranged in repetitive sequence blocks with palindromic structure and are recurrently deleted in infertile patients due to intrachromosomal recombination [2,3]. The AZFa region does not have the same palindromic structure, but deletions are provoked in a similar way to the AZFb and c regions by flanking direct repeats leading to misalignment and unequal recombination between sister chromatids [4].

Several candidate genes have been found within the AZF regions, which are expressed at different stages of germ cell differentiation, but their precise function in spermatogenesis has not been fully characterised yet [5]. The lack of functional information on these genes is mainly due to two reasons: i) typically the deletions remove simultaneously more than one gene, hindering the establishment of genotype-phenotype correlations; ii) an appropriate animal model to study the function of human Y-linked genes in spermatogenesis is lacking. In fact, due to the accelerated rate of evolution of the Y chromosome [6-8] there are great differences in gene content across mammalian Y chromosomes, even between closely related species. A striking example is the fact that four genes on the chimpanzee Y chromosome, with active orthologues on the human Y, bear disrupting mutations [9-11]. One is *USP9Y*, a gene in the human AZFa region that is thought to have an important role in spermatogenesis, reviewed in [5]. Mammalian spermatogenesis has been intensively studied in *Mus musculus *but, although some of the genes within the human AZF regions have orthologues on mouse Yp, the single gene that has been proven to be crucial for spermatogenesis in mice, *Eif2s3y *[12], does not have a Y-linked human counterpart. Conversely, the human Y chromosome is also characterised by the presence of genes acquired recently in primate evolution, which are absent from the mouse. Therefore, to gain a better understanding of the spermatogenic process and to identify the genes that are fundamental for male fertility in humans it is of paramount importance to characterise all human Y-linked genes.

The finished sequence of the human Y chromosome [3] has provided a complete list of male-specific genes. Many are multi-copy transcripts expressed in testis, while others are single copy and have homologues on the X chromosome. Classically, the latter have been thought to be ubiquitous proteins that perform basic maintenance functions in all cell types. However, the finding of different regulation during spermatogenesis for the Y-linked gene (*DDX3Y*, formerly *DBY*) and its X homologue has proved that this generalization is inadequate and has prompted us to perform an in depth characterisation of both X- and Y-linked genes of X-Y homologous pairs.

Spermatogenesis is the complex process through which spermatozoa are formed and consists of both mitotic and meiotic divisions, and of morphological cell differentiation involving dramatic changes in the transcriptome and proteome of the germ cells. It is therefore not surprising that the study of mouse germline gene expression has revealed a unique repertoire of transcripts derived from genes, or variants of genes, that are only expressed during male gametogenesis [13].

Following a careful literature and database search we selected for further analysis those single-copy Y chromosome genes that were still poorly characterised and presented novel alternative transcripts, as well as their homologues on the X chromosome. We determined the expression patterns of 20 different transcripts in a panel of human tissues and in biopsies of infertile patients, in an attempt to identify new testis-specific isoforms involved in germ cell development in humans. Most isoforms analysed were detected in testis and one gene, belonging to the human ribosomal S4 family (*RPS4Y2*), showed restricted expression in testis and prostate, in contrast with its X-linked homologue, which was ubiquitously expressed.

Eukaryotic ribosomal protein S4 (S4e) is X-linked in mammals [14] and a Y-linked homologue (*RPS4Y1*) is present in all primate lineages analysed to date [15,16]. In humans, a second copy of the Y-linked gene (*RPS4Y2*) was described [3] which originated by duplication before the radiation of Old World Monkeys, approximately 35 million years ago [15]. The report where *RPS4Y2 *was first described includes conflicting information regarding its expression pattern [3]. Andrés *et al*. [15] performed an evolutionary analysis of the *RPS4XY *gene family in primates and claimed testis-specific expression for *RPS4Y2 *in humans but they have not presented any supporting evidence. We have now characterised the expression patterns of all the coding isoforms encoded by this gene family and found different breadth of expression for the transcripts encoded by each gene, but also for alternative transcripts arising from the same gene, and we have confirmed restricted expression for *RPS4Y2 *in testis and prostate. Moreover, we have determined by highly specific quantitative real time PCR that *RPS4Y2 *is more highly expressed in testis biopsies containing germ cells. Finally, we performed comparative modelling of the three human RPS4 proteins, in order to evaluate the structural context of the differences observed between these proteins and to try to predict their functional impact.

## Results

### Expression patterns of X/Y isoforms in human tissues

For each X/Y gene pair a careful search for protein-coding alternative transcripts was performed in several databases: Ensembl http://www.ensembl.org, NCBI http://www.ncbi.nlm.nih.gov/ and at the UCSC Genome Browser http://genome.ucsc.edu/. We specially focused on those isoforms presenting different UTRs (at the 5' and 3' ends), since these are most likely to incorporate different regulatory regions, and did not aim to characterise any isoforms that would give rise to truncated proteins and/or lacking relevant functional domains. In some cases it was not possible to distinguish very similar isoforms transcribed by the same locus or highly homologous genomic sequences, such as *EIF2S3X *and its autosomal copy.

We analysed the expression patterns of 20 different transcripts in a panel of 15 somatic tissues plus testis and ovary, by standard end point RT-PCR, using isoform-specific primers (Additional file 1). All Y chromosome isoforms and most X-linked ones were expressed in testis but the majority were also present in other tissues (Additional file 2). In some cases, unexpected bands were present in the gel, indicating the presence of additional transcripts. None of these extra bands suggested restricted expression in testis and therefore were not relevant to the present study. In the ovary, none of the Y-linked isoforms were detected, confirming the specificity of the primers used.

**Figure 1:**
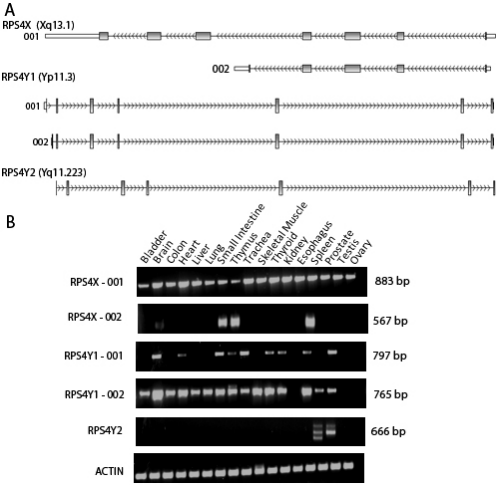
**Analysis of expression of *RPS4 *transcripts**. **(A) **Structure of the transcripts analysed; the arrows represent the direction of transcription (forward or reverse strand). **(B) **Expression patterns of the *RPS4X, RPS4Y1 *and *RPS4Y2 *transcripts in a panel of 17 human tissues (Ambion First Choice RNA Panel). Each tissue sample is derived from a pool of individuals (males and/or females). Last lane is the no template control.

Interestingly, we found different expression patterns for the genes encoding each of the proteins of the ribosomal S4 family (*RPS4X/RPS4Y1/RPS4Y2*). While the canonical *RPS4X *transcript, corresponding to the full length ORF (*RPS4X-*001, ENST00000316084) was expressed in almost all tissues surveyed, the Y-linked isoforms were somewhat more restricted, *RPS4Y2 *being only found in testis and prostate (Figure 1). An alternative, protein-coding transcript arising from *RPS4X *is annotated in Ensembl (*RPS4X*-002, ENST00000373626), which results in a shorter protein. This transcript was only detected in small intestine, thymus and spleen and at vestigial levels in brain. The analysis of this gene family revealed that only one of the Y-linked transcripts is more widely expressed, *RPS4Y1-*002 (ENST00000430575), and therefore the remaining transcripts are good candidates for a male-specific function. The two *RPS4Y1 *transcripts incorporate alternative first exons, *RPS4Y1-*002 giving rise to a protein with an N-terminus nine amino acids longer, as compared to the canonical protein encoded by *RPS4Y1-*001 (NP_000999.1). The sequence available in Ensembl for *RPS4Y1*-002 lacks a termination codon and a 3'UTR; nevertheless, we have detected by RACE-PCR, using a forward primer specific for *RPS4Y1*-002, a full length transcript incorporating the whole ORF and 3'UTR of *RPS4Y1 *as well as a poly A tail (the cDNA sequence has been submitted to dbEST - Acession GT091164).

In addition to the canonical *RPS4Y2 *transcript (ENST00000288666), in testis and prostate we detected a longer transcript arising from this locus that incorporates a previously uncharacterised exon, located between exons 2 and 3. However, the *in silico *translation of the corresponding mRNA resulted in a truncated protein, due to the presence of an early stop codon. We performed a RACE-PCR specific for that transcript but were unable to get any evidence for the existence of an alternative upstream sequence that could give rise to a non-truncated ORF (results not shown). Therefore, it is not clear if this alternative transcript has a functional role, although a putative regulatory function cannot be ruled out. In prostate, a third *RPS4Y2 *transcript was detected but, since this isoform was only expressed in this tissue, we did not further characterise it.

To determine the relative abundance of the different transcripts in testis we performed quantitative real time PCR in commercially available testis RNA derived from a pool of three males (Ambion) and compared the detection threshold of each transcript to that of *Beta Actin*. *RPS4X*-001 is the most highly expressed of all the three ribosomal proteins S4 analysed. *RPS4Y1*-002 (ENST00000430575) is the second most abundant *RPS4 *transcript while *RPS4Y2 *is the least abundant (Table 1).

**Table 1:** Relative expression of *RPS4X/Y *isoforms in human testis.

Isoform	Identifiers	Delta Ct*
*RPS4X*-001	ENST00000316084; NM_001007.4	7,3
*RPS4Y1-*002	ENST00000430575	11,9
*RPS4Y1*-001	ENST00000250784; NM_001008.3	15,3
*RPS4Y2*-longer isoform		19,0
*RPS4Y2*	ENST00000288666; NM_001039567.2	21,2

* Difference in the detection threshold of each target to *Beta Actin *is presented (average of two experimental replicates) in a pool of total testis RNA derived from three males (Ambion).

### Expression of ribosomal S4 genes in testis biopsies

**Figure 2:**
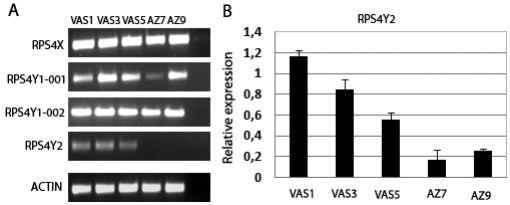
**Expression of *RPS4 *genes in testis biopsies of five infertile individuals**. All samples had been previously characterized by histology and global expression profiling [17] (3 subjected to vasectomy - VAS1, VAS3, VAS5; 2 azoospermic patients - AZ7, AZ9). **(A) **End-point detection of RT-PCR products of *RPS4XY *isoforms by electrophoresis. Last lane is the no template control. **(B) **Quantitative real time PCR using an *RPS4Y2 *TaqMan probe. The values correspond to the mean of two independent measurements of relative expression using *GAPDH *as an endogenous control for normalization. The error bars refer to one standard deviation to the mean of the two experimental replicates.

The testis is a complex organ, where several spermatogenic and somatic cell types co-exist. To determine the cell type from which the transcripts detected in testis are arising we compared the expression levels of each *RPS4 *transcript in a set of testicular biopsies of infertile patients that do not present known Y chromosome deletions (three individuals subjected to vasectomy but with conserved spermatogenesis and two azoospermic patients with no evidence for the presence of germ cells). This biopsy material had been previously characterised by histology and global expression profiling [17]. By conventional end-point RT-PCR all isoforms were detected in both groups of samples (with and without germ cells), except *RPS4Y2 *that was only present in the biopsies containing spermatogenic cells (Figure 2A). These results suggested that *RPS4Y2 *is expressed in the germ cells.

Due to the weak amplification signal obtained from biopsy samples we have performed a method known to be more sensitive, quantitative real time RT-PCR, with a *RPS4Y2 *TaqMan probe designed to discriminate *RPS4Y2 *from the two other paralogues. Using this approach we detected *RPS4Y2 *cDNA in all of the samples analysed, but the expression levels of the gene were approximately four times higher in the samples of biopsies from individuals with conserved spermatogenesis, when compared to those from azoospermic patients (Figure 2B). The high heterogeneity observed within the group of samples from patients subjected to vasectomy may reflect variation in the cell type content in the seminiferous tubules of these patients. In all cases ΔCt (Ct _*RPS*4*Y*2 _- Ct _*GAPDH*_) was equal to or greater than 9, confirming the low expression levels of the gene.

### Analysis of *RPS4Y2 *promoter region

*RPS4Y2 *has originated from *RPS4Y1 *through duplication and a BLAST alignment of the genomic sequence upstream the transcription start site of the two genes indicates that the 692 bp of the 5'-flanking sequence of Y2, on Yq11.22, were derived from the Y1 sequence, on Yp11.3 (results not shown). However, there are several sequence differences between the two genes in this region that may underlie their different expression patterns. Yoshihama *et al*. [18] have performed an extensive analysis of the promoter regions of human ribosomal genes and found that transcription always starts at a C residue within a characteristic oligopyrimidine tract. This sequence element is disrupted by a C-to-G mutation in *RPS4Y2*, which may contribute to the more restricted expression pattern of this gene. Additionally, the human *RPS4Y2 *mRNA AF497481.2 includes a 5'UTR that extends beyond that position, indicating that *RPS4Y2 *transcription starts further upstream the ATG, in a different sequence context.

In the analysis of expression presented above we have detected both *RPS4Y1 *transcripts predicted by Ensembl, by anchoring the primers in the alternative UTRs. Our results indicate that in some instances *RPS4Y1 *transcription may also start upstream the described oligopyrimidine tract. The UTR of *RPS4Y1*-001 is similar to the sequence upstream the start codon of *RPS4Y2*; *RPS4Y1*-002 transcription starts 319 bp downstream of the first ATG, within the first intron of the gene. Interestingly, there is a short oligopyrimidine tract (7 bp) immediately upstream this alternative ATG. In the corresponding position of *RPS4Y2 *intron, the ATG is not present, due to a mutation.

**Figure 3:**
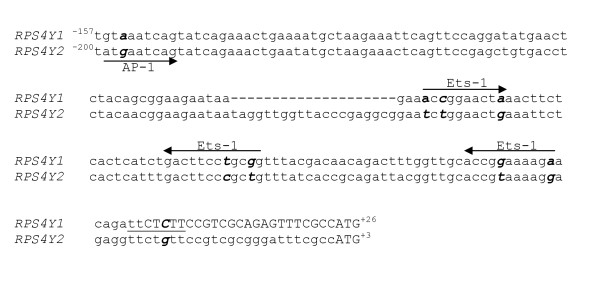
**Sequence motifs found within the promoter regions of *RPS4Y1 *and *RPS4Y2***. The numbers indicate nucleotide positions relative to the TSS, according to [18]. For RPS4Y2, we considered that the TSS overlaps the ATG. Hyphens represent inserted/deleted nucleotides. The olygopyrimidine tract overlapping the *RPS4Y1 *transcription start site is underlined. Arrows indicate the orientation of the predicted transcription factor binding sites (forward or reverse strand). The positions within the sequence motifs that differ between the two genes are in italic.

Using MatInspector 8.0 we have searched for binding sites for transcription factors in the 500 bp of genomic sequence upstream the start codon of *RPS4Y2 *and the corresponding sequence of *RPS4Y1 *(upstream the first ATG). In both genes several putative binding sites were predicted with high scores (matrix similarity =95.0), in forward and in reverse orientation (Figure 3). We also run TFSEARCH http://www.cbrc.jp/research/db/TFSEARCH.html in the same sequences and report here those sites predicted by both programs. Within the *RPS4Y1 *sequence, three potential sites for different ETS factors were detected, one of which had been previously reported [18]. Additionally, two potential binding sites for the GATA-binding protein family and one for each of the following transcription factors were found: C/EBPb, Ik-2 and AML-1a. *RPS4Y2 *presents DNA sequence motifs potentially recognised by the following factors: AP-1, Sox5, MZF-1, v-Myb and AML-1a.

### 3D structure prediction of RPS4X, RPS4Y1 and RPS4Y2

#### Sequence analysis

There is high amino acid identity between the three human proteins, denoting their common origin (RPS4X, RPS4Y1 and RPS4Y2 accession numbers NP_000998.1, NP_000999.1 and NP_001034656.1, respectively). As expected, the two Y-coded proteins are more similar (~94% identity) since *RPS4Y2 *arose more recently through gene duplication from *RPS4Y1 *[15].

Additional file 3 shows an alignment of the amino acid sequence of the three human RPS4 proteins. Only one position shows different amino acids in the three sequences, position 171, that is an Asp in RPS4X, an Asn in RPS4Y1 and a Ser in RPS4Y2. This substitution changes a negatively charged amino acid in RPS4X to a polar amino acid, with different chemical properties, in the two RPS4Y proteins. Other non-conservative differences, between RPS4X and one or both of the Y-linked sequences, are listed in Table 2. A BLAST alignment of the eukaryotic RPS4 family (Additional file 4) shows several conserved positions, notably: His36 is absolutely conserved; the residue at position 27 is always an aromatic amino acid; the residues at positions 60 and 75 are always negatively and positively charged amino acids, respectively. The amino acid positions that are more highly conserved across the RPS4 family are clustered in the N-terminal domain, suggesting that this domain is involved in an important function, probably interacting with the ribosome.

**Table 2:** Non-conservative substitutions observed in the RPS4Y proteins.

Residue	Type of substitution
78	Thr → Val: polar in X, hydrophobic in Y1 and Y2
104	Asp → Asn: negative in X and Y1, polar in Y2
108	Arg → Cys: positive in X and Y1, polar/hydrophobic in Y2
130	Phe → Thr: hydrophobic in X, polar in Y1 and Y2
133	Thr → Val: polar in X and Y2, hydrophobic in Y1
165	Glu → Gly: negative in X, glycines in Y1 and Y2
170	Thr → Ile: polar in X and Y2, hydrophobic in Y1
171	Asp → Asn/Ser: negative in X, polar in Y1 and Y2
184	Thr → Ile: polar in X, hydrophobic in Y1 and Y2
230	Lys → Asn: positive in X, polar in Y1 and Y2
258	Ala → Thr: hydrophobic in X and Y2, polar in Y1

### Comparative modelling of RPS4X, RPS4Y1 and RPS4Y2

The fold recognition program FUGUE [19] identified the ribosomal protein S4E (pdb-Id: 3KBG) as an appropriate template for nearly the full length of the RPS4 proteins; the ribosomal protein S4 delta 41 (pdb-Id: 1C06) was identified as a suitable template for the N-terminal domain of the RPS4 proteins and the 50S ribosomal protein L24P (pdb-Id: 1JJ2 chain S) for the C-terminal domain. Comparative models of the full length of RPS4X, RPS4Y1 and RPS4Y2 were obtained based on the three templates. The protein sequence used to build the RPS4Y1 model corresponds to isoform RPS4Y1-001.

**Figure 4:**
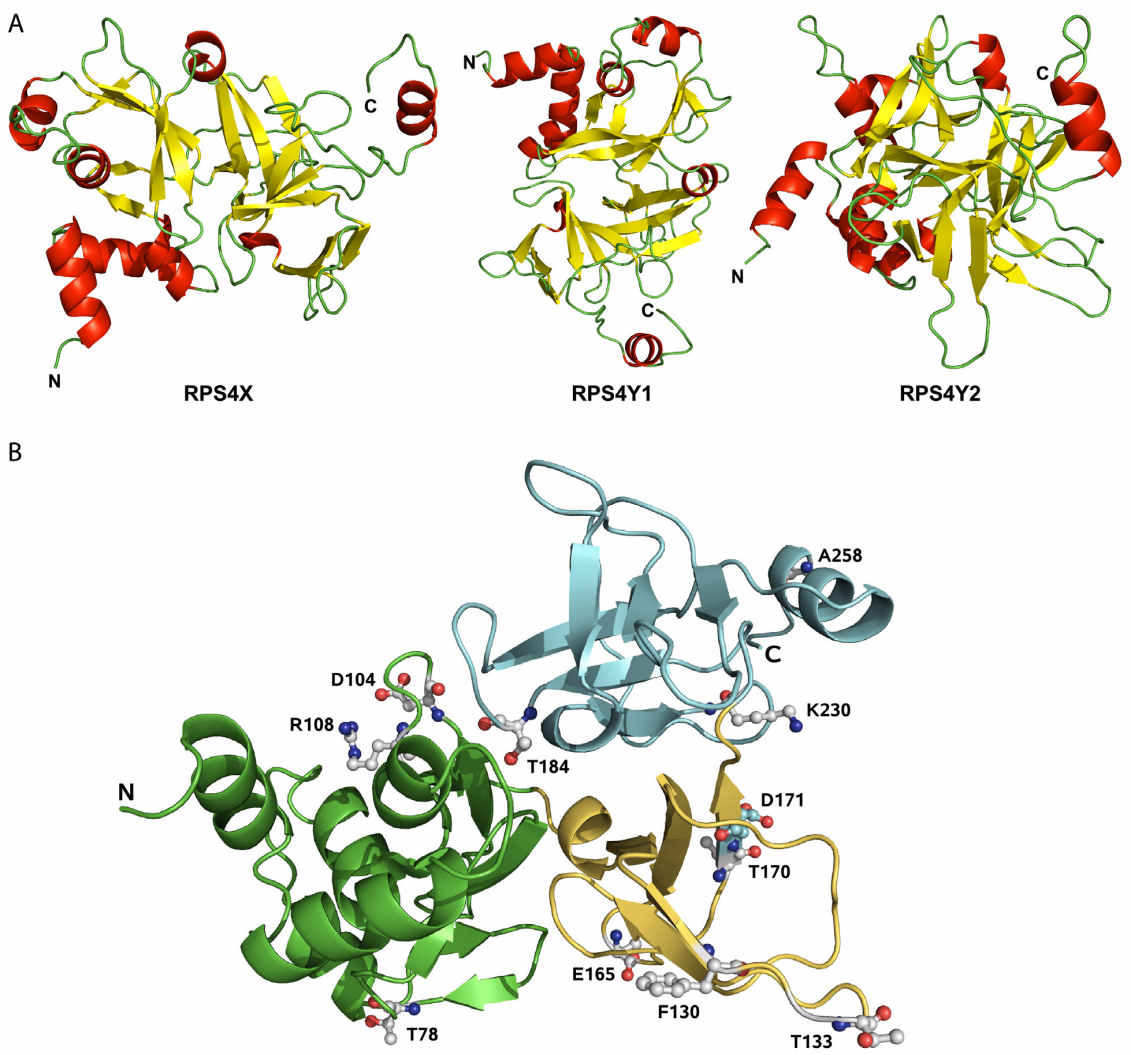
**Three dimensional models of isolated RPS4 proteins**. The N and C termini are marked. (**A**) Structure of each RPS4 protein. β strands are in yellow, α helices in red and loops in green. (**B**) RPS4X showing the one residue that is different in the three RPS4 proteins (carbon atoms in cyan, oxygen atoms in red and nitrogen atoms in blue) and the residues that are different, with non-conservative substitutions, in one or both of the RPS4Y proteins (carbon atoms in grey, oxygen atoms in red and nitrogen atoms in blue). N-terminal domain is in green, central domain in yellow and C-terminal domain in cyan.

Figure 4A shows the structure of the models of isolated RPS4X, RPS4Y1 and RPS4Y2 from different points of view. The RPS4 proteins are composed of three structural domains: an N-terminal domain (aa 1-113), a central domain (aa 116-173) and a C-terminal domain (aa 180-263). The structure of the N-terminal domain is composed of a five-stranded β sheet with five α helices and a small two stranded β sheet located at the same side (Figure 4A - left side for RPS4X, top for RPS4Y1 and left for RPS4Y2); one helix and parts of the two β sheets contact the other domains of the protein. The central domain is formed by a small helix, a three stranded β sheet that interacts with the other two domains and a β hairpin (Figure 4A - top centre for RPS4X, back centre for RPS4Y1 and back right for RPS4Y2). The structure of the C-terminal domain includes six β strands arranged as an irregular β barrel and two α helices (Figure 4A on the right side for RPS4X, bottom for RPS4Y1 and bottom right for RPS4Y2); interactions with the other domains are observed through the β barrel, the small helix and some loops.

### Location of residues that differ between the three RPS4 proteins

Several non-conservative amino acid substitutions can be observed in the RPS4 Y-coded proteins, as compared to the highly conserved RPS4X, which are distributed throughout the three structural domains of the proteins (see Table 2). Figure 4B shows the location, on the predicted RPS4X structure, of the only residue that is different in all three proteins (position 171, carbon atoms in cyan), as well as those presenting non-conservative substitutions in one or both of the RPS4Y proteins (carbon atoms in grey).

It can be seen that RPS4X residue Thr184 is located in the interface between two domains (N-terminal and central) and shows strong inter-domain interactions (69Å2 in RPS4X and 64Å2 in RPS4Y2). The presence of a different amino acid at position 184 in the Y chromosome proteins contributes to a modification of the relative position of the two domains, compared to RPS4X.

At position 171, an interesting difference can be observed between RPS4Y2 and the other two RPS4 proteins. A hydrogen bond is formed between S4Y2 Ser171, in the central domain, and Asn232, in the C-terminal domain, which is not present in either S4X or S4Y1. Therefore, the presence of different amino acids at position 171 can affect the relative position of these domains.

No intra-molecular interactions are seen for the other residues shown in Figure 4B. The residues at positions 104, 108, 165 and 170 are all charged or polar in RPS4X. Interestingly RPS4X residues Glu165 and Thr170 are replaced by hydrophobic amino acids in the Y-coded proteins (see Table 2). If exposed to the solvent in the assembled ribosome, the new hydrophobic residues are good candidates for interactions with other proteins. Similarly, RPS4X residues Asp104 and Arg108, which are charged aminoacids, are replaced by polar residues in RPS4Y2.

## Discussion

We sought to characterize the expression patterns of several X-Y gene pairs in a panel of human somatic tissues and testis in order to evaluate the contribution of sex-linked isoforms to the transcriptome of the male germline. Considering testis-specific expression as a strong indication of a function in spermatogenesis, we then focused on a particularly interesting gene family, Ribosomal Protein S4, presenting five protein coding isoforms with diverse expression patterns, *RPS4Y2 *being only expressed in testis and prostate. Interestingly, the quantification of transcripts in testis biopsies strongly suggests that *RPS4Y2 *is more highly expressed during spermatogenesis, although this trend should be confirmed in a larger cohort of patients.

Ribosomes are the site of mRNA translation and protein synthesis and comprise an assembly of a few rRNA molecules and a large number of proteins. Although the major biochemical steps in protein synthesis are carried out by the rRNA, ribosomal proteins (RPs) are crucial for efficient translation and for ribosome assembly. However, the precise role of each protein within the ribosome is not clearly defined, since there is a great synergy between rRNA and RPs in the different steps of translation.

The global structure of the prokaryotic and eukaryotic ribosome is similar, consisting of two distinct subunits, small and large: 30S and 50S in the prokaryotic ribosome; 40S and 60S in the eukaryotic counterpart. The corresponding subunits in these two cell lineages differ in the size of the rRNA molecules and in the number and type of ribosomal proteins they incorporate. In fact, although there is high conservation of ribosomal proteins, both at the sequence and structural level, within each domain of life (eubacteria, archaea and eukarya), some RPs are restricted to one evolutionary lineage [20]. For example, the eukaryotic homologue of S4 is S9, while S4e is only found in archaeal and eukaryotic ribosomes [21].

We have built comparative models of the structures of RPS4X, RPS4Y1 and RPS4Y2 in order to evaluate the impact of the differences observed at the sequence level. Overall the three proteins show very similar structures, which is suggestive that all may be functional. There are however a few noteworthy differences. Due to the presence of a different amino acid at position 184, the Y chromosome proteins show differences in the relative position of the central and N-terminal domains, compared to RPS4X. This, in turn, may produce slightly different interactions with the ribosome, or with neighboring proteins within the small subunit.

Additionally, in RPS4Y2 the presence of a hydrogen bond between Ser171 and Asn 232 creates a new interaction between the central and the C-terminal domains, which determines their relative position. Due to its high conservation, the N-terminal domain of eukaryotic S4 proteins is most likely involved in the interaction with the ribosome, in which case the C-terminal domain will be more exposed, at the ribosome surface. The differences at the C-terminus of RPS4Y2 may therefore reflect the interaction with different ribosomal proteins or extra-ribosomal factors, as compared to RPS4X and RPS4Y1.

High resolution crystal structures of the small and large subunits of the eubacterial ribosome and the low-resolution structure of the archaeal large subunit have been determined, as well as that of several of the proteins bound to the ribosome [22-24]. Recently, the atomic model of yeast 80S ribosome has been built, through cryo-EM and RNA and protein homology modelling [25]. Unfortunately, eukaryotic S4 was not included in this model and therefore we could not dock our models of the human RPS4 proteins to a 60S ribosomal subunit. It will be interesting in the future to test whether the three proteins present similar interactions with RNA and neighbouring proteins or interacting factors, in the small subunit.

The ribosomal small subunit controls mRNA binding and ensures translation accuracy in the decoding process [26,27]. Mammalian ribosomal protein S4 is located at the interface between the small and the large subunits of mammalian ribosomes and is in contact with the initiation factor eIF-3 [28]. Therefore RPS4 is most likely involved in the initiation of translation.

Human RPS4Y is able to rescue a temperature-sensitive hamster S4 mutant cell line [29]. Zinn *et al*. [30] confirmed the presence of both human S4 proteins in male placental ribosomes using isoform-specific antisera and found that the S4Y protein was only 15% as abundant as S4X. We quantified the expression levels of each RPS4 isoform in testis and found that *RPS4Y *transcripts are also less abundant than their X-linked counterparts in this tissue. *RPS4Y2 *is the least abundant of all the transcripts tested. According to these results only a minor fraction of the ribosomes incorporate the Y-coded isoform of RPS4 and, in testis, an even smaller subset may include RPS4Y2. It would be important to test the presence of RPS4Y2 in human ribosomes; however, due to the high homology between the two proteins encoded by the Y chromosome (approximately 94%) it is virtually impossible to design an antibody which specifically detects RPS4Y2.

From our analysis, one of the *RPS4Y1 *transcripts (*RPS4Y1-*002; ENST00000430575) is apparently a more faithful homologue of *RPS4X*, since it presents a wider breadth of expression than *RPS4Y1*-001. In spite of initial association of RPS4 haploinsufficiency and Turner Syndrome [14,29], the finding that X chromosome monosomy leads to a similar phenotype even in animal species that do not have an Rps4 homologue on the Y chromosome, where *Rps4X *might therefore be subject to inactivation, has raised some uncertainties about its role in this respect [31]. In fact, in humans the global dosage of RPS4 does not seem to be crucial, since in males the ubiquitous RPS4Y protein is produced at much lower levels than its X homologue, while in females *RPS4X *escapes inactivation and therefore is present in double dosage.

In the testis, even in the presence of a second Y-linked copy, the scenario is not expected to change drastically in terms of overall gene dosage, due to the extremely low expression levels of *RPS4Y2*. Histologically testis is a very heterogeneous organ and in the seminiferous tubules cells of the germline, in different states of differentiation, coexist with somatic cells (Sertoli). The fact that *RPS4Y2 *mRNA levels are higher in the testis biopsies where germ cells are present suggests that this gene is either transcribed in both cell types (somatic and spermatogenic) or it may only be expressed in somatic cells, but at a higher rate during spermatogenesis. Its location within the azoospermia region AZFb on the Y chromosome makes it a good candidate for male infertility.

Most ribosomal proteins are constitutively expressed in several tissues. To understand the restricted expression of *RPS4Y2 *we have inspected the 5'-flanking region of the gene for any features that could distinguish this sequence from the promoter region of *RPS4Y1*, its most closely related paralogue. Interestingly, the polypirimidine tract that characterises the promoters of the ubiquitously expressed ribosomal proteins, including that of *RPS4Y1*, is disrupted in *RPS4Y2*. This difference may explain the loss of constitutive expression. To determine the additional features of the Y2 promoter that led to the acquisition of prostate and testis-specific expression, it will be necessary to perform functional experiments. In this context, the presence of a potential binding site for the transcription factor AP-1 within the proximal 5'-flanking sequence of RPS4Y2 is noteworthy. Transcription factors of the activator protein 1 (AP-1) class are expressed in the rodent testis in a developmental-stage dependent manner and different lines of evidence suggest multiples roles of these proteins during normal spermatogenesis [32,33]. It is also possible that other yet uncharacterized enhancer/insulator elements in the genomic sequence in Yq11.22, farther upstream the RPS4Y2 promoter, are capable of regulating the expression of this gene in testis and prostate.

Recently, mutations in several ribosomal proteins have been linked to genetic diseases, many of which interfere with ribosome biogenesis (for a recent review see [34]). It is also known that defects in ribosome synthesis elicit cell cycle arrest and apoptosis [35,36]. Therefore, it is plausible that deficits in specific ribosomal proteins may have some bearing on infertility or subfertility phenotypes, to which the particular contribution of *RPS4Y2 *should be further explored.

## Conclusions

Although most ribosomal proteins are ubiquitous, one of the members of the RPS4 family has restricted expression in testis and prostate. The three dimensional models of the human S4 proteins revealed a conserved structure, suggesting that all are involved in RNA processing in the ribosome. Nevertheless RPS4Y2 shows different inter-domain contacts and the potential to establish specific interactions. Moreover *RPS4Y2 *has acquired an exclusive expression pattern that suggests that it may have a role in germ cell development and its location within the AZFb region makes it a good candidate for male infertility.

## Methods

### Expression patterns of X/Y isoforms in human tissues

A panel of RNA samples from human tissues were obtained from Ambion (First Choice Total RNA Survey Panel). Each of this is a pool of RNA from several donors. For first-strand cDNA synthesis 1 μg of total RNA was used as template. RNAs were first denatured at 70°C for 5 minutes and cooled on ice. Reactions were incubated at 42°C 60 minutes in a final volume of 20 μl with reaction buffer (as supplied by the manufacturer), 1 mM of each dNTP, 0.5 units of RNAseOUT, 2.5 μM of oligodT and 15 units of Promega AMV Reverse Transcriptase (Promega Reverse transcription System, Promega, USA). A final step of 5 minutes at 95°C was included to inactivate the enzyme. PCR amplification was performed with isoform-specific primers, taking advantage of sequence differences between the X and Y chromosomes and anchoring the primers in variable exons (for primer sequences see Aditional file1: Primers used in the expression studies). All primers were designed to span exon-exon boundaries, to prevent amplification of possible genomic DNA contaminants. Typically 50 ng of cDNA were amplified using the HotStar HiFidelity Polymerase Kit (Qiagen) in a 15 μl reaction volume comprising 0.3 μM of each primer. PCR conditions were the following: 15 min pre-incubation step at 94°C, 32-35 cycles of denaturation at 94°C for 30 sec, annealing for 1 min at the respective AT for each primer pair and extension at 72°C for 1 min, followed by a final extension step at 72°C for 10 min. The PCR products were visualised after electrophoresis in 1.5-2% agarose gels, to allow the discrimination between molecules of similar molecular weight. Whenever possible, each of the PCR products was directly sequenced; if more than one band were detected in the gel each was excised, cleaned up using QIAquick Gel Extraction Kit (Qiagen) and the DNA sequenced sequenced with BD Terminator chemistry (Applied Biosystems).

#### RACE-PCR in testis RNA

5'RACE and 3'RACE-ready cDNAs were prepared from 1 μg of total testis RNA (Ambion) using the BD SMART RACE cDNA amplification kit (Clontech). PCR fragments were amplified with universal primers included in the kit and gene-specific primers using the RACE-ready cDNAs as template, according to the manufacturer' protocols. PCR fragments were gel-purified (Qiaquick, Qiagen) and sequenced.

#### One-step real time RT-PCR using SybrGreen

Isoform-specific primers, designed to span exon-exon boundaries and to generate amplicons of less than 200 bp, were used for relative quantification of the different transcripts in total testis RNA. One-step quantitative real-time PCR reactions were carried out using 100 ng of RNA in a reaction volume of 20 μl comprising QuantiTect SYBR Green RT-PCR Kit (Qiagen) according to the manufacturer's protocol; the resulting fluorescence was quantified using an iCycler system (Bio-Rad). Each reaction was performed in triplicate and melt curve data were obtained to confirm amplification of the correct product in each well. *Beta Actin *was used as an endogenous control for the amount of RNA in each sample and ΔCt was calculated as the difference between target Ct and Beta Actin Ct. For primer sequences see Additional file 1.

### Expression of ribosomal S4 genes in testis biopsies

#### Subjects and samples

The clinical samples used in this study have been described and characterised in [17]. RNA preparation and cDNA amplication where also performed according to [17].

#### Two-step real time RT-PCR using a custom RPS4Y2 TaqMan assay

The TaqMan probe used (custom assay RPS4Y2-260) was designed to span the junction between exon 3 and exon 4, where lies a single base difference that distinguishes *RPS4Y2 *from *RPS4X *and *RPS4Y1*. Quantitative real-time RT-PCR reactions were carried out using approximately 2 ng of SMART cDNAs in a reaction volume of 25 μl comprising TaqMan Gene Expression Master Mix (Applied Biosystems), according to the manufacturer's protocol; the resulting fluorescence was quantified using an ABI7000 system (Applied Biosystems). *GAPDH *expression assay (Applied Biosystems) was performed as an endogenous control. Each reaction was performed in triplicate and two experimental replicates where performed. Efficiency of primers and quantity of cDNA in each well were derived from an experimentally determined standard curve; only reactions with r^2 ^= 0.99 and with a standard curve slope typically -3.1 = S = -3.6 were accepted.

### Comparative modelling of RPS4X, RPS4Y1 and RPS4Y2

Fold recognition was carried out by the program FUGUE [19]. FUGUE identifies homologues by comparison of sequence profiles against structural profiles of homologous protein families taken from the HOMSTRAD database [37]. The amino acid sequences and three dimensional structures of the homologous proteins used as templates for comparative modelling were obtained from the Protein Data Bank http://www.rcsb.org/pdb/. Initial amino acid sequence alignments between RPS4 proteins and templates were also obtained using the program FUGUE. FUGUE produces alignments by comparison of sequence and structural profiles.

Theoretical models of the structures of the RPS4 proteins were obtained by the program MODELLER [38]. MODELLER generates protein structures by satisfaction of spatial restraints with simultaneous optimisation of CHARMM energies, conjugate gradients and molecular dynamics with simulated annealing. Comparative models were validated with PROCHEK [39] and WHAT_CHECK [40] that study the geometry of the structure of the protein, VERIFY3D [41] that reports amino acid environmental errors and JOY [42] for comparison of the environments of residues of the modelled structure with the environments of residues of the homologous proteins used for modelling. The alignments were manually modified when the model was unsatisfactory (i.e. produced results below validation program recommendations), and the modelling and validation processes repeated. These processes were repeated until models with good geometry and conformation were obtained. The program MODELLER was also used for loop remodelling.

The models of isolated RPS4X, RPS4Y1 and RPS4Y2 show no residues with disallowed conformation in the Ramachandran plot, with 90.2%, 90.7% and 90.7% residues, respectively, residues in the most favourable regions. The Verify3D report shows that 82.2% of residues of RPS4X, 92.0% of RPS4Y1 and 90.2% of RPS4Y2 have an averaged 3D-1D score > 0.2 and no negative values are observed (18). The WHAT_CHECK report shows that there were no 'bumps' between non-bonding atoms in the models of the three RPS4 proteins. For the WHAT_CHECK authors, two atoms are said to 'bump' if they are closer than the sum of their van der Waals radii minus 0.40Å (19). On the other hand, a WHAT_CHECK comparison of the backbone conformation with database proteins shows that the three backbone folds in this structure present a low conformation Z-score of -7.03 (RPS4X), -6.47 (RPS4Y1) and -7.05 (RPS4Y2) and that four, five and four side chains, respectively, can be flipped in order to form energetically more favourable hydrogen bonds. WHAT_CHECK reports similar errors for some of the template proteins used for modelling. In particular, WHAT_CHECK reports one bump, a low backbone conformation Z-score of -6.07 and three side chains that can be flipped for 1C06 and one bump and two side chains that can be flipped for 1JJ2 chain S.

Contact residues were defined as the residues that possessed an interface solvent accessible surface area (ASA) that decreased (ΔASA) by more than 1Å^2 ^on interaction [43]. The ASA was calculated using the Lee and Richards algorithm [44] developed by Richmond [45]. HBPLUS was used for hydrogen bond definition [46].

## Authors' contributions

AML conceived the study, designed and carried out all the experiments, interpreted the results and wrote the manuscript; RNM performed the protein comparative modelling and wrote part of the manuscript; NAA, CAS contributed to the experimental design, discussion of the results and with a critical review of the manuscript; PJE provided samples and contributed with a critical review of the manuscript; AA contributed to the discussion of the results and with a critical review of the manuscript. All authors read and approved the final manuscript.

## Supplementary Material

Additional file 1 Primers used in the expression studies. Click here for file

Additional file 2 Expression patterns of the 20 transcripts in a panel of 17 human tissues. Click here for file

Additional file 3 Alignment of the amino acid sequence of the three human RPS4 proteins. Click here for file

Additional file 4 BLAST alignment of the RPS4 protein family. Click here for file
