# Supplementary material for: The human RPS4 paralogue on Yq11.223 encodes a structurally conserved ribosomal protein and is preferentially expressed during spermatogenesis
Source: BMC Mol Biol. 2010 May 7;11:33. doi: 10.1186/1471-2199-11-33 (PMC2884166; doi:10.1186/1471-2199-11-33)
Supplement: Additional file 2 — Expression patterns of the 20 transcripts in a panel of 17 human tissues. [file 1471-2199-11-33-S2.PDF]

Table 1 – Expression patterns of the 20 transcripts in a panel of 17 human tissues (Ambion First Choice RNA Panel). Each tissue sample is derived from a pool of individuals (males and females). Bl – bladder, Br - brain, Co – colon, He – heart, Li – liver, Lu – lung, SI – small intestine, Th – thymus, Tc – trachea, Sk – skeletal muscle, Tr – thyroid, Kd – kidney, Es – esophagus, Sp - spleen, Pr – prostate, Te – testicle, Ov – ovary.

GENE	ISOFORM (Ensembl)	Bl.	Br.	Co.	He.	Li.	Lu.	SI.	Th.	Tc.	Sk.	Tr.	Kd.	Es.	Sp.	Pr.	Te.	Ov.
RPS4X	001- ENST00000316084	+	+	+	+	+	+	+	+	+	+	+	+	+	+	+	+	+
	002- ENST00000373626	-	+	-	+	-	-	+	+	-	-	+	-	-	+	-	-	+
RPS4Y1	001- ENST00000250784	-	+	-	+	-	-	+	+	+	-	+	+	-	+	-	+	-
	002- ENST00000430575	+	+	+	+	+	+	+	+	+	+	+	+	+	+	+	+	-
RPS4Y2	001- ENST00000288666	-	-	-	-	-	-	-	-	-	-	-	-	-	-	+	+	-
ZFX	001/002-<br>ENST00000379177<br>ENST00000304543	+	+	+	+	+	+	+	+	+	+	+	+	+	+	+	+	+
	003-ENST00000338565	-	+	+	-	-	-	+	-	-	+	+	-	+	-	+	-	+
ZFY	001-ENST00000383052	+	+	+	+	+	+	+	+	+	+	+	+	-	+	+	+	-
UTX	001- ENST00000377967	+	+	+	+	+	+	+	+	+	+	+	+	+	+	+	+	+
UTY	001- ENST00000331397	+	+	+	+	+	+	+	+	+	+	+	+	-	+	+	+	-
	002- ENST00000362096	+	+	+	+	+	+	+	+	+	+	+	+	-	+	+	+	-
	003- ENST00000329134	-	+	+	+	+	+	+	+	+	+	+	+	-	+	+	+	-
	004- ENST00000382893	+	+	+	+	+	+	+	+	+	+	+	+	-	+	+	+	-
SMCX	001 ENST00000375401	+	+	+	+	+	+	+	+	+	+	+	+	+	+	+	+	+
SMCY	001/002-<br>ENST00000317961<br>ENST00000382806	+	+	+	+	+	+	+	+	+	+	+	+	-	+	+	+	-
EIF1AY	001/003-<br>ENST00000361365<br>ENST00000382772	+	+	+	+	+	+	+	+	+	+	+	+	-	+	+	+	-
PRKX	001- ENST00000262848	+	+	+	+	+	+	+	+	+	+	+	+	+	+	+	+	+
PRKY	001- ENST00000362758	+	+	+	+	+	+	+	+	+	+	+	+	-	+	+	+	-
