# Supplementary figures and images for: The human RPS4 paralogue on Yq11.223 encodes a structurally conserved ribosomal protein and is preferentially expressed during spermatogenesis
Source: BMC Mol Biol. 2010 May 7;11:33. doi: 10.1186/1471-2199-11-33 (PMC2884166; doi:10.1186/1471-2199-11-33)

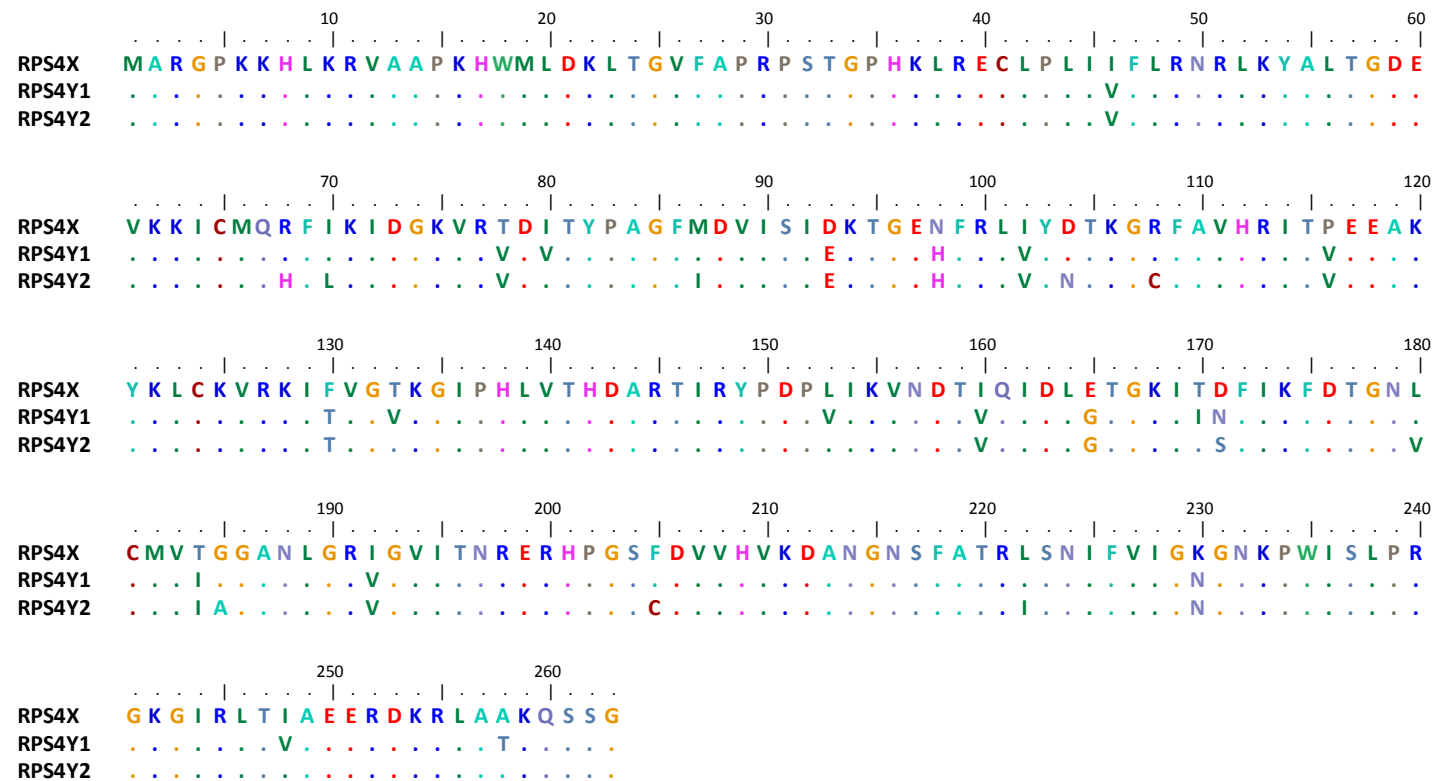

Supplement: Additional file 3 — Alignment of the amino acid sequence of the three human RPS4 proteins. [file 1471-2199-11-33-S3.PDF]
