# Supplementary material for: The human RPS4 paralogue on Yq11.223 encodes a structurally conserved ribosomal protein and is preferentially expressed during spermatogenesis
Source: BMC Mol Biol. 2010 May 7;11:33. doi: 10.1186/1471-2199-11-33 (PMC2884166; doi:10.1186/1471-2199-11-33)
Supplement: Additional file 4 — BLAST alignment of the RPS4 protein family. [file 1471-2199-11-33-S4.PDF]

Supplementary alignment

BLAST alignment in CLUSTAL format of the RPS4 family. Amino acid conservation is shown at the end of every part of the alignment. Key: '*' means absolutely conserved amino acid; ':' means conserved substitution; and '.' means less conserved substitution. Numbers correspond to the human RPS4X sequence.

```
CLUSTAL W (1.82) multiple sequence alignment

                     |    |10  |    |20  |                                 |30
RPS4X           MAR-GPKKHLKRVAAPKHWMLDKLTGVF-----------------------------APR
RPS4Y1          MAR-GPKKHLKRVAAPKHWMLDKLTGVF-----------------------------APR
RPS4Y2          MAR-GPKKHLKRVAAPKHWMLDKLTGVF-----------------------------APR
119592221       MAR-GPKKHLKRVAAPKHWMLDKLTGVF-----------------------------APR
4506725         MAR-GPKKHLKRVAAPKHWMLDKLTGVF-----------------------------APR
46048780        MAR-GPKKHLKRVAAPKHWMLDKLTGVF-----------------------------APR
12851918        MAR-GPKKHMKRVAAPKHWMLDKLTGVF-----------------------------APR
1350996         MAR-GPKKHLKRVAAPKHWMLDKLTGVF-----------------------------APR
57090063        MAR-GPKKHLKRVAAPKHWMLDKLTGVF-----------------------------APR
74179765        MAR-GPKKHLKRVAAPKHWMLDKLTGVF-----------------------------APR
62896517        MAR-GPKKHLKRVAAPKHWMLDKLTGVF-----------------------------APH
45360467        MAR-GPKKHLKRVAAPKHWMLDKLTGVF-----------------------------APR
126722859       MAR-GPKKHLKRVAAPKHWMLDKLTGVF-----------------------------APR
74136531        MAR-GPKKHLKRVAAPKHWMLDKLTGVF-----------------------------APR
227229          MAR-GPKKHLKRVAAPKHWMLDKLTGVF-----------------------------APR
950115          -AR-GPKKHLKRVAAPKHWMLDKLTGVF-----------------------------APR
147899358       MAR-GPKKHLKRVAAPKHWMLDKLTGVF-----------------------------APR
126327259       MAR-GPKKHLKRVAAPKHWMLDKLTGVF-----------------------------APR
119901281       MAR-GPKKHLKRVAAPKHWMLDKLTGVF-----------------------------APR
148229573       MAR-GPKKHLKRVAAPKHWMLDKLTGVF-----------------------------APR
114689109       MAR-GPKKHLKRVAAPKHWMLDKLTGVF-----------------------------APR
74136523        MAR-GPKKHLKRVAAPKHWMLDKLTGVF-----------------------------APR
119934106       MAR-GPKKHLKRVAAPKHWMLDKLTGVF-----------------------------APR
109083806       MAR-GPKKHLKRVVAPKHWMLDKLTGVF-----------------------------APR
57113929        MAR-GPKKHLKRVAAPKHWMLDKLTGVF-----------------------------APR
119895079       MAR-GPKKHLKRVAAPKHWMLDKLTGVF-----------------------------APR
29126987        MAR-GPKKHLKRVAAPRHWMLDKLTGVF-----------------------------APR
157819705       MAR-GPKKHLKRVAAPRHWMLDKLTGVF-----------------------------APR
53933236        MAR-GPKKHLKRVAAPKHWMLDKLTGVF-----------------------------APR
50403757        MAR-GPKKHLKRVAAPKHWMLDKLTGVF-----------------------------APR
72533642        MAR-GPKKHLKRVAAPKHWMLDKLTGVF-----------------------------APR
124300793       MAR-GPKKHLKRVAAPKHGMLDKLTGVF-----------------------------APR
4506727         MAR-GPKKHLKRVAAPKHWMLDKLTGVF-----------------------------APR
50401280        MAR-GPKKHLKRVAAPKHWMLDKLTGVF-----------------------------APR
133777261       -AR-GPKKHLKRVAAPKHWMLDKLTGVF-----------------------------APR
119930339       MAR-GPKKPLKCVAAPKHWMLDKLTGVF-----------------------------APR
119913177       MAQ-GPKKHLKPVAAPKHWMLDKLTGVF-----------------------------APR
38503307        MAR-GPKKHLKRVAAPKHWMLDKLTGVF-----------------------------APR
38503308        MAR-GPKKHLKRVAAPKHWMLDKLTGVF-----------------------------APR
119930307       MAR-GPKKHLKYVAAPKHWMLDELTGVF-----------------------------APR
62896747        MAR-GPKKHLKRVAAPKHWMLDKLTGVF-----------------------------APR
88703062        MAR-GPKKHLKRVAAPKHWMLDKLTGVF-----------------------------APR
57113861        MAR-GPKKHLKRVAAPKHWMLDKLTGVF-----------------------------APR
148680472       MTR-GPKKHLKRVAAPKHWILDKLTGVF-----------------------------APG
124486640       MTR-GPKKHLKRVAAPKHWILDKLTGVF-----------------------------APG
28204689        -AR-GPKKHLKRVAAPKHWMLDKLTGVF-----------------------------APR
28204681        -AR-GPKKHLKRVAAPKHWMLDKLTGVF-----------------------------APR
167045794       MAR-GSKKHLKRVAAPKHWMLDKLTGVF-----------------------------APR
119902798       MAR-GPKKPLKCVAAPKHWMLDKLTGVF-----------------------------APR
28204665        -AR-GPKKHLKRVAAPKHWMLDKLTGVF-----------------------------APR
76660997        MAR-GPKKHLKRVAAPKHWMLEKLTGVF-----------------------------ALR
149712494       MAR-GPKKHLKRLRAPKQWMLDKLTGVF-----------------------------APR
119923668       MAR-GPKKPLKCVAAPKHWMLDKLTGVF-----------------------------APR
90820002        MAR-GPKKHLKRLHAPKAWMLDKLGGVY-----------------------------APR
119923854       MAR-GPKKPLKCVAAPKHWMLDKLTGVF-----------------------------APR
70909479        MAR-GPKKHLKRLNAPKAWMLDKLGGVF-----------------------------APR
109067331       MAG-GPKKHLKRVAAPKHWMLDKLTGVF-----------------------------APR
125979105       MAR-GPKKHLKRLAAPKAWMLDKLGGAF-----------------------------APR
24663668        MAR-GPKKHLKRLAAPKAWMLDKLGGVF-----------------------------APR
146285351       MAR-GPKRHLKRLNAPKSWMLDKLGGVF-----------------------------APR
148691968       MAR-GPKKHLKRVAAPKHWMLDKLTGVF-----------------------------APS
170285559       MAR-GPRKHLKRLHAPKAWMLDKLGGVF-----------------------------APR
112984078       MAR-GPKKHLKRLNAPKAWMLDKLGGVY-----------------------------APR
91083095        MAR-GPKKHLKRLNAPKAWMLDKLGGVF-----------------------------APR
75029893        MAR-GPKKHLKRLNAPKSWMLDKLGGVF-----------------------------APR
74844658        MAR-GPKKHLKRLNAPKAWMLDKLGGVY-----------------------------APR
66517407        MAR-GPKKHLKRLNAPKAWMLDKLGGVY-----------------------------APR
70909489        MAR-GPKKHLKRLNAPKAWMLDKLGGVF-----------------------------APR
70909487        MAR-GPKKHLKRLNAPKAWMLDKLGGVF-----------------------------APR
156542863       MAR-GPKKHLKRLNAPKAWMLDKLGGVY-----------------------------APR
70909485        MAR-GPKKHLKRLNAPKAWMLDKLGGVF-----------------------------APR
440853          MAR-GPKKHLKRLAAPKAWMLDKLGGVF-----------------------------APR
74007700        -------------------MLDKLTGVF-----------------------------APR
74829226        MAR-GPKKHLKRLNAPKSWMLGKLGGVY-----------------------------APR
```

```
22138108        MAR-GLKKHLKRLNAPKHWMLDKLGGAF-----------------------------APK
125593989       -AR-GLKKHLKRLNAPSHWMLDKLGGAF-----------------------------APK
17979233        MAR-GLKKHLKRLNAPKHWMLDKLGGAF-----------------------------APK
118573852       MAR-GPKKHLKRLNAPKAWMLDKLGGVY-----------------------------APR
18415395        MAR-GLKKHLKRLNAPKHWMLDKLGGAF-----------------------------APK
1173256         MAR-GLKKHLKRLNAPRHWMLDKLGGAF-----------------------------APK
118480993       MAR-GLKKHLKRLNAPKHWMLDKLGGAF-----------------------------APK
118484853       MAR-GLKKHLKRLNAPKHWMLDKLGGAF-----------------------------APK
15237195        MAR-GLKKHLKRLNAPKHWMLDKLGGAF-----------------------------APK
18398393        MAR-GLKKHLKRLNAPKHWMLDKLGGAF-----------------------------APK
116871421       MAR-GPKKHLKRLAAPNHWMLDKLSGKF-----------------------------APR
73759787        MAR-GLKKHLKRLNAPKHWMLDKLGGAF-----------------------------APK
50550587        MAR-GPKKHLKRLAAPSHWMLDKLSGTY-----------------------------APR
45187482        MAR-GPKKHLKRLAAPHHWLLDKLSGCY-----------------------------APR
109067333       -------KHLKRVAAPKHWMLDKLTGVF-----------------------------APR
9759565         -AR-GLKKHLKRLNAPKHWMLDKLGGAF-----------------------------APK
48376549        ---------------------DKLTGVF-----------------------------APR
149287204       MAR-GPKKHLKRLHAPKSWMLDKLGGVF-----------------------------APR
82400118        MAR-GLKKHLKRLNAPKHWMLDKLGGAF-----------------------------APK
337930          -------------------MLDKLTGVF-----------------------------APR
159145754       -----SRKHLKRLNAPKHWMLDKMGGNF-----------------------------APR
1173257         MAR-GLKKHLKRLNAPKHWMLDKLGGAF-----------------------------APK
19112469        MVR-GPKKHLKRVAAPHHWLLDKLSGTY-----------------------------APK
148695326       -------------------MLDKLTGVF-----------------------------APR
50303511        MAR-GPKKHLKRLAAPHHWMLDKLSGCY-----------------------------APR
71000467        MPR-GPKKHQKRLSAPSHWLLDKMSGTY-----------------------------APK
19113142        MVR-GPKKHLKRVAAPHHWLLDKLSGTY-----------------------------APK
19115086        MVR-GPKKHLKRVAAPHHWLLDKLSGTY-----------------------------APK
168027041       MAR-GLKKHLKRLNAPKHWMLDKLGGAF-----------------------------APK
146413813       MAR-GPKKHLKRLAAPSHWMLDKLSGTY-----------------------------APR
164429760       MGR-GPKKHQKRLSAPSHWLLDKLSGVY-----------------------------APR
6321997         MAR-GPKKHLKRLAAPHHWLLDKLSGCY-----------------------------APR
3914899         MAR-GLKKHLKRLNAPKHWMLDKLGGAF-----------------------------APK
156362157       MAR-GPKKHMKRLNAPKHWMLDKLSGVF-----------------------------APR
119493023       MPR-GPKKHQKRLSAPSHWLLDKMSGTY-----------------------------APK
126139561       MGR-GPKKHLKRLAAPSHWMLDKLSGTY-----------------------------APR
145610060       MGR-GPKKHQKRLAAPSHWLLDKLSGTY-----------------------------APK
154273927       MGR-GPKKHQKRLSAPSHWLLDKLSGSY-----------------------------APR
126273970       MAR-GPKKHLKRLAAPSHWMLDKLSGTY-----------------------------APR
22758868        ------RKSMKRLTAPKSWMLDKLSGVF-----------------------------APR
3724352         --R-GPKKHLKRVAAPHHWLLDKLSGTY-----------------------------APK
119192028       MGR-GPKKHQKRLSAPSHWLLDKLSGAY-----------------------------APR
44894458        MAR-GLKKHLKRLNAPKHWMLDKLGGAF-----------------------------APK
68477663        MGR-GPKKHLKRLAAPSHWMLDKLSGTY-----------------------------APR
145245281       MPR-GPKKHQKRLSAPSHWLLDKMSGTY-----------------------------APK
116193489       MGR-GPKKHQKRLSAPSHWLLDKLSGVY-----------------------------APR
125552065       -AR-GLKKHLKRLNAPSHWMLDKLGGAF-----------------------------APK
115463485       MAR-GLKKHLKRLNAPSHWMLDKLGGAF-----------------------------APK
158187698       --R-GRRNHLKRLHAPKHWMLDKLGGVF-----------------------------APK
48596901        MAR-GLKKHLKRLNAPSHWMLDKLGGAF-----------------------------APK
2345154         MAR-GLKKHLKRLNAPKHWMLDKLGGAF-----------------------------APK
157690730       MVR-GPRKHMKRVAAPNHWMLDKLGGCY-----------------------------APR
168014621       MAR-GLKKHLKRLNAPRHWMLDKLGGAF-----------------------------APK
94468404        MAR-GPKKHLKRLNAPRGWMLDKTGGTF-----------------------------APR
108861826       --R-GNRKHLKRLNAPKHWMLDKLGGNF-----------------------------APR
115436514       MAR-GLKKHLKRLNAPKHWMLDKLGGAF-----------------------------APK
44967243        ----------RVAAPRHWMLDKLTGVF-----------------------------APR
115443689       MAR-GLKKHLKRLNAPKHWMLDKLGGAF-----------------------------APK
109509108       MAR-GPKKHLKRLNAPRGWMLDKTGGTF-----------------------------APR
168014095       MAR-GLKKHLKRLNAPRHWMLDKLGGAF-----------------------------APK
121704880       MPR-GPKKHQKRLSAPSHWLLDKMSGTY-----------------------------APK
168057168       MAR-GLKKHLKRLNAPKHWMLDKLGGAF-----------------------------APK
50287861        MAR-GPKKHLKRLAAPHHWLLDKLSGCY-----------------------------APR
167998342       MAR-GLKKHLKRLNAPRHWMLDKLGGAF-----------------------------APK
61654708        MPR-GPKKHLKRLHAPKHWMLDKLSGKF-----------------------------APR
29841450        --R-GNRKHLKRLNAPKHWMLDKLGGNF-----------------------------APR
170942476       MGR-GPKKHQKRLSAPSHWLLDKLSGVY-----------------------------APR
154290538       MGR-GPKKHQKRLSAPNHWLLDKLSGAY-----------------------------APR
168066840       MAR-GLKKHLKRLNAPKHWMLDKLGGAF-----------------------------APK
170034084       -AR-GPKKHLKRLNAPRGWMLDKTGGTF-----------------------------APR
170574388       MAR-GPKRHLKRLAAPKHWMLDKLGGVF-----------------------------APR
169595016       MGR-GPKKHQKRLSAPSHWLLDKLSGAY-----------------------------APK
158286707       MAR-GPKKHLKRLNAPRGWMLDKTGGTF-----------------------------APR
44967294        -----------RVAAPKHWMLDKLTGVF-----------------------------APR
156035911       MGR-GPKKHQKRLSAPNHWLLDKLSGAY-----------------------------APR
116779177       MAR-GLKKHLKRLNAPRHWMLDKLGGAF-----------------------------APK
119632119       -------------------MLDKLTGVF-----------------------------APR
170086746       MAR-GPKKHLKRLAAPSSWMLDKLSGTY-----------------------------APR
116781974       MAR-GLKKHLKRLNAPRHWMLDKLGGAF-----------------------------APK
149239787       MGR-GPKKHLKRLAAPSHWMLDKLSGAY-----------------------------APK
28630198        ----------RLAAPRHWMLDKLTGVF-----------------------------APR
50426545        MAR-GPKKHLKRLAAPSHWMLDKLSGTY-----------------------------APR
50423849        MGR-GPKKHLKRLAAPSHWMLDKLSGTY-----------------------------APR
167526949       MAR-GPKKHLKRLAAPKHWMLDKLTGRY-----------------------------APR
159466042       MVR-GPKKHLKRLNAPYHWMLDKLSGIF-----------------------------APK
82907488        MAR-GPKKHLKRVAAPRHWMLDKLTGIF-----------------------------A--
169849885       ----GDAQHLKRLAAPSSWMLDKLGGTY-----------------------------ASR
```

```
71015994        LVR-GPKHHLKRLNAPSSWMLDKLSGTY-----------------------------APR
119592222       MAR-GPKKHLKRVAAPKHWMLDKLTGVF-----------------------------APR
145286314       MAR-GPKKHLKRLATPKHWMLDKLGGVF-----------------------------AVR
149635430       LAR-GPKKHLKRVAAPKHWMLDKLTGVF-----------------------------APR
95007264        MAR-GPRKHLKRIAAPHHWMLDKLTGHY-----------------------------APR
1350992         MGR-GPKKHLKRLAAPSHWMLXKLSGTY-----------------------------APR
47208976        ------------------MLDKLTGVF-----------------------------APR
58259587        MGR-GPKKHLKRLAAPSSWMLDKLGGTY-----------------------------APR
28630195        -----------RLAAPKHWMLDKLTGVF-----------------------------APR
157337052       ------------------MLDKLGGAF-----------------------------APK
66823117        MAR-GPKKHLKRLAAPNHWMLDKLSGKW-----------------------------APR
17367482        MAR-GLKKHLKRLNAPKHWMLDKLGGAF-----------------------------APK
17543386        --R-GPKKHLKRLAAPSHWMLDKLGGVF-----------------------------AVR
157756381       --R-GPKKHLKRLAAPKHWMLDKLGGVF-----------------------------AVR
28630193        -----------RLAAPNHWMLDKLSGKF-----------------------------APR
79327186        ------------------MLDKLGGAF-----------------------------APK
157357316       ------------------MLDKLGGAF-----------------------------APK
10177019        ------------------MLDKLGGAF-----------------------------APK
145355247       MAR-GLKKHLKRLNAPKHWMLDKLGGVF-----------------------------APK
6598334         ------------------MLDKLGGAF-----------------------------APK
58257447        ------------------MLDKLSGTY-----------------------------APK
71029346        MGR-GPKKHMKRINAPSHWMLDKLTGRY-----------------------------APK
146418285       ------------------MLDKLSGTY-----------------------------APR
46128673        ------------------MLDKLSGTY-----------------------------APK
37779112        MAR-GPKKHLKRVAAPKHWMLDKLTGVF-----------------------------APR
67537248        ----------------------MSGTY-----------------------------APK
47777385        ------------------MLDKLGGAF-----------------------------APK
68475117        ------------------MLDKLSGTY-----------------------------APR
164656677       MVR-GPKHHLKHLAAPSSWMLDKLSGTY-----------------------------APR
115402491       ----------------------MSGTY-----------------------------APK
156088951       MGR-GVKKHMKRINAPSHWMLDKLSGRY-----------------------------APK
57047769        MAC-GPKKHLKCVAAPKHWMLDKLTGVF-----------------------------VPR
149244128       ------------------MLDKLSGAY-----------------------------APR
145528524       MAR-GPKKHMKRITAPKSWMLSKLGGNW-----------------------------TTR
84997205        ---------MKRINAPSHWMLDKLTGKY-----------------------------APK
126654348       -AR-GPKKHLKRVAAPSNWMLDKLTGVY-----------------------------APR
124803509       -GK-GIKKHLKRVNAPSHWMLNKMGGQY-----------------------------APK
167389537       -AR-GPRHHLKRLNAPHHWMLSKLGGTF-----------------------------APK
55982009        MAR-GPKKHLKRLNAPRGWMLDKTGGTF-----------------------------APR
70946979        -GK-GIKKHMKRVNAPSHWMLNKMDGQY-----------------------------APK
57107829        MAH-SPKKYVKRVAAPKPWMLDKLTGVF-----------------------------APR
159114046       MAR-GVRKHLKRLNAPKHWLLDKMGGIW-----------------------------APR
156098302       MGK-GIKKHMKRVNAPSHWMLNKMGGQY-----------------------------APK
67465453        MAR-GPRHHLKRLNAPHHWMLSKLGGTF-----------------------------APK
167391306       MAR-GPRHHLKRLNAPHHWMLSKLGGTF-----------------------------APK
68073433        -GK-GIKKHMKRVNAPSHWMLNKMDGQY-----------------------------APK
2463335         MAR-GLKKHLKRLNAPKHWMLDKLGGAF-----------------------------APK
73954159        MAH-SPRKHLKHVAAPKHWMLDKLTGVF-----------------------------APH
145475847       MAR-GPKKHMKRITAPKSWMLSKLGGNW-----------------------------TTR
115692154       MAR-GPKKHLKRLNAPKHWMLAKLTGNF-----------------------------APR
145488765       MAR-GPKKHMKRITAPKSWMLSKLGGNW-----------------------------TTR
118573853       -AR-GPKKHLKRINAPKSWMLNKLGGIW-----------------------------ATR
160550143       MAR-GPKKHLKRLNAPKHWMLDKLSGVF-----------------------------APR
146080976       MA----KKHLKRLYAPKDWMLSKLTGVF-----------------------------APR
145499397       MAR-GPKKHMKRITAPKSWMLSKLGGNW-----------------------------TTR
82539137        ---------MKRVNAPSHWMLNKMDGQY-----------------------------APK
56199522        -AR-GPKKHLKRLHAPKAWMLDKLGGVF-----------------------------APR
118376768       -AR-GPKKHLKRINAPKSWMLNKLGGIWVITFFAFRLRKNDFITTIPKLKFIYFKKQATR
154333974       ------KKHLKRLYAPKDWMLSKLTGVF-----------------------------APR
160331849       MAH-GIKKHLKRLRAPKRWKLTKLGGIW-----------------------------APN
169802405       MAR-GPRHHLKRLNAPHHWMLSKLGGTF-----------------------------APK
167379757       -----------RLNAPHHWMLSKLGGTF-----------------------------APK
109124282       VAF-GPKQHLKRVAALQHWMLDKLTGVF-----------------------------AAR
2500489         --------------------LDKLTGVF-----------------------------APR
157866320       ------KKHLKRLYAPKDWMLSKLTGVF-----------------------------APR
146080974       ------KKHLKRLYAPKDWMLSKLTGVF-----------------------------APR
123477532       MPR-CQRFHLKRLTAPHHWLLAKSAGKF-----------------------------ASH
4432939         --------------------LDKLTGVF-----------------------------APR
71755051        ------KKHLKRLYAPKDWMLSKLTGVF-----------------------------APR
123376214       MPR-CQRFHLKRLTAPHHWLLAKSAGKF-----------------------------ASH
145495133       ---------MKRITAPKSWMLSKLGGNW-----------------------------TTR
145500014       ---------MKRITAPKSWMLSKLGGNW-----------------------------TTR
71415246        ------KKHLKRLYAPKDWMLSKLTGVF-----------------------------APR
123439290       MPR-CQRFHLKRLTAPHHWLLAKSAGKF-----------------------------ASH
162606302       MSR-GIKKHYKRLNAPKKWLLNKLGGIW-----------------------------APR
67465619        ------------------MLSKLGGTF-----------------------------APK
167393127       ------------------MLSKLGGTF-----------------------------APK
148700270       MAR-GPKKHLKRVAAPKHWMLDKLTGVF-----------------------------APR
70909483        MAR-GPKKHLKRLNAPKAWMLDKLGGVF-----------------------------TPR
119623175       MAR-GPKKHLKRVAAPKHWMLDKLTGVF-----------------------------APR
57641464        MARKGPKRHLKRLAAPTSWYIHRKAYKW-----------------------------AVR
14591522        MARKGPKRHLKRLAAPSSWYIERKAYKW-----------------------------AVR
124028167       MARMGGRRHLKTLAAPKFWPVRQRAGIF-----------------------------TVK
15678046        MA---SRKHLKRFKSPVHWPIHPKEYKW-----------------------------TVK
14520545        MARKGPKRHLKRLAAPTSWYIERKAYKW-----------------------------AVR
                                           :                             .
```

```
                    |    |40  |                             |50  |    |60  |
RPS4X           PSTGPHKLRECLPLI-------------------------IFLRNRLKYALTGDEVKKIC
RPS4Y1          PSTGPHKLRECLPLI-------------------------VFLRNRLKYALTGDEVKKIC
RPS4Y2          PSTGPHKLRECLPLI-------------------------VFLRNRLKYALTGDEVKKIC
119592221       PSTGPHKLRECLPLI-------------------------IFLRNRLKYALTGDEVKKIC
4506725         PSTGPHKLRECLPLI-------------------------IFLRNRLKYALTGDEVKKIC
46048780        PSTGPHKLRECLPLI-------------------------IFLRNRLKYALTGDEVKKIC
12851918        PSTGPHKLRECLPLI-------------------------IFLRNRLKYALTGDEVKKIC
1350996         PSTGPHKLRECLPLI-------------------------IFLRNRLKYALTGDEVKKIC
57090063        PTTGPHKLRECLPLI-------------------------IFLRNRLKYALTGDEVKKIC
74179765        PSTGPHKLRECLPLI-------------------------IFLRNRLKYALTGDEVKKIC
62896517        PSTGPHKLRECLPLI-------------------------IFLRNRLKYALTGDEVKKIC
45360467        PSTGPHKLRECLPLI-------------------------IFLRNRLKYALTGDEVKKIC
126722859       PSTGPHKLRECLPLI-------------------------IFLRNRLKYALTGDEVKKIC
74136531        PSTGPHKLRECLPLI-------------------------IFLRNRLKYALTGDEVKKIC
227229          PSTGPHKLRECLPLI-------------------------IFLRNRLKYALTGDEVKKIC
950115          PSTGPHKLRECLPLI-------------------------IFLRNRLKYALTGDEVKKIC
147899358       PSTGPHKLRECLPLI-------------------------IFLRNRLKYALTGDEVKKIC
126327259       PSTGPHKLRECLPLI-------------------------IFLRNRLKYALTGDEVKKIC
119901281       PSTGPHKLRECLPLI-------------------------IFLRNRLKYALTGDEVKKIC
148229573       PSTGPHKLRECLPLI-------------------------IFLRNRLKYALTGDEVKKIC
114689109       PSTGPHKLRECLPLI-------------------------IFLRNRLKYALTGDEVKKIC
74136523        PSTGPHKLRECLPLI-------------------------IFLRNRLKYALTGDEVKKIC
119934106       PSTGPHKLRECLPLI-------------------------IFLRNRLKYALTGDEVKKIC
109083806       PSTGPHKLRECLPLI-------------------------IFLRNRLKYALTGDEVKKIC
57113929        PSTGPHKLRECLPLI-------------------------VFLRNRLKYALTGDEVKKIC
119895079       PSTGPHKLRECLPLI-------------------------IFLRNRLKYALTGDEVKKIC
29126987        PSAGPHRLRECLPLA-------------------------IFLRNRLKYALTGDEVKKIC
157819705       PSAGPHRLRECLPLA-------------------------IFLRNRLKYALTGDEVKKIC
53933236        PSTGPHKLRECLPLI-------------------------IFLRNRLKYALTGDEVKKIC
50403757        PSTGPHKLRECLPLI-------------------------IFLRNRLKYALTGDEVKKIC
72533642        PSTGPHKLRECLPLI-------------------------VFLRNRLKYALTGDEVKKIC
124300793       PSTGPHKLRECLPLI-------------------------IFLRNRLKYALTGDEVKKIC
4506727         PSTGPHKLRECLPLI-------------------------VFLRNRLKYALTGDEVKKIC
50401280        PSTGPHKLRECLPLI-------------------------IFLRNRLKYALTGDEVKKIC
133777261       PSTGPHKLRECLPLI-------------------------VFLRNRLKYALTGDEVKKIC
119930339       PSTGPHKLRECLPLI-------------------------TFLRNRLKYALTGDEVKKIC
119913177       PSTGPHKLRECLPLI-------------------------IFLRNRLKYALTGDEVKKMC
38503307        PSTGPHKLRECLPLI-------------------------VFLRNRLKYALTGDEVKKIC
38503308        PSTGPHKLRECLPLI-------------------------VFLRNRLKYALTGDEVKKIC
119930307       PSTGPHKLRECLPLF-------------------------IFLRNRLKYALTGDEVKKIC
62896747        PSTGPHKLRECLPLI-------------------------VFLRNRLKYALTGDEVKKIC
88703062        PSTGPHKLRECLPLI-------------------------VFLRNRLKYALTGDEVKKIC
57113861        PSTGPHKLRECLPLI-------------------------VFLRNRLKYALTGDEVKKIC
148680472       PSTGPHKLRECLPLI-------------------------IFLRNRLKYALNGDEVKKIC
124486640       PSTGPHKLRECLPLI-------------------------IFLRNRLKYALNGDEVKKIC
28204689        PSTGPHKLRECLPLI-------------------------VFLRNRLKYALTGDEVKKIC
28204681        PSTGPHKLRECLPLI-------------------------VFLRNRLKYALTGDEVKKIC
167045794       PSTSPHKLRECLPLI-------------------------IFLRNRLKYALTGDEVKKIC
119902798       PSTGPHKLRECLPLI-------------------------TFLRNRLKYALTGDEVKKIG
28204665        PSTGPHKLRECLPLI-------------------------VFLRNRLKYALTGDEVKKIC
76660997        PSAGPHKLRECLPLI-------------------------IFLRNRLKCALTGDEVKKIC
149712494       SSTRPHKLRECLPVV-------------------------IFLRNRLKYGLTGDEVKKIC
119923668       PSTGPHKLRECLPLI-------------------------TFLRNRLKYALTGDEVKKIG
90820002        PSTGPHKLRESLPLV-------------------------IFLRNRLKYALTNCEVKKIV
119923854       PSTGPHKLRECLPLI-------------------------TFLRNRLKYALTGDEVKKIG
70909479        PSTGPHKLRESLPLV-------------------------IFLRNRLKYALTNCEVTKIV
109067331       PSTGPHKLRECLPLI-------------------------IFLRNRLKYALTGDEVKKIC
125979105       PSTGPHKLRESLPLL-------------------------IFLRNRLKYALNGAEVTKIV
24663668        PSTGPHKLRESLPLL-------------------------IFLRNRLKYALNGAEVTKIV
146285351       PSTGPHKLRECLPLV-------------------------IFLRNRLKYALTKDEVTKIV
148691968       PSTGPHKLRECLPPI-------------------------IFLRNRLKYALTGDEVKKIC
170285559       PSTGPHKLRECLPLV-------------------------IFLRNRLKYALTNSEVTKIV
112984078       PSTGPHKLRECLPLV-------------------------IFLRNRLKYALTGNEVLKIV
91083095        PSTGPHKLRESLPLV-------------------------IFLRNRLKYALTNSEVTKIV
75029893        PSTGPHKLRESLPLI-------------------------IMLRNRLKYALTGAEVKKIV
74844658        PSTGPHKLRECLPLV-------------------------IFLRNRLKYALTGNEVLKIV
66517407        PSTGPHKLRESLPLV-------------------------IFLRNRLKYALTNSEVTKIV
70909489        PSTGPHKLRESLPLV-------------------------IFLRNRLKYALTNCEVKKIV
70909487        PSTGPHKLRESLPLV-------------------------IFLRNRLKYALTNSEVTKIV
156542863       PSTGPHKLRESLPLV-------------------------IFLRNRLKYALTNCEVTKIV
70909485        PSTGPHKLRESLPLV-------------------------IFLRNRLKCALTNAEVTKIV
440853          PSTGPHKLRESLPLL-------------------------TFLRNRLKYALNGAEVTKIV
74007700        PSTGPHKLRECLPLI-------------------------IFLRNRLKYALTGDEVKKIC
74829226        PSTGPHKLRECLPLL-------------------------IFLRNRLKYALTNCEVTKIT
22138108        PSSGPHKSRECLPLI-------------------------LILRNRLKYALTYREVIAIL
125593989       PSSGPHKARECLPLI-------------------------LILRNRLKYALTYREVISIL
17979233        PSSGPHKSRECLPLV-------------------------LIIRNRLEYALTYREVISIL
118573852       PSTGPHKLRESLPLV-------------------------IFLRNRLKYALTNSEVTKIV
18415395        PSSGPHKSRECLPLV-------------------------LIIRNRLKYALTYREVISIL
1173256         PSSGPHKSRECLPLI-------------------------LILRNRLKYALTYREVIAIL
118480993       PSSGPHKSRECLPLI-------------------------LILRNRLKYALTYREVISIL
118484853       PSSGPHKSRECLPLI-------------------------LILRNRLKYALTGREVLAIL
```

15237195	PSSGPHKSRECLPLV-------------------------LIIRNRLKYALTYREVISIL
18398393	PSSGPHKSRECLPLV-------------------------LIIRNRLKYALTYREVISIL
116871421	PSCGPHKLRECLPLC-------------------------LFLRNRLKYALTYDEVKRIL
73759787	PSSGPHKSRECLPLI-------------------------LILRNRLKYALTYREVQSIL
50550587	SSAGPHKLRESLPLV-------------------------IFLRNRLKYALNGREVNAIL
45187482	PSAGPHKLRESLPLI-------------------------VFLRNRLKYALNGREVKAIL
109067333	PSTGPHKLRECLPLI-------------------------IFLRNRLKYALTGDEVKKIC
9759565	PSSGPHKSRECLPLV-------------------------LIIRNRLKYALTYREVISIL
48376549	PSTGPHKLRECLPLI-------------------------IFLRNRLKYALTGDEVKKIC
149287204	PSTGPHKLRESLPLI-------------------------IMLRNRLKYALTGAEVKKIV
82400118	PSSGPHKSRECLPLV-------------------------IIMRNRLKYALTYREVISIL
337930	PS-GPHKLRECLPLI-------------------------IFLRNRLKYALTGDEVKKIC
159145754	PSTGPHKLRECLPLI-------------------------LFIRNRLKYALTYDEAKKIM
1173257	PSSGPHKSRECLPLV-------------------------IIMRNRLKYALTYREVISIL
19112469	PSPGPHKARECLPLI-------------------------VFLRNRLKYALNGREVKAIL
148695326	PSTGPHKLRECPPLI-------------------------IFLRNRLKYALTGDEVKKIC
50303511	PSAGPHKLRESLPLI-------------------------VFLRNRLKYALNGREVKAIL
71000467	ASPGPHKLRDCLPLI-------------------------VFIRNRLKYALNGRETKAIM
19113142	PSPGPHKARECLPLI-------------------------VFLRNRLKYALNGREVKAIL
19115086	PSPGPHKARECLPLI-------------------------VFLRNRLKYALNGREVKAIL
168027041	PSPGPHKERECLPLV-------------------------VMLRNRLKYALTYREVVAIV
146413813	PSAGPHKLRESLPLV-------------------------VFLRNRLKYALNGREVKAIL
164429760	PSAGPHKLRECMPLI-------------------------VFVRNRLKYALNYRETKAIM
6321997	PSAGPHKLRESLPLI-------------------------VFLRNRLKYALNGREVKAIL
3914899	PSSGPHKSRECLPLI-------------------------LIIRNRLKYALTYREVISIL
156362157	PSTGPHKLRECLPLI-------------------------IFLRNRLKYALNGEEVKKIV
119493023	ASPGPHKLRDCLPLI-------------------------VFIRNRLKYALNGRETKAIM
126139561	PSAGPHKLRESLPLV-------------------------VFLRNRLKYALNGREVKAIL
145610060	PSPGPHKQRECLPLI-------------------------VFIRNRLKYALNGRETKAIL
154273927	PSPGPHKLRDCLPLI-------------------------VFIRNRLKYALNARETNAIL
126273970	PSAGPHKLRESLPLV-------------------------VFLRNRLKYALNGREVKAIL
22758868	PSTGPHKLRECLPMI-------------------------VFLRNRLKYALTYDEVKKIV
3724352	PSPGPHKARECLPLI-------------------------VFLRNRLKYALNGREVKAIL
119192028	PSPGPHKLRDCLPLI-------------------------VFIRNRLKYALNGRETNAIL
44894458	PSSGPHKSRECLPLI-------------------------LIIRNRLKYALTYREVISIL
68477663	PSAGPHKLRESLPLV-------------------------VFLRNRLKYALNGREVKAIM
145245281	ASPGPHKLRDCLPLI-------------------------VFIRNRLKYALNGRETKAIM
116193489	PSAGPHKLRDCMPLI-------------------------VFVRNRLKYALNYRETKAIM
125552065	PSSGPHKARECLPLI-------------------------LILRNRLKYALTYREVISIL
115463485	PSSGPHKARECLPLI-------------------------LILRNRLKYALTYREVISIL
158187698	PSTGPHKTRECLPMM-------------------------IFLRNRLKYALTYTEVKKIV
48596901	PSSGPHKARECLPLI-------------------------LILRNRLKYALTYREVQSIL
2345154	PSSGPHKSRECLPLI-------------------------LIIRNRLKYALNYREVISIL
157690730	PSSGPHKLRECLPLA-------------------------LFLRNRLKYALTYSEVKKIL
168014621	PSPGPHKERECLPLV-------------------------VMLRNRLKYALTYREVVAIV
94468404	PSTGPHKLRESLPLV-------------------------IFLRNRLKYALTNTEVTKIV
108861826	PSTGPHKLRECLPLC-------------------------IFLRNRLKYALTYTECTKIL
115436514	PSSGPHKSRECLPLI-------------------------LIIRNRLKYALTYREVISIL
44967243	PSTGPHKLRECLPLI-------------------------IFLRNRLKYALTGDEVKKIC
115443689	PSSGPHKSRECLPLI-------------------------LIIRNRLKYALTYREVISIL
109509108	PSTGPHKLRESLPLV-------------------------IFLRNRLKYALTNNEVTRIV
168014095	PSPGPHKERECLPLV-------------------------VMLRNRLKYALTYREVVAIV
121704880	ASPGPHKLRDCLPLI-------------------------VFIRNRLKYALNGRETKAIM
168057168	PSPGPHKERECLPLV-------------------------VMLRNRLKYALTYREVVAIV
50287861	PSAGPHKLRESLPLI-------------------------VFLRNRLKYALNGREVKAIM
167998342	PSPGPHKERECLPLV-------------------------VMLRNRLKYALTYREVVAIV
61654708	PSPGPHKLRECIPLV-------------------------VFLRNRLKYALTADEVKRIV
29841450	PSTGPHKLRECLPLC-------------------------ILLRNRLKYALTYTECTKIL
170942476	PSAGPHKLRDCMPLI-------------------------VFVRNRLKYALNFRETRAIL
154290538	PSAGPHKLRDCMPLI-------------------------VFIRNRLKYALNSRETKAIV
168066840	PSPGPHKERECLPLV-------------------------VMLRNRLKYALTYREVVAIV
170034084	PSTGPHKLRESLPLV-------------------------IFLRNRLKYALTNNEVTRIV
170574388	PRCGPHKLRESLPLI-------------------------LFLRNRLKYALTYNEARMIC
169595016	ASPGPHKLRDSLPLV-------------------------IFLRNRLKYALNAREVNAIL
158286707	PSTGPHKLRESLPLV-------------------------IFLRNRLKYALTNSEVTKIV
44967294	PSTGPHKLRECLPLI-------------------------IFLRNRLKYALTGDEVKKIC
156035911	ASAGPHKLRDCMPLI-------------------------VFIRNRLKYALNSRETKAIL
116779177	PSPGPHKGRECLPLV-------------------------VLIRNRLKYALTYREVIAIV
119632119	PSTGPHKLRECLPLI-------------------------VFLRNRLKYALTGDEVKKIC
170086746	PSPGPHKLRESLPLT-------------------------VFLRNRLKYALTGREVTAIV
116781974	PSPGPHKGRECLPLV-------------------------VLIRNRLKYALTYREVIAIV
149239787	SSAGPHKLRESLPLV-------------------------IFIRNRLKYALNGREVKAIL
28630198	PSTGPHKLRECLPLI-------------------------IFLRNRLKYALTGDEVKKIC
50426545	PSAGPHKLRESLPLV-------------------------IFLRNRLKYALNGREVKAIL
50423849	PSAGPHKLRESLPLV-------------------------IFLRNRLKYALNGREVKAIL
167526949	PSTGPHKLRECLPLV-------------------------IFLRNRLKYALTYHEVKMIL
159466042	PSAGPHKQRECLPLL-------------------------LILRNRLKYALTGKEVQSIL
82907488	-----HHLQECLPLA-------------------------IFLRNRPKYAMTGDEVKKIC
169849885	PSPGPHKLRESLPLT-------------------------VFLRNRLKYALTGREVTSIV
71015994	PSNGPHKLREALPLV-------------------------ILLRNRLKYALTGREVNAIT
119592222	PSTGPHKLRECLPLI-------------------------IFLRNRLKYALTGDEVKKIC
145286314	PRSGPHKLRECLPLN-------------------------LFLRNRLKYALNYAEVKKIL
149635430	PSTGPHKLRECLPLI-------------------------IFLRNRLKYALTGDEVKKIC

```
95007264        PSPGPHKLRESAPLV-------------------------VLLRNRLRYALTYREVMMIV
1350992         PSAGPHXLRESLPLX-------------------------VFLRNRLXYALCGREVKAIM
47208976        PSTGPHKLRECLPLI-------------------------IFLRNRLKYALTGDEVKKIC
58259587        PSPGPHKLRESLPLT-------------------------VFLRNRLKYALTGREVTAIV
28630195        PSTGPHRLRECLSLI-------------------------IFLRNRLRYALTYDEVKKIC
157337052       PSSGPHKSRECLPLI-------------------------LILRNRLKYALTYREVIAIL
66823117        PSSGPHKLRECLPLI-------------------------LVLRNRLKYALTKKEVTLIL
17367482        PSSGPHKSRECLPLI-------------------------LILRNRLKYALTYREVVSIL
17543386        PNPGPHKLRESLPLS-------------------------LFLRNRLKYALNYTEAKKIL
157756381       PNPGPHKLRESLPLS-------------------------LFLRNRLKYALNYTEAKKIL
28630193        PSCGPHKLRECLPLC-------------------------LFLRNRLKYALTYDEVKRIL
79327186        PSSGPHKSRECLPLV-------------------------LIIRNRLKYALTYREVISIL
157357316       PSSGPHKSRECLPLI-------------------------LILRNRLKYALTYREVIAIL
10177019        PSSGPHKSRECLPLV-------------------------LIIRNRLKYALTYREVISIL
145355247       PSPGPHKSRECLPLC-------------------------LILRNRLKYALTYKEVTTIL
6598334         PSSGPHKSRECLPLV-------------------------LIIRNRLKYALTYREVISIL
58257447        PSPGPHKQRECLPLI-------------------------VFIRNRLKYALNGRETKAIL
71029346        ASPGPHKSRECLPLL-------------------------VLLRNRLKYALTYDEVKLIV
146418285       PSAGPHKLRESLPLV-------------------------VFLRNRLKYALNGREVKAIL
46128673        PSAGPHKLRDCMPLI-------------------------VFIRNRLKYALNYREVKAIL
37779112        PSTGPHKLRECLPLI-------------------------IFLRNRLKYALTGDEVKKIC
67537248        ASPGPHKLRDCLPLI-------------------------VFIRNRLKYALNGRETKAIM
47777385        PSSGPHKARECLPLI-------------------------LILRNRLKYALTYREVISIL
68475117        PSAGPHKLRESLPLV-------------------------VFLRNRLKYALNGREVKAIM
164656677       PSTGPHKLREALPLVLCLRLAKLICEQGVALVLALAVVIWILLRNRLKYALTGREVLSIT
115402491       ASPGPHKLRDCLPLI-------------------------VFIRNRLKYALNGRETKAIM
156088951       PSPGPHKKTECLPLI-------------------------LLLRNRLKYALTYDEVKLIV
57047769        PSTGPHKLRECLPLI-------------------------IFLRNRLKYALTEDEVKKIC
149244128       SSAGPHKLRESLPLV-------------------------IFLRNRLKYALNGREVKAIL
145528524       PSQGPHKLRESIPLS-------------------------VILQQKLKYALYAREVQMIL
84997205        ASPGPHKSRECLPLL-------------------------VLLRNRLKYALTYDEVKLIV
126654348       PSSGPHKLRECIPLA-------------------------ILLRNRLKFALTYTEAKYIV
124803509       TSSGPHKLLESIPLV-------------------------ILLRNRLKYALTFDEVKMIL
167389537       PSHGPHGMKECLPLI-------------------------LILRNRLNYALNGREVTMIV
55982009        PSTGPHKLRESLPLV-------------------------IFLRNRLKYALTNTEVTKIV
70946979        TSSGPHKLLESIPLV-------------------------ILLRNRLKYALTFDEVKMIL
57107829        PSTGHHKLKECLPLI-------------------------IFLRNRLKYALTGDEVKKIW
159114046       PTNGPHGLRECIPLI-------------------------LILRNRLHYANTYAETSMIL
156098302       TSSGPHRLIESIPLV-------------------------ILLRNRLKYALTFDEVKMIL
67465453        PSHGPHGMKECLPLI-------------------------LILRNRLNYALNGREVTMIV
167391306       PSHGPHGMKECLPLI-------------------------LILRNRLNYALNGREVTMIV
68073433        TSSGPHKLLESIPLV-------------------------ILLRNRLKYALTFDEVKMIL
2463335         PSSGPHKSRECLPLI-------------------------LIIRNRLKYALTYREVISIL
73954159        PSTGPHKLRECLPLI-------------------------IFLRNRLKYALTGDEVKKIR
145475847       PSQGPHKLRESIPLS-------------------------VILQHKLKYALYARESTMIL
115692154       ASTGPHKLRECLPLI-------------------------IFLRNRLKYALTYVETKKIM
145488765       PSQGPHKLRESIPLS-------------------------VILQHKLKYALYARESTMIL
118573853       PSQGPHKLRESLPLS-------------------------VLLKERLNYALNGRDVTLIL
160550143       PSSGPHKLRESLPII-------------------------LFIRNRLKYALTYDEAKKIV
146080976       PRPGPHKLRECLPLL-------------------------VIIRNRLKYALNAREGEMIL
145499397       PSQGPHKLRESIPLS-------------------------VILQHKLKYALYAREVQTIL
82539137        TSSGPHKLLESIPLV-------------------------ILLRNRLKYALTFDEVKMIL
56199522        PSQGPHRLRESLPLV-------------------------IFLRNRLKYALTNSEVTKIV
118376768       PSQGPHKLRESLPLS-------------------------VLLKERLNYALNGRDVTLIL
154333974       PRPGPHKLRECLPLL-------------------------VIIRNRLKYALNAREGEMIL
160331849       PKSGPHKKFESFPLI-------------------------LILRNRLKYALNNQEAVQIL
169802405       PSHGPHGMKECLPLI-------------------------LILRNRLNYALNGREVTMIV
167379757       PSHGPHGMKECLPLI-------------------------LILRNRLNYALNGREVTMIV
109124282       PSTGPHKLRECLPH--------------------------IFLRNRLKYALTGDEVKKIC
2500489         PSTGPHKLRECLPLI-------------------------IFLRNRLKYALTGDEVKKIC
157866320       PRPGPHKLRECLPLL-------------------------VIIRNRLKYALNAREGEMIL
146080974       PRPGPHKLRECLPLL-------------------------VIIRNRLKYALNAREGEMIL
123477532       PSTGPHKLRECLPIN-------------------------IFLRDRLKYALTAKEAVAIV
4432939         PSTGPHKLRECLPLI-------------------------IFLRNRLKYALTGDEVKKIC
71755051        PRAGPHKLRECLSLL-------------------------IIIRNRLKYALNALEAQMIL
123376214       PSTGPHKLRECLPIN-------------------------IFLRDRLKYALTAKEAVAIV
145495133       PSQGPHKLRESIPLS-------------------------VILQQKLKYALYAREVQMIL
145500014       PSQGPHKLRESIPLS-------------------------VILQHKLKYALYAREVQMIL
71415246        PRAGPHKLRECMTLM-------------------------IIIRNRLKYALNAAEAQMIL
123439290       PSTGPHKLRECLPIN-------------------------IFLRDRLKYALTAKEAVAIV
162606302       SSSGPHKKQNSIPLV-------------------------IVIRNKLKYSTNQRESLNIL
67465619        PSHGPHGMKECLPLI-------------------------LILRNRLNYALNGREVTMIV
167393127       PSHGPHGMKECLPLI-------------------------LILRNRLNYALNGREVTMIV
148700270       PSTGPHKLRECLPLI-------------------------IFLRNRLKYALTGDEVKEIC
70909483        PSTGPHKLRESLPLV-------------------------IFLRNRLKYALTNSEVTKIV
119623175       PSTGPHKLRECLPLI-------------------------VFLRNRLKYALTGDEVKKIC
57641464        PSPGPHSMKTSIPLI-------------------------YIVRDYLGYAKTAREARKIL
14591522        PRPGPHNMRTSIPLL-------------------------YIVRDYLGYAKTAREARKIL
124028167       PSPGPHPIERSIPLL-------------------------ILVRDVLGYAKTAREARKLI
15678046        PSPGPHAIENSLPLM-------------------------IIVRDILKVADNAREARKII
14520545        PRPGPHNMRTSIPLL-------------------------YIVRDYLGYAKTAREARKIL
                     *    . .                            .::.    .    :   :
```

```
                               |70  |     |80  |            |90  |     |100 |     |110
RPS4X           --M--QR--FIKIDGKVRTDITYPAGF-M--------DVISIDKTGENFRLIYDTKGRFA
RPS4Y1          --M--QR--FIKIDGKVRVDVTYPAGF-M--------DVISIEKTGEHFRLVYDTKGRFA
RPS4Y2          --M--QH--FLKIDGKVRVDITYPAGF-I--------DVISIEKTGEHFRLVYNTKGCFA
119592221       --M--QR--FIKIDGKVRTDITYPAGF-M--------DVISIDKTGENFRLIYDTKGRFA
4506725         --M--QR--FIKIDGKVRTDITYPAGF-M--------DVISIDKTGENFRLIYDTKGRFA
46048780        --M--QR--FIKIDGKVRTDITYPAGF-M--------DVISIEKTGEHFRLVYDTKGRFA
12851918        --M--QR--FIKIDGKVRTDITYPAGF-M--------DVISIDKTGENFRLIYDTKGRFA
1350996         --M--QR--FIKIDGKVRTDITYPAGF-M--------DVISIDKTGENFRLIYDTKGRFA
57090063        --M--QR--FIKIDGKVRTDITYPAGF-M--------DVISIDKTGENFRLIYDTKGRFA
74179765        --M--QR--FIKIDGKVRTDITYPAGF-M--------DVISIDKTGENFRLIYDTKGRFA
62896517        --M--QR--FIKIDGKVRTDITYPAGF-M--------DVISIDKTGENFRLIYDTKGRFA
45360467        --M--QR--FIKIDGKVRTDITYPAGF-M--------DVISIEKTGEHFRLVYDTKGRFA
126722859       --M--QR--FIKIDGKVRTDVTYPAGF-M--------DVISIDKIGENFRLIYDTKGRFA
74136531        --M--QR--FIKIDGKVRTDITYPAGF-M--------DVISIEKTGEHFRLVYDTKGRFA
227229          --M--QR--FIKIDGKVRTDITYPAGF-M--------DVISIDKTGENFRLIYDTKGRFA
950115          --M--QR--FIKIDGKVRTDITYPAGF-M--------DVISIDKTGENFRLIYDTKGRFA
147899358       --M--QR--FIKIDGKVRTDITYPAGF-M--------DVISIEKTGEHFRLVYDTKGRFA
126327259       --M--QR--FIKIDGKVRTDITYPAGF-M--------DVISIEKTGEHFRLVYDTKGRFA
119901281       --M--QR--FIKIDGKVRTDITYPAGF-M--------DVISIDKTGENFHLIYDTKGRFA
148229573       --M--QR--FIKIDGKVRTDITYPAGF-M--------DVISIEKTGEHFRLVYDTKGRFA
114689109       --M--QR--FIKIDGKVRTDITYPAGF-MVTGYCLISDVISIDKTGENFRLIYDTKGRFA
74136523        --M--QR--FIKIDGKVRTDTTYPVGF-M--------DVISIEKTGEHFRLVYDTKGRFA
119934106       --M--QR--FIKIDGKVRTDITYPAGF-M--------DVIRTDKTGENFRLIYDTTGRFA
109083806       --M--QR--FIKIDGKVRTDITYPAGFIM--------DIISIDKTGENFRLIYDTKGRFA
57113929        --M--QR--FIKIDGKVRVDITYPAGF-M--------DVISIEKTGEHFRLVYDTKGRFA
119895079       --M--QR--FIKIDGKVRTDITYPAGF-M--------DVIRTDKTGENFRLIYDTTGRFA
29126987        --M--QR--LIKVDGKVRTDVAYPAGF-M--------DVISIDKSGENFRLVYDTKGRFA
157819705       --M--QR--LIKVDGKVRTDVAYPAGF-M--------DVISIDKSGENFRLVYDTKGRFA
53933236        --M--QR--FIKIDGKVRTDITYPTGF-M--------DVVSIEKTGENFRLIYDVKGRFT
50403757        --M--QR--FIKIDGKVRVDVTYPAGF-M--------DVISIEKTGEHFRLVYDTKGRFA
72533642        --M--QR--FIKIDGKVRVDVTYPAGF-M--------DVISIEKTGEHFRLVYDTKGRFA
124300793       --M--QR--FIKIDGKVRTDITYPAGF-M--------DVISIEKTGEYFRLIYDVKGRFT
4506727         --M--QR--FIKIDGKVRVDVTYPAGF-M--------DVISIEKTGEHFRLVYDTKGRFA
50401280        --M--QR--FIKVDGKVRTDITYPAGF-M--------DVISIEKTGENFRLIYDVKGRFT
133777261       --M--QR--FIKIDGKVRVDVTYPAGF-M--------DVISIEKTGEHFRLVYDTKGRFA
119930339       --M--QH--FIKIDGKVRTDITYPAGF-M--------DVIGIDKTGENFCLIYDTKGRFA
119913177       --M--QR--FIKIDGKVRTDITYPAGF-M--------DVISIDKTRENFRLIYDTKGRFA
38503307        --M--QR--FIKIDGKVRVDITYPAGF-M--------DVISIDKTGEHFRLVYDTKGRFA
38503308        --M--QR--FIKIDGKVRVDVTYPAGF-M--------DVISIEKTGEHFRLVYDTKGRFA
119930307       --M--QR--FTKIDGKVCTDITYPAGF-M--------DVISIDKTGENFHLIYDTKGRFA
62896747        --M--QR--FIKIDGKVRVDVTYPAGF-M--------DVISIEKTGEHFRLVYDTKGRFA
88703062        --M--QH--FLKIDGKVRVDITYPAGF-I--------DVISIEKTGEHFRLVYNTKGCFA
57113861        --M--QR--FIKIDGKVRVDVTYPAGF-M--------DVISIEKTGEHFRLVYDTKGRFA
148680472       --V--QR--FIKIDGKVRTDITYPAGF-M--------DVISIDKNRENFRLIYDTKGRFA
124486640       --V--QR--FIKIDGKVRTDITYPAGF-M--------DVISIDKNRENFRLIYDTKGRFA
28204689        --M--QR--FIKIDGKVRVDITYPAGF-M--------DVISIDKTGEHFRLVYDTKGRFA
28204681        --M--QR--FIKIDGKVRVDVTYPAGF-M--------DVISIEKTGEHFRLVYDTKGRFA
167045794       --M--QR--FIKIDGKVRTDITYPAGF-M--------DVISIDKTGENFHLIYDTKGRFA
119902798       --M--QH--FIKIDGKVRTDITYPAGF-M--------DVIGIDKTGENFRLIYDTKGRFA
28204665        --M--QR--FIKIDGKVRVDVTYPAGF-M--------DVISIEKTGEHFRLVYDTKGRFA
76660997        --M--QR--FIKIDGKVRTDITYPAGF-M--------DVISIDKTGENFRLIYDTKGHFA
149712494       --M--RR--FVKIDGKVRTDVTYPAGF-M--------DVISIDKTGENFRLIYDTKGRFA
119923668       --M--QH--FIKIDGKVRTDITYPAGF-M--------DVIGIDKTGENFRLIYDTKGRFA
90820002        --M--QR--LIKVDGKIRTDPNYPAGF-M--------DVVTIEKTGEFFRLIYDVKGRFT
119923854       --M--QH--FIKIDGKVRTDITYPAGF-M--------DVIGIDKTGENFRLIYDTKGRFA
70909479        --M--QR--LIKVDGKVRTDPNYPAGF-M--------DVITIEKTGEFFRLIYDVKGRFT
109067331       --M--QR--FIKIDGKVRTDITYPAGF-M--------DVISIDKTGENFRLIYDTKGRFA
125979105       --M--QR--LVKVDGKVRTDPTYPAGF-M--------DVITLEKTGEFFRLVYDVKGRFT
24663668        --M--QR--LVKVDGKVRTDPTYPAGY-M--------DVITLEKTGEFFRLVYDVKGRFV
146285351       --M--QR--LIKVDGKVRTDPNYPAGF-M--------DVITIEKTGEFFRLIYDVKGRYA
148691968       --M--QR--FIKIDGKIRTGFIYPAGF-M--------DVISIDKTGENFRLIYDTKGRFA
170285559       --M--QR--LIKIDSKVRTDINYPAGF-M--------DVITIEKTGEYFRLIYDVKGRFA
112984078       --K--QR--LIKVDGKVRTDPTYPAGF-M--------DVVSIEKTNELFRLIYDVKGRFT
91083095        --M--QR--LIKVDGKVRTDPNYPAGF-M--------DVVTIEKTGEFFRLIYDVKGRFT
75029893        --K--QR--LIKVDGKVRTDTNYPAGF-M--------DVVTIEKTGEFFRLLYDVKGRFT
74844658        --K--QR--LIKVDGKVRTDPTYPAGF-M--------DVVSIEKTNELFRLIYDVKGRFT
66517407        --M--QR--LIKVDGKVRTDANYPAGF-M--------DVITINKTGEYFRLIYDVKGRFT
70909489        --M--QR--LIKVDGKVRTDPNYPAGF-M--------DVVTIEKTSEFFRLIYDVKGRFT
70909487        --M--QR--LIKVDGKVRTDPNYPAGF-M--------DVVTIEKTGEFFRLIYDVKGRFT
156542863       --M--QR--LIKVDGKVRTDPNYPAGF-M--------DVITIEKTNEFFRLIYDVKGRFT
70909485        --M--QR--LIKVDGKVRTDPNYPAGF-M--------DVITIEKTGEYFRLIYDVKGRFT
440853          --M--QR--LVKVYGKVRTDPTYPAGY-M--------DVITLEKTGEFFRLVYDVKGRFV
74007700        --M--QR--FIKIDGKVRTDITYPAGF-M--------DVISIDKTGENFRLIYDTKGRFA
74829226        --M--QR--LIKVDGKVRTDSNYPLGF-M--------DVVTIEKTGEFFRLIYDVKGRFS
22138108        --M--QR--HVLVDGKVRTDKTYPAGF-M--------DVVSIPKTNENFRLLYDTKGRFR
125593989       --M--QR--HVMVDGKVRTDKTYPAGF-M--------DVVSIAKTGENFRLLYDTKGRFR
17979233        --M--QR--HIQVDGKVRTDKTYPAGF-M--------DVVSIPKTNENFRLLYDTKGRFR
118573852       --M--QR--LIKVDGKVRTDPNYPSGF-M--------DVITIEKTGEFFRLIYDVKGRFT
18415395        --M--QR--HIQVDGKVRTDKTYPAGF-M--------DVVSIPKTNENFRLLYDTKGRFR
1173256         --M--QR--HVMVDGKVRTDKTYPAGF-M--------DVVSIPKTNEDFRLLYDTKGRFC
118480993       --M--QR--HVLVDGKVRTDKTYPSGF-M--------DVVSIPKTNESFRLLYDTKGRFR
118484853       --M--QR--HVLVDGKVRTDKTYPSGF-M--------DVVSIPKTNESFRLLYDTKGRFR
```

15237195	--M--QR--HIQVDGKVRTDKTYPAGF-M--------DVVSIPKTNENFRLLYDTKGRFR
18398393	--M--QR--HIQVDGKVRTDKTYPAGF-M--------DVVSIPKTNENFRLLYDTKGRFR
116871421	--M--QR--LIKVDGKVRTDRCYPAGF-M--------DVITIEKTAENFRLIYDVKGRFT
73759787	--M--QR--HVLVDGKVRTDKTYPAGF-M--------DVVSIPKTNENFRLLYDTKGRFR
50550587	--M--QR--LVKVDGKVRTDSTFPAGF-M--------DVIQLEKTGENFRLVYDVKGRFA
45187482	--M--QR--HVKVDGKVRTDTTYPAGF-M--------DVITLEATNENFRLVYDVKGRFA
109067333	--M--QR--FIKIDGKVRTDITYPAGF-M--------DVISIDKTGENFRLIYDTKGRFA
9759565	--M--QR--HIQVDGKVRTDKTYPAGF-M--------DVVSIPKTNENFRLLYDTKGRFR
48376549	--M--QR--FIKIDGKVRTDITYPAGF-M--------DVISIDKTGENFRLIYDTKGRFA
149287204	--K--QR--LIKVDHKVRTDTNYPAGF-M--------DVITIEKTGEFFRLLYDVKGRFV
82400118	--M--QR--QVMVDGKVRTDKTYPAGF-M--------DVVSIPKTNENFRLLYDTKGRFR
337930	--M--QR--FIKIDGKVRTDITYPAGF-M--------DVISIDKTGENFRLIYDTKGRFA
159145754	--N--QR--LVKVDGKVRTDICYPSGF-M--------DVISIERTNENFRLVYDVKGRFA
1173257	--M--QR--QVMVDGKVRTDKTYPAGF-M--------DVVSIPKTNENFRLLYDTKGRFR
19112469	--M--QR--LIQVDGKVRTDSTFPTGF-M--------DVISVEKTGEHFRLVYDIKGRFT
148695326	--M--QR--FIKIDVKVRTDITYPAGF-M--------DVISIDKTGENFRLIYDTKGRFA
50303511	--M--QR--HVKVDGKVRTDTTFPAGF-M--------DVITLEATNENFRLVYDVKGRFA
71000467	--M--QR--LIKVDGKVRTDPTYPAGF-M--------DVISIEKTGENFRLIYDTKGRFT
19113142	--M--QR--LIKVDGKVRTDSTFPTGF-M--------DVISVEKTGEHFRLVYDIKGRFT
19115086	--M--QR--LIKVDGKVRTDSTFPTGF-M--------DVISVDKTGEHFRLVYDIKGRFT
168027041	--M--QR--LISIDGKVRTDKCYPAGF-M--------DVISIAKTNENFRLLYDNKGRFT
146413813	--M--QE--HVKVDGKVRTDSTFPAGF-M--------DVITLEATNEHFRLVYDVKGRFA
164429760	--M--QR--LIKVDGKVRTDITYPAGF-M--------DVITIEKTGENFRLIYDTKGRFT
6321997	--M--QR--HVKVDGKVRTDTTYPAGF-M--------DVITLDATNENFRLVYDVKGRFA
3914899	--M--QR--HVLVDGKVRTDKTYPAGF-M--------DVISIPKTNENYRLLYDTKGRFR
156362157	--K--QR--LIKIDGKVRTDTTYPAGF-M--------DVVTIDKTGENFRLLYDVKGRFA
119493023	--M--QR--LIKVDGKVRTDPTYPAGF-M--------DVIGIEKTGENFRLIYDTKGRFT
126139561	--M--QQ--HVKVDGKVRTDSTFPAGF-M--------DVISLEATNEHFRLIYDVKGRFA
145610060	--M--QR--LVKVDGKVRTDSTYPAGF-M--------DVVSIEKTGENFRLVYDTKGRFT
154273927	--M--QR--LVKVDGKVRTDSTYPAGF-M--------DVITIDKTGENFRLVYDTKGRFT
126273970	--M--QQ--HVKVDGKVRTDSTFPAGF-M--------DVISLEATNEHFRLVYDVKGRFA
22758868	--N--QR--LIKVDGKVRTAKDYPAGF-M--------DVITIEKTNENFRLLYDVKGRFT
3724352	--M--QR--LIKVDGKVRTDSTFPTGF-M--------DVISVDKTGEHFRLVYDIKGRFT
119192028	--M--QR--LVKVDGKVRTDATYPAGF-M--------DVISIEKTGENFRLIYDTKGRFT
44894458	--M--QR--HVLVDGKVRTDKTYPAGF-M--------DVISIPKTNENYRLLYDTKGRFR
68477663	--M--QQ--HVQVDGKVRTDTTYPAGF-M--------DVITLEATNEHFRLVYDVKGKFA
145245281	--M--QR--LIKVDGKVRTDPTYPAGF-M--------DVIGIEKTGENFRLIYDTKGRFT
116193489	--M--QR--LVKVDGKVRTDVTYPAGF-M--------DVITIEKTGENFRLVYDTKGRFT
125552065	--M--QR--HVMVDGKVRTDKTYPAGF-M--------DVVSIAKTGENFRLLYDTKGRFR
115463485	--M--QR--HVMVDGKVRTDKTYPAGF-M--------DVVSIAKTGENFRLLYDTKGRFR
158187698	--N--QR--LIKVDGKVRTDKTYPCGF-M--------DVITIEKTGENFRLIYDVKGRFA
48596901	--M--QR--HIMVDGKVRTDKTYPAGF-M--------DIISIPKTGENYRLLYDTKGRFR
2345154	--M--QR--HVLVDGKVRTDKTYPAGF-M--------DVISIPKTMENYRLLYDTKGRFR
157690730	--A--QR--LVKVDGRVRTDHTFPTGF-M--------DVVQMEKTGENFRLIYDVKGRYK
168014621	--M--QR--LISIDGKVRTDKCYPAGF-M--------DVISIAKTNENFRLLYDNKGRFT
94468404	--M--QR--HIKIDGKVRTDPNYPAGF-M--------DVITIEKTNENFRLIYDVKGRFT
108861826	--A--QR--LVKVDGRVRTDKKYPAGF-M--------DVITIEKTNEHFRLIYDTKGRFA
115436514	--M--QR--HVLVDGKVRTDKTYPAGF-M--------DVISIPKTGENYRLLYDTKGRFR
44967243	--M--QR--FIKIDGKVRTDITYPAGF-M--------DVISIEKTGEHFRLVYDTKGRFA
115443689	--M--QR--HVLVDGKVRTDKTYPAGF-M--------DVISIPKTGENYRLLYDTKGRFR
109509108	--M--QR--HIKIDGKVRTDINYPAGF-M--------DVITIDKTSENFRLIYDVKGRFT
168014095	--M--QR--LISIDGKVRTDKCYPAGF-M--------DVISIAKTNENFRLLYDNKGRFT
121704880	--M--QR--LIQVDGKVRTDPTYPAGF-M--------DVVGIEKTGENFRLIYDTKGRFT
168057168	--M--QR--LISIDGKVRTDKCYPAGF-M--------DVISIAKTNENFRLLYDNKGRFT
50287861	--M--QR--HVKVDGKVRTDATYPAGF-M--------DVITLEATNENFRLVYDVKGRFA
167998342	--M--QR--LISIDGKVRTDKCYPAGF-M--------DVISIAKTNENFRLLYDNKGRFT
61654708	--K--QR--LIKVDAKVRTDPTFPAGF-M--------DVVSIDKTGENFRLLYDIKGRFT
29841450	--A--QR--LVKVDGRVRTDKKYPAGF-M--------DVITIEKTNEHFRLIYDTKGRFA
170942476	--M--QR--LVKVDGKVRTDMTYPAGF-M--------DVISIEKTGENFRLVYDTKGRFT
154290538	--M--QR--LIKVDGKVRTDATYPAGF-M--------DVIGIEKTSENFRLVYDTKGRFT
168066840	--M--QR--LISIDGKVRTDKCYPAGF-M--------DVISIAKTNENFRLLYDNKGRFT
170034084	--M--QR--HIKIDGKVRTDINYPAGF-M--------DVITIDKTSENFRLIYDVKGRFT
170574388	--K--QR--LIKVDGKVRTDMRFPAGF-M--------DVIRIEKTNETFRLLYDAKGRYA
169595016	--M--QR--LVKVDGKVRTDSTFPSGL-M--------DVISIEKTGENFRLIYDTKGRFT
158286707	--M--QR--HVKIDGKVRTDPNYPAGF-M--------DVINIHKTGEYFRLIYDVKGRFT
44967294	--M--QR--FIKIDGKVRTDITYPSGF-M--------DVISIEKTGEHFRLVYDTKGRFA
156035911	--M--QR--LIKVDGKVRTDATYPAGF-M--------DVIGIEKTSENFRLVYDTKGRFT
116779177	--M--QR--LISVDGKVRTDKCYPAGF-M--------DVLSIAKTNENFRLLYDAKGRFR
119632119	--M--QH--FLKIDGKVRVDITYPAGF-I--------DVISIEKTGEHFRLVYNTKGCFA
170086746	--K--QR--LIKIDGKVRTDPTYPAGF-M--------DVISIEKSGEHFRLLYDVKGRFT
116781974	--M--QR--LISVDGKVRTDKCYPAGF-M--------DVLSIAKTNENFRLLYDAKGRFR
149239787	--M--QQ--HVQVDGKVRTDATFPAGF-M--------DVITLEATNEHFRLIYDVKGRFT
28630198	--M--QR--FIKIDGKVRTDITYPTGS-M--------DVISIDKTSEHFRLIYDTKGRFA
50426545	--M--QE--HVKVDGKVRTDATFPAGF-M--------DVITLEATNEHFRLIYDVKGRFT
50423849	--M--QE--HVKVDGKVRTDATFPAGF-M--------DVITLEATNEHFRLIYDVKGRFT
167526949	--K--QR--KVYVDGKARTDLTFPTGL-M--------DVISIPDTNEHFRLLFDTKGRYT
159466042	--M--QR--LVKVDGKVRTDHTYPTGF-M--------DVISMEKTDENFRLVLDTKGRFV
82907488	--M--QR--LIKVEGKVRTDVAYPVGF-M--------DVISMYKSGENFLLIYDTKGRFA
169849885	--K--QR--LIKIDGKVRTDPTYPAGF-M--------DVISIEKSGEHFRLLYDVKGRFT
71015994	--A--QR--LIKIDGKVRTDPTYPTGF-Q--------DVVSIEKSGEHFRILYDVKGRFI
119592222	--M--QR--FIKIDGKVRTDITYPAGF-M--------DVISIDKTGENFRLIYDTKGRFA
145286314	--R--QR--VIKVDGKIRTDHKFPAGF-M--------DVISIERTNETFRLLYDVKGRYT
149635430	--M--QR--FIKIDGKVRTDITYPAGF-M--------DVISIEKTGEHFRLVYDTKGRFA

```
95007264        --M--QR--LIKVDNKVRTDQCYPAGF-M--------DVISIEKTKENFRMLFDTKGRFV
1350992         --M--QQ--HVQVVGKVRTDTTYPAGF-M--------DVITLEATNEHFRLAYDVKGKFA
47208976        --M--QR--FIKIDGKVRTDITYPTGF-M--------DVISIDKTGEHFRLIYDVKGRFT
58259587        --K--QR--LIKVDGKVRTDETFPAGF-M--------DVISIERSGEHFRLLYDVKGRFT
28630195        --M--QR--LIKIDGKVRTDITYPAGF-M--------DVITIDKTSENFRLIYDTKGRFA
157337052       --M--QR--HVLVDGKVRTDKTYPSGF-M--------DVVSIPKTNENFRLLYDTKGRFR
66823117        --M--QR--LVKVDGKVRTDPNYPAGF-M--------DVISIEKTKENFRLLFDPKGRFT
17367482        --M--QR--HILVDGKIHFCIR----L-S--------DVVSIPKTNENFRLLYDTKGRFR
17543386        --T--QR--VVRVDGKVRTCHKFPTGF-M--------DVVAIERTNEYFRMLYDTKGRYV
157756381       --T--QR--VVRVDGKVRTCHKFPTGF-M--------DVVAIDRTNEYFRMLYDTKGRYI
28630193        --M--QR--LIKVDGKVRTDRCYPSGF-M--------DAITIEKTAENFRLICDVKGRFT
79327186        --M--QR--HIQVDGKVRTDKTYPAGF-M--------DVVSIPKTNENFRLLYDTKGRFR
157357316       --M--QR--HVLVDGKVRTDKTYPSGF-M--------DVVSIPKTNENFRLLYDTKGRFR
10177019        --M--QR--HIQVDGKVRTDKTYPAGF-M--------DVVSIPKTNENFRLLYDTKGRFR
145355247       --M--QR--VVKVDGKIRMDKCYPCGI-M--------DVVSIEKSDEHFRLIYDNKGRFV
6598334         --M--QR--HIQVDGKVRTDKTYPAGF-M--------DVVSIPKTNENFRLLYDTKGRFR
58257447        --M--QR--LVKVDGKVRTDSTYPAGF-M--------DVVSIEKTGENFRLVYDTKGRFT
71029346        --K--QR--LVKVDGKVRTDITYPTGF-M--------DVVSLDKTNEKFRMLYDTKGRFC
146418285       --M--QE--HVKVDGKVRTDSTFPAGF-M--------DVITLEATNEHFRLVYDVKGRFA
46128673        --M--QR--LVKVDGKVRTDSTFPSGF-M--------DVITIEKTGENFRLVYDTKGRFT
37779112        --M--QR--FIKIDGKVRTDVTYPAGF-M--------DVISIEKTGEHFRLIYDVKGRFT
67537248        --M--QR--LIQVDGKVRTDPTYPAGF-M--------DVITIEKTGENFRLIYDTKGRFT
47777385        --M--QR--HVMVDGKVRTDKTYPAGF-M--------DVVSIAKTGENFRLLYDTKGRFR
68475117        --M--QQ--HVQVDGKVRTDTTYPAGF-M--------DVITLEATNEHFRLVYDVKGKFA
164656677       --Q--QR--IIKVDNKVRTDPTFPTGF-M--------DVVSIEKSGEHFRILYDVKGRFV
115402491       --M--QR--LIKVDGKVRTDPTYPAGF-M--------DVIGIEKTGENFRLIYDTKGRFT
156088951       --M--QK--VVRIDGKVRTDMTYPIGF-M--------DVVSLERTNEHFRLLYDTKGRFV
57047769        --M--QH--FIKIDGKVQTDITYPAGF-M--------DVISIDKTGENFHLIYDTKGHFA
149244128       --M--QQ--HVQVDGKVRTDATFPAGF-M--------DVITLEATNEHFRLIYDVKGRFT
145528524       ADK--DG--NIKVDGKVRSDFGYPVGL-Q--------DVITIDKTRESYRVLYDVQGKFI
84997205        --K--QR--LVKVDGKVRTDITYPTGF-M--------DVVSLDKTNEKFRMLYDTKGRFC
126654348       --M--QR--LIKVDGKVRTDICFPLGL-M--------DVVSIDRVGKNIRIMYDTKGRFV
124803509       --I--QK--IVKVDNKVRTDCTFPVGL-M--------DVIHITKSNEYFRLLYDIKGRFV
167389537       --K--NR--TIKIDGKIRTDTRYPVGF-M--------DVLSIPRTKENFRLMYNTKRRFC
55982009        --M--QR--HIKIDGKVRTDPNYPAGF-M--------DVITIEKTNENFRLIYDVKGRFT
70946979        --I--QK--IVKVDNKTRTDCTFPVGL-M--------DVIHITKSNEYFRLLYDVKGRFV
57107829        --M--QH--FIKIDGKVRTDTTYPAGF-M--------DVISIDKTGENFRLIYDTKGRFA
159114046       --Q--DK--NVLIDGKPRTDPTFPVGF-M--------DVFEIPKVHKTFRVLYDVKGRFT
156098302       --I--QK--IVKVDNKIRTDCTFPVGL-M--------DVIHITKSNEYFRLLYDIKGRFV
67465453        --K--NR--TIKIDGKIRTDTRYPVGF-M--------DVLSIPRTKENFRLMYNTKRRFC
167391306       --K--NR--TIKIDGKIRTDTRYPVGF-M--------DVLSIPRTKENFRLMYNTKRRFC
68073433        --I--QK--IVKVDNKTRTDCTFPVGL-M--------DVIHITKSNEFFRLLYDVKGRFV
2463335         --M--QR--HVLVDGKVRTDKTYPAGF-M--------DVISIPKTGENYRLLYDTKGRFR
73954159        --M--QH--FIKMDGK-------------------------------------DTKGRFA
145475847       ADK--DG--NIKVDGKVRTDSGYPVGL-Q--------DVITIDKTRESYRVLYDVQGKFI
115692154       --M--QR--LIKVDGKVRTDITYPAGF-M--------DVISINKTGENFRLIYNVKGRFV
145488765       ADK--DG--NIKVDGKVRTDSGYPVGL-Q--------DVITIDKTRESYRVLYDVQGKFI
118573853       --NDKEG--NVFVDQKVRRDKGYPTGL-M--------DVVRIEKTDQSFRILYDTKGRFV
160550143       --M--QR--LIKVDNKVRTDLTFPAGF-M--------DVISIEKTSENFRLIYDVKGRFA
146080976       --R--QG--LVHVDNHPRRDGKYPAGF-M--------DVVEIPKTGDRFRLMYDVKGRFA
145499397       ADK--DG--NIKVDGKVRSDFGYPVGL-Q--------DVITIDKTRESYRVLYDVQGKFI
82539137        --I--QK--IVKVDNKTRTDCTFPVGL-M--------DVIHITKSNEFFRLLYDVKGRFV
56199522        --M--QR--LIKVDGKVRTDSNFPAGF-M--------DVITIEKTNEYFRLIYDVKGRFT
118376768       --N--DKEQNVFVDGKVRRDKGYPTGL-M--------DVVRIEKTDQSFRILYDTKGRFV
154333974       --R--QG--LVHVDNHPRRDGKYPAGF-M--------DVIEIPKTGDRFRLLYDVKGRFA
160331849       --Q--QR--SIKVDKKIRTEKNYPAGL-M--------DIISIEKTEENFRLLVDTFGRFI
169802405       --K--NR--TIKIDGKIRTDTRYPVGF-M--------DVLSIPRTKENFRLMYNTKRRFC
167379757       --K--NR--TIKIDGKIRTDTRYPVGF-M--------DVLSIPRTKENFRLMYNTKRRFC
109124282       --M--QW--FIKVDGKIRTDNNLPYWI-M--------DVISIDKMGEDFCLIYGINRRFA
2500489         --M--QR--FIKIDGKVRTDITYPAGF-M--------DVISIDKTGENFRLIYDTKGRFA
157866320       --R--QG--LVHVDNHPRRDGKYPAGF-M--------DVVEIPKTGDRFRLMYDVKGRFA
146080974       --R--QG--LVHVDNHPRRDGKYPAGF-M--------DVVEIPKTGDRFRLMYDVKGRFA
123477532       --K--RR--LVKVDGKVRTNYRYPTGL-M--------DVIELGKSNELFRIIYDCKGRFC
4432939         --M--QR--FIKIDGKVRTDVTYPAGF-M--------DVISIDKIGENFRLIYDTKGRFA
71755051        --R--QG--LVCVDGKPRKDGKYPAGF-M--------DVVEIPKTGDRFRILYDVKGRFA
123376214       --K--RR--LVKVDGKVRTNYRYPTGL-M--------DVIELGKSNELFRIIYDCKGRFC
145495133       ADK--DG--NVKVDGKVRSDFGYPVGL-L--------DVITIDKTRESYRVLYDVQGKFI
145500014       ADK--DG--NIKVDGKVRSDFGYPVGL-Q--------DVITIDKTRESYRVLYDVQGKFI
71415246        --R--QG--LVCVDGKPRKDTKYPVGF-M--------DVVEIPRTGDRFRILYDVKGRFA
123439290       --K--RR--LVKVDGKVRTNYRYPTGL-M--------DVIGLGKSNELFRIIYDCKGRFC
162606302       --K--KN--EIYVDGKVRADKNFPAGV-M--------DVISIKTTKENYRVLYDNVGHFF
67465619        --K--NR--TIKIDGKIRTDTRYPVGF-M--------DVLSIPRTKENFRLMYNTKRRFC
167393127       --K--NR--TIKIDGKIRTDTRYPVGF-M--------DVLSIPRTKENFRLMYNTKRRFC
148700270       --M--QR--FIKIDGKIRTGIIYPAGF-M--------DVISIDKTGENFRLIYDTKGRFA
70909483        --M--QR--LIKVDGKIRTDPNYPAGF-M--------DVVTIEKTGEFFRLIYDDKGTVS
119623175       --M--QR--FIKIDGKVRVDVTYPAGF-M--------DVISIEKTGEHFRLVYDTKGRFA
57641464        --N--EG--KILVDGRVRKDYKFPVGI-M--------DVVSIPETGEHYRVLPNRIGKLI
14591522        --N--EG--KFLVDGKVRKDYKFPVGI-M--------DVVSIPETGEHYRVLPNRIGKLV
124028167       --A--EG--HFKIDGRVRRNYKYPVGF-M--------DVIEIVDTGEFYRVLPYPTRFFT
15678046        --N--SG--EVLVDGRPRKNYKFPVGF-M--------DVVSIPRTGDVYRVLPDERGRLV
14520545        --N--EG--KFLVDGRVRKDYKFPVGI-M--------DVVSIPETGEHYRVLPNRIGKLI
                            :  :
```

```
                         |    |120 |    |130 |    |140 |        |150
RPS4X               VHRITPEEAKYKLCKVRKIFVGTKGIPHLVTHDART--I--RYP----DP------L---
RPS4Y1              VHRITVEEAKYKLCKVRKITVGVKGIPHLVTHDART--I--RYP----DP------V---
RPS4Y2              VHRITVEEAKYKLCKVRKITVGTKGIPHLVTHDART--I--RYP----DP------L---
119592221           VHRITPEEAKYKLCKVRKIFVGTKGIPHLVTHDART--I--RYP----DP------L---
4506725             VHRITPEEAKYKLCKVRKIFVGTKGIPHLVTHDART--I--RYP----DP------L---
46048780            VHRITAEEAKYKLCKVRKIFVGTKGIPHLVTHDART--I--RYP----DP------L---
12851918            VHRITPEEAKYKLCKVRKIFVGTKGIPHLVTHDART--I--RYP----DP------L---
1350996             VHRITSEEAKYKLCKVRKIFVGTKGIPHLVTHDART--I--RYP----DP------L---
57090063            VHRITPEEAKYKLCKVRKIFVGTKGIPHLVTHDART--I--RYP----DP------L---
74179765            VHRITPEEAKYKLCKVRKILVGTKGIPHLVTHDART--I--RYP----DP------L---
62896517            VHRITPEEAKYKLCKVRKIFVGTKGIPHLVTHDART--I--RYP----DP------L---
45360467            VHRITAEEAKYKLCKVRKTWVGTKGIPHLVTHDART--I--RYP----DP------L---
126722859           VHRITPEEAKYKLCKVRKIFVGTKGIPHLVTHDART--I--RYP----DP------L---
74136531            VHRITAEEAKYKLCKVRKIFVATKGIPHLVTHDART--I--RYP----DP------L---
227229              VHRITPEEAKYKLCKVRKIFVGTKGIPHLVTHDART--I--RYP----DP------L---
950115              VHRITPEEAKYKLCKVRKIFVGTKGIPHLVTHDART--I--RYP----DP------L---
147899358           VHRITAEEAKYKLCKVRKTWVGTKGIPHLVTHDART--I--RYP----DP------L---
126327259           VHRITAEEAKYKLCKVRKIFVGTKGIPHLATHDART--I--RYP----DP------L---
119901281           VHRITPEEAKYKLCKVRKIFVGTKGIPHLVTHDART--I--RYP----DP------L---
148229573           VHRITSEEAKYKLCKVRKTWVGTKGIPHLVTHDART--I--RYP----DP------L---
114689109           VHRITPEEAKYKLCKVRKIFVGTKGIPHLVTHDART--I--RYP----DP------L---
74136523            VHRITAEEAKYKLCKVRKTFVGTKAIPHLVTHDART--I--RYP----DP------L---
119934106           VHRITPEEAKYKLCKVRKIFVGTKGIPHLVTHDART--I--RYP----DP------L---
109083806           VHRITPEEAKYKLCKVRKIFVGTKGIPHLVTHDART--I--RYP----DP------L---
57113929            VHRITVEEAKYKLCKVRKITVGTKGIPHLVTHDART--I--RYP----DP------L---
119895079           VHRITPEEAKYKLCKVRKIFVGTKGIPHLVTHDART--I--RYP----DP------L---
29126987            VHRITPEEAKYKLCKVRKVFVGTKGIPHLVTHDART--I--RYP----DP------L---
157819705           VHRITPEEAKYKLCKVRKVFVGTKGIPHLVTHDART--I--RYP----DP------L---
53933236            VHRITNEEAKYKLCKVRKILIGTKGIPHLVTHDART--I--RYP----DP------L---
50403757            VHRITAEEAKYKLCKVRKITVGTKGIPHLVTHDART--I--RYP----DP------V---
72533642            VHRITVEEAKYKLCKVRKITVGVKGIPHLVTHDART--I--RYP----DP------V---
124300793           VHRITAEEAKYKLCKVKKLLVGTKGIPHLVTHDART--I--RYP----DP------L---
4506727             VHRITVEEAKYKLCKVRKITVGVKGIPHLVTHDART--I--RYP----DP------V---
50401280            VHRITNEEAKYKLCKVKKILIGTKGIPHLVTHDART--I--RYP----DP------M---
133777261           VHRITVEEAKYKLCKVRKITVGVKGIPHLVTHDART--I--RYP----DP------V---
119930339           VHRITPQEAKYKLCKVRKIFVGTKGIPHLVTHDARA--I--RYP----DP------L---
119913177           VHRITPEEAKYKLCKVRKIFVGTKGIPHLVTHDARI--I--RYP----DP------L---
38503307            VQRITVEEAKYKLCKVRKITVGMKGIPHLVTHDART--I--RYP----DP------L---
38503308            VHRITVEEAKYKLCKVRKITVGVKGIPHLVTHDART--I--RYP----DP------V---
119930307           VHRITPEEAKYKLCKVRKIFVGTKGIPHLVTHDART--I--RYP----DP------L---
62896747            VHRITVEEAKYKLCKVRKITVGVKGIPHLVTHDART--I--RYP----DP------V---
88703062            VHRITVEEAKYKLCKVRKITVGTKGIPHLVTHDART--I--RYP----DP------L---
57113861            VHRITVEEAKYKLCKVRKITVGVKGIPHLVTHDART--I--RYP----DP------V---
148680472           VHRITLEEAQYKLCKVRKIFVGTKGIPHLVTHDGRT--I--RYP----DP------L---
124486640           VHHITLEEAQYKLCKVRKIFVGTKGIPHLVTHDGRT--I--RYP----DP------L---
28204689            VQRITVEEAKYKLCKVRKITVGMKGIPHLVTHDART--I--RYP----DP------L---
28204681            VHRITVEEAKYKLCKVRKITVGVKGIPHLVTHDART--I--RYP----DP------V---
167045794           VHRITPEEVKYKLCKVQKIFVGTKGIPHLVTPDAHT--I--RYH----DP------L---
119902798           VHRITPQEAKYKLCKVRKIFVGTKGIPHLVTHDARA--I--RYP----DP------L---
28204665            VHRITVEEAKYKLCKVRKITVGVKGIPHLVTHDART--I--RYP----DP------V---
76660997            VHRITPEEAKYKLYKVRKIFMGTKGIPHLVTHDART--I--RYP----DP------L---
149712494           VHRITPEEAKYKLCKVRKTFVGPKGIPHLVTHDART--I--RYP----DP------L---
119923668           VHRITPQEAKYKLCKVRKIFVGTKGIPHLVTHDARA--I--RYP----DP------L---
90820002            IHRITEEEAKYKLCKVKRVQTGPKGIPFLVTHDGRT--I--RYP----DP------L---
119923854           VHRITPQEAKYKLCKVRKIFVGTKGIPHLVTHDARA--I--RYP----DP------L---
70909479            IHRITAEEAKYKLCKVKKVQTGPKGIPFLVTHDGRT--I--RYP----DP------M---
109067331           VHRITPEEAKYKLCKVRKIFVGTKRIPHLVTHDAHT--I--HYP----DP------L---
125979105           IHRISAEEAKYKLCKVKKNQLGAKGVPFLVTHDGRT--I--RYP----DP------L---
24663668            IHRISAEEAKYKLCKVKKTQLGAKGVPFLVTHDGRT--I--RYP----DP------L---
146285351           IHRITADEAKYKLCKVRKVQTGPKGIPFLVTHDGRT--I--RYP----DP------L---
148691968           VHRITPEEAKYKLCKVRKIFVGTKGIPHLVTHDART--T--RYP----DP------L---
170285559           VHRITAEEAKYKLCKVRKVQTGPKGIPFIVTHDGRT--I--RYP----DP------V---
112984078           IHRITPEEAKYKLCKVKRVATGPKNVPYLVTHDGRT--I--RYP----DP------L---
91083095            IHRITGEEAKYKLCKVKKVQTGPKGIPFLVTRDGRT--I--RYP----DP------M---
75029893            LHRITPEEAKYKLCRVKRVQVGPKGVPFLTTHDART--I--RYP----DP------L---
74844658            VHRITPEEAKYKLCKVKRVSTGPKNVPFLVTHDGRT--I--RYP----DP------L---
66517407            THRITAEEAKYKLCKVKRVQTGPKGIPFLVTHDGRT--I--RYP----DP------V---
70909489            IHRITPEEAKYKLCKVRKVQTGPRGIPFLVTHDGRT--I--RYP----DP------V---
70909487            IHRITGEEAKYKLCKVKKVQTGPKGIPFLVTRDGRT--I--RYP----DP------N---
156542863           IHRITPVEAKYKLCKVRRVQTGPKGIPFLVTHDGRT--I--RYP----DP------L---
70909485            IHRITAEEAKYKLCKVRKVQTGPKGVPFLVTHDGRT--I--RYP----DP------I---
440853              IHRISAEEAKYKLCKVKKTQLGAKGVPFLVTHDGRT--I--RYP----DP------L---
74007700            VHRITPEEAKYKLCKVRKIFVGTKGIPHLVTHDART--I--RYP----DP------L---
74829226            IHRISNEEAKYKLCKVRRVQTGPKGVPFLVTHDGRT--L--RYP----DP------V---
22138108            LHSVRDDEAKFKLCKVRSVQFGQKGIPYLNTYDGRT--I--RYP----DP------L---
125593989           LHSIKDEDAKFKLCKVRSVQFGQKGIPFLNTNDGRT--I--RYP----DP------L---
17979233            LHSIKDEEAKFKLCKVRSIQFGQKGIPYLNTYDGRT--I--RYP----DP------L---
118573852           IHRITAEEAKYKLCKVKRVQTGPKGIPFLVTHDGRT--I--RYP----DP------I---
18415395            LHSIKDEEAKFKLCKVRSIQFGQKGIPYLNTYDGRT--I--RYP----DP------L---
1173256             LHAITGDETKFKLCKVRSVQFGQKGIPYLNTYDGRT--I--RYP----DP------L---
118480993           LHSLREDEAKFKLCKVRSIQFGQKGIPYLNTYDGRT--I--RYP----DP------L---
118484853           LHSLRDDEAKFKLCKVRSVQFGQKGIPYLNTYDGRT--I--RYP----DP------L---
```

15237195	LHSIKDEEAKFKLCKVRSIQFGQKGIPYLNTYDGRT--I--RYP----DP------L---
18398393	LHSIKDEEAKFKLCKVRSIQFGQKGIPYLNTYDGRT--I--RYP----DP------L---
116871421	IHRITPEEAKYKLCKVRKVMTGPKGIPCLVTHDART--I--RYP----DP------K---
73759787	LHSVRDEEAKFKLCKVRSVQFGQKGIPYINTYDGRT--I--RYP----DP------L---
50550587	VHRITDEEAAYKLGKVKRVQVGKKGIPYLVTHDGRT--I--RYP----DP------L---
45187482	VHRITDEEATYKLGKVKRVQLGKKGVPYVVTHDGRT--I--RYP----DP------N---
109067333	VHRITPEEAKYKLCKVRKIFVGTKRIPHLVTHDAHT--I--HYP----DP------L---
9759565	LHSIKDEEAKFKLCKVRSIQFGQKGIPYLNTYDGRT--I--RYP----DP------L---
48376549	VHRITPEEAKYKLCKVRKIFVGTKGIPHLVTHDART--I--RYP----DP------L---
149287204	LHRITPEEAKYKLCRVKRVQVGPKGVPFLVTHDART--I--RYP----DP------L---
82400118	LHSLRDEESKFKLCKVRSVQFGQKGIPYLNTYDGRT--I--RYP----DP------L---
337930	VHRITPEEAKYKLCKVRKIFVGTKGIPHLVTHDART--I--RYP----DP------L---
159145754	VHRISGEEAKYKLCKVRKVSVAPKGVPYLTTHDART--I--RYP----DP------L---
1173257	LHSLRDEESKFKLCKVRSVQFGQKGIPYLNTYDGRT--I--RYP----DP------L---
19112469	VHRITAEEAKYKLCKVKRVQLGAKGVPFLVTHDGRT--I--RYP----DP------L---
148695326	VHRITPEEAQYKLCKVRKIFVGTKGIPHLVTHDART--I--RYP----DP------L---
50303511	VHRITDEEASYKLAKVKKVQLGKKGIPYVVTHDGRT--I--RYP----DP------N---
71000467	VHRIQAEEAEYKLCKVKRVQLGKGGIPFLVTHDART--I--RYP----DP------A---
19113142	VHRITAEEAKYKLCKVKRVQLGAKGVPFLVTHDGRT--I--RYP----DP------L---
19115086	VHRITAEEAKYKLCKVKRVQLGAKGVPFLVTHDGRT--I--RYP----DP------L---
168027041	LHHISAEEAKYKLCKVRSAQFGDKGVPHITTFDGRT--I--RYP----DP------L---
146413813	VHRITAEEASYKLGKVKKVQLGKRGIPYVVTHDGRT--L--RYP----DP------L---
164429760	VHRIQDEEAKYKLGKVKRVQLGKGGIPFLVTHDART--I--RYP----DP------L---
6321997	VHRITDEEASYKLGKVKKVQLGKKGVPYVVTHDGRT--I--RYP----DP------N---
3914899	LHPIRDEDAKFKLCKVRSVQFGQKGIPYLNTYDGRT--I--RYP----DP------L---
156362157	VHRITAEEAKYKLGRVRRVDVGAKGVPYIVTHDART--I--RYP----DP------N---
119493023	VHRIQAEEAEYKLCKVKRVQLGKGGIPFLVTHDART--I--RYP----DP------A---
126139561	VHRITAEEASYKLGKVKKVQLGKRGVPYVVTHDGRT--L--RYP----DP------L---
145610060	VHRIQAEEAEYKLGKVKRVQLGRGGIPFLVTHDART--I--RYP----DP------L---
154273927	VHRIQAEEAEYKLGKVKRVQLGKGGIPFLVTHDART--I--RYP----DP------A---
126273970	VHRITAEEASYKLGKVKKVQLGKRGVPYVVTHDGRT--L--RYP----DP------L---
22758868	VHRIKPEEAKYKLCRVKRVTVGAKGVPMLTTHDART--I--RYP----DP------L---
3724352	VHRITAEEAKYKLCKVKRVQLGAKGVPFLVTHDGRT--I--RYP----DP------L---
119192028	VHRISGEEAEYKLGKVKRVQLGKGGIPFLVTHDART--I--RYP----DP------A---
44894458	LHPIRDEDAKFKLCKVRSVQFGQKGIPYLNTYDGRT--I--RYP----DP------P---
68477663	VHRISAEEAAYKLGKVKKVQLGKKGVPYVVTHDGRT--I--RYP----DP------L---
145245281	VHRIQAEEAEYKLCKVKRVQLGKGGIPFLVTHDART--I--RYP----DP------A---
116193489	VHRIQDEEAKYKLGKVKRVQLGRGGVPFLVTHDART--I--RYP----DP------L---
125552065	LHSIKDEDAKFKLCKVRSVQFGQKGIPFLNTNDGRT--I--RYP----DP------L---
115463485	LHSIKDEDAKFKLCKVRSVQFGQKGIPFLNTNDGRT--I--RYP----DP------L---
158187698	VHRISAEEAKYKLCKVKKGLVGLKGVPHLVTHDGRT--I--RYP----DP------L---
48596901	LHSVRDEDAKYKLCKVRSVQFGQKGIPYLNTNDGRT--I--RYP----DP------L---
2345154	LHPIRDEDAKFKLCKVRSVQFGQKGIPYLNTYDGRT--I--RYP----DP------L---
157690730	VHRISKEEALYKLCIVKKVSVGPKGIPYLTTTDGRT--I--RYP----DP------L---
168014621	LHNISAEEAKYKLCKVRSAQFGDKGVPYITTFDGRT--I--RYP----DP------L---
94468404	VHRITPEEAKYKLLKVKRVQIGPKKVPFILTHDGRT--I--RYP----DP------I---
108861826	IQRIHPDEAKYKLCKVKKVLVGHGGVPYIVTHDART--I--RYP----DP------N---
115436514	LQSVKDEDAKFKLCKVRSVQFGQKGIPYLNTYDGRT--I--RYP----DP------L---
44967243	VHRITAEETKYKLCKVRKVFVGTKGIPHLVTHDART--I--RYP----DP------L---
115443689	LQSVKDEDAKFKLCKVRSVQFGQKGIPYLNTYDGRT--I--RYP----DP------I---
109509108	VHRITPEEAKYKLLKVKRVQIGPKKVPFILTHDGRT--I--RYP----DP------I---
168014095	LHNISAEEAKYKLCKVRSAQFGDKGVPHITTFDGRT--I--RYP----DP------L---
121704880	VHRIQAEEAEYKLCKVKKVQLGKGGIPFLVTHDART--I--RYP----DP------A---
168057168	LHHISAEEAKYKLCKVRSAQFGDKGVPYITTFDGRT--I--RYP----DP------L---
50287861	VHRITDEEASYKLGKVKKVQLGKKGVPYVVTDDGRT--I--RYP----DP------N---
167998342	LHNISAEEAKYKLCKVRSAQFGDKGVPHITTFDGRT--I--RYP----DP------L---
61654708	VHRISPEEASYKLXKVRRIQLGKKGIPYCVTHDGRT--I--RYP----DP------V---
29841450	IQCIHPDEAKYKLCKVRKVLVGHGGVPFIVTHDART--I--RYP----DP------N---
170942476	VHRIGDEESKYKLGKVKRVQLGRGGVPFLVTHDART--I--RYP----DP------L---
154290538	VHRIQAEEAEYKLGKVKRVQLGKGGIPFLVTHDART--I--RYP----DP------S---
168066840	LHHISAEEAKYKLCKVRAAQFGDKGVPHVTTFDGRT--I--RYP----DP------L---
170034084	VHRITPEEAKYKLLKVKRVQIGPKKVPFILTHDGRT--I--RYP----DP------I---
170574388	THRITEQEGNIKLCKIVKKCVGPKGVPFIVTHDART--I--RYP----DP------H---
169595016	VHRITDEEAQYKLGKVKRVQLGKGGIPYLVTHDART--I--RYP----DP------A---
158286707	VHRITSEEAKYKLLKVKRVQIGPKRVPFLLTHDGRT--I--RYP----DP------Q---
44967294	VHRITAEEAKYKLCKVRKIFVRNKGIPHLVTHDART--I--RYP----DP------L---
156035911	VHRIQAEEAEYKLAKVKRVQLGKGGIPFLVTHDART--I--RYP----DP------A---
116779177	LHSIKDEEAKYKLCKVRSVQFGDKGIPYLNTYDGRT--I--RYP----DP------L---
119632119	VHRITVEEAKYKLCKVRKITVGTKGIPHLVTHDART--I--RYP----DP------L---
170086746	IHRITPEEANYKLLKVRRVAIGARGVPHIVTHDGRT--I--RYP----DP------L---
116781974	LHSIKDEEAKYKLCKVRSVQFGDKGIPYLNTYDGRT--I--RYP----DP------L---
149239787	VHRISAEEASYKLGKVKKVQLGKRGIPYVVTHDGRT--I--RYP----DP------L---
28630198	VHRITVEEAKYKLCKVRKIRVGTKGIPHLVTHDART--I--RYP----DP------L---
50426545	VHRITAEEASYKLAKVKKVQLGKRGIPYVVTHDGRT--I--RYP----DP------L---
50423849	VHRITAEEASYKLAKVKKVQLGKRGIPYVVTHDGRT--I--RYP----DP------L---
167526949	VHKIQDDEASFKLCKVKQAAIGPNAVPYVVTHDGRT--I--RYP----DP------H---
159466042	VHRISKEEAAYKLCRVRKVEFGKGGVPYVITHDGRT--I--RYP----DP------E---
82907488	VHRITPEEAKYKLCKVRKVFVGTKGIPHLITHDART--I--HYP----DP------L---
169849885	IHRITPEEATYKLLKVKRVALGARGVPHVVTHDGRT--I--RYP----DP------L---
71015994	VHRITAEEATYKLLKVKKVQLGARGVPHIVTHDGRT--I--RYP----DP------A---
119592222	VHRITPEEAKYKLCKVRKIFVGTKGIPHLVTHDART--I--RYP----DP------L---
145286314	VHRITAAEGQFKLCKVKALHVAKQNIPYITTNDGRT--I--RYP----DP------H---
149635430	VHRITAEEAKYKLCKVRKIFVGTKGIPHLVTHEART--I--RYP----DP------L---

```
95007264        PHPIREEEASYKLCRVKKVVVGPKGVPALITHDGRT--M--RYP----HP------S---
1350992         VHRISAEEAVYKLGKVKKVQLGKKGVPYVVTHDGRT--I--RYP----DP------L---
47208976        VHRITAEEAKYKLCKVKKIIIGTKGIPHLVTHDART--I--RYP----DP------L---
58259587        IHRITPEEATFKLLKVRKHQLGAKGVPHIVTHDGRT--I--RYP----DP------A---
28630195        IHRITAEEAKYKLCKIRKRRVASKGIPFVVTHDART--I--RYP----DP------L---
157337052       LHSIRDEEAKFKLCKVRSVQFGQKGIPYLNTYDGRT--I--RYP----DP------L---
66823117        LQRITPEEAKFKLARVTRVETGNQGIPYVHTDDGRT--I--RYP----DP------A---
17367482        LHSIRDEESKFKLCKVRSVQFGQKGIPYINTYDGRT--I--RYP----DP------L---
17543386        VHRIQAAEADFKLCKVKSVRTVNKGVPVLTTTDGRT--I--RYP----DP------H---
157756381       VHRIQAAEADFKLCKVKSVRTVIKGVPVLTTTDGRT--I--RYP----DP------H---
28630193        VHRITPEEAKYKLCKVRKVMTGPKGIPCLVTHDART--I--RYP----DP------K---
79327186        LHSIKDEEAKFKLCKVRSIQFGQKGIPYLNTYDGRT--I--RYP----DP------L---
157357316       LHSIRDEEAKFKLCKVKGVQFGQKGIPYLNTYDGRT--I--RYP----DP------L---
10177019        LHSIKDEEAKFKLCKVRSIQFGQKGIPYLNTYDGRT--I--RYP----DP------L---
145355247       VHRIEAIEAQYKLCKVKSKKLGDKGVPCVGLHDGRT--I--RYP----DP------L---
6598334         LHSIKDEEAKFKLCKVRSIQFGQKGIPYLNTYDGRT--I--RYP----DP------L---
58257447        VHRIQAEEAEYKLGKVKRVQLGRGGIPFLVTHDART--I--RYP----DP------L---
71029346        PHKITDEEATYKLCRVKKTFLGPKEVNLAVTHDGRT--F--RCV----HP------E---
146418285       VHRITAEEASYKLGKVKKVQLGKRGIPYVVTHDGRT--I--RYP----DP------L---
46128673        VHRIQNEEAEYKLGKVKRVQLGRGGIPFLVTHDART--I--RYP----DP------L---
37779112        VHRITGEEAKYKLCKVKKLIVGTKGIPHLVTHDART--I--RYP----DP------L---
67537248        VHRIQAEEAEYKLCKVKKVQLGKGGIPFLVTHDART--I--RYP----DP------A---
47777385        LHSIKDEDAKFKLCKVRSVQFGQKGIPFLNTNDGRT--I--RYP----DP------L---
68475117        VHRISAEEAAYKLGKVKKVQLGKKGVPYVVTHDGRT--I--RYP----DP------L---
164656677       VHRITAEEAQYKLLKVKRVQLGAKGVPLIVTHDGRT--L--RYP----DP------L---
115402491       VHRIQAEEAEYKLCKVKRVQLGKGGIPFLVTHDART--I--RYP----DP------A---
156088951       PHKITPEEASYKLCRVIKTFLGPKEVGMAVTHDGRT--I--RFV----HP------D---
57047769        VHRITPEEAKYKLCKVRKIFVGTKGIPHLVTHDAHT--I--CYP----DP------L---
149244128       VHRISAEEASYKLGKVKKVQLGKRGIPYVVTHDGRT--I--RYP----DP------L---
145528524       LKSIKPDEAKFKLVKVTQKKVGPNNVPYIVTNDSRT--I--RYP----NP------E---
84997205        PHKITDEEATYKLCRVKKTFLGPKEVNLAVTHDGRT--F--RCV----HP------E---
126654348       PVQIDSKEAGFKLCKVTKVALGAKGIPTATTNDART--L--RYI----HP------D---
124803509       PHRITNEESKYKLCKVKKILLRKGRLSIAVTHDGRS--I--PYI----HP------D---
167389537       LVPLTAEQAKFKLCKIEKRVLGTGAIPYIVTHDGRT--I--RYP----HP------E---
55982009        VHRITPEEAKYKLLKVKRVQIGPKKVPFILTHDGRT--I--RYP----DP------I---
70946979        PHRITNEESKYKLCKVKKIILRKGKLSIAITHDGRS--I--PYI----HP------E---
57107829        VHRITPEEAKYKLCKVRKIFVGTKGIPHLVTPDALT--T--GYP----DP------L---
159114046       LVPIQSNEAGFKLCRVQKIFLGDKGMPYLSTHDART--I--RFP----HP------D---
156098302       PHRITNEESKYKLCKVKKMILRKGRLSIAVTHDGRS--I--PYI----HP------D---
67465453        LVPLTAEQAKFKLCKIEKRVLGTGAIPYIVTHDGRT--I--RYP----HP------E---
167391306       LVPLTAEQAKFKLCKIEKRVLGTGAIPYIVTHDGRT--I--RYP----HP------E---
68073433        PHRITNEESKYKLCKVKKIILRKGKLSIAITHDGRS--I--PYI----HP------E---
2463335         LQSVKDEDAKFKLCKVRSVQFGQKGIPYLNTYVVPT--D--TYM----AT------IA--
73954159        VHRITPEEAKYKLGKVRKIFVGTKGIPHLVTHDART--I--RYP----DP------L---
145475847       LKSIKPEEAKFKLVKVTQKKVGPNNVPYIVTNDART--I--RYP----NP------D---
115692154       VHRIGSEEAKVRFVIHPRLTVSVFGAKHFTLGIGHS--G--TRR----NT------P---
145488765       LKSIKPEEAKFKLVKVTQKKVGPNNVPYIVTNDART--I--RYP----NP------D---
118573853       LKSLSKEEAKYKLLKVTAKAIGPNQIPYIVTHDSRT--I--RFP----NP------E---
160550143       IHRITAEEATYKLCKVAKQCTGPKGVPYVTTHDGRT--I--RYP----DP------L---
146080976       LVSLSEAEAQIKLMKVVNLYTATGRVPVAVTHDGHR--I--RYP----DP------H---
145499397       LKSIKPDEAKFKLVKVTQKKVGPNNVPYIVTSDART--I--RYP----NP------D---
82539137        PHRITNEESKYKLCKIKKIILRKGKLSIAITHDGRS--I--PYI----HP------E---
56199522        VHRISAEEAKYKLCKVKKTCVGPKKVPYLITHDGRT--I--RFP----DP------L---
118376768       LKSLSKEEAKYKLLKVTAKAIGPNQIPYIVTHDSRT--I--RFP----NP------E---
154333974       LVSVSEAEAQIKLMKVVNLYTATGRVPVAVTHDGHR--I--RYP----DP------H---
160331849       LHRIEEKESMFKLCKVTKSTKGQRGIPYIVTHDGRT--I--RFP----DP------F---
169802405       LVPLTAEQA--------KRVLGTGAIPYIVTHDGRT--I--RYP----HP------E---
167379757       LVPLTAEQAKFKLCKIEKRVLGTGAIPYIVTHDGRT--I--RYP----HP------E---
109124282       LHRITPEEVKYKLCKMRKIFVRAKGIPHLVIHDIHT--V--RHP----DS------L---
2500489         VHRITPEEAKYKLCKVRKIFVGTKGIPHLVTHDART--I--RYP----DP------L---
157866320       LVNLSEAEAQIKLMKVVNLYTATGRVPVAVTHDGHR--I--RYP----DP------H---
146080974       LVSLSEAEAQIKLMKVVNLYTATGRVPVAVTHDGHR--I--RYP----DP------H---
123477532       VHHIEAKEASFKLLRVNQFKIGAKGIPHVVTHDGRT--I--SYV----DP------S---
4432939         VHRITPEEAKYKLCKVRKIFVGTKGIPHLVTHDART--I--RYP----DP------L---
71755051        LVRVSEAESSIKMMKVVNVYTGTGRIPVAVTHDGHR--I--RYP----DP------R---
123376214       VHHIEAKEASFKLLRVNQFKIGAKGIPHVVTHDGRT--I--SYV----DP------S---
145495133       LKSIKPDEAKFKLVKVTQKKVGPNNVPYIVTNDART--I--RYP----NP------E---
145500014       LKSIKPDEAKFKLVKVTQKKVGPNNVPYIVTNDGRT--I--RYP----NP------E---
71415246        LVKVGEAEGNIKLLKVENVYTSTGRIPVAMTHDGHR--I--RYP----DP------R---
123439290       VHHIEAKEASFKLLRVNQFKIGAKGIPHVVTHDGRT--I--SYV----DP------S---
162606302       LHRITIKESMFKLCKVIRVSIGSKGIPYIVTHDGRT--I--RFP----NL------L---
67465619        LVPLTAEQAKFKLCKIEKRVLGTGAIPYIVTHDGRT--I--RYP----HP------E---
167393127       LVPLTAEQAKFKLCKIEKRVLGTGAIPYIVTHDGRT--I--RYP----HP------E---
148700270       VHRITPEEAKYKLCKVRKIFVGTKGIPHLVTHDALT--I--RYP----DP------L---
70909483        IHRITAEEAKYKLCKVRKVQTGPKGVPFLVTHDGKT--I--RYP----DP------M---
119623175       VHRITVEEAKYKLCKVRKITVGVKGIPHLVTHDART--I--RYP----DP------V---
57641464        LHPISEEEAKLKPFRINNKRMVKGAKVQLNLHDGSN--H--LVS----LA------EKDA
14591522        LHPISEEEANIKPLRIRNKRMVKGAKVQLNFHDGTN--H--LIPLTEKDN------Y---
124028167       LHPISKEEAQFKLGRIEDKSTVKGGHIQLHLHDGRNVLI--RVS----DP------TNPV
15678046        LHPIDEENAGFKLCKIVNKTTIKGGRTQLNLHDGRN--Y--L-S----DD------E---
14520545        LHPISEEEANIKPLRIRNKRMVKGAKIQLNFHDGTN--H--LIPLSEKDN------Y---
                    :   :
```

```
                         |    |160 |    |170 |    |180 |    |190 |    |200     |
RPS4X           ----IKVNDTIQIDLETGKITDFIKFDTGNLCMVTGGANLGRIGVITNRERH--P-G-SF
RPS4Y1          ----IKVNDTVQIDLGTGKIINFIKFDTGNLCMVIGGANLGRVGVITNRERH--P-G-SF
RPS4Y2          ----IKVNDTVQIDLGTGKITSFIKFDTGNVCMVIAGANLGRVGVITNRERH--P-G-SC
119592221       ----IKVNDTIQIDLETGKITDFIKFDTGNLCMVTGGANLGRIGVITNRERH--P-G-SF
4506725         ----IKVNDTIQIDLETGKITDFIKFDTGNLCMVTGGANLGRIGVITNRERH--P-G-SF
46048780        ----IKVNDTIQIDLETGKITDFIKFDTGNLCMVTGGANLGRIGVITNRERH--P-G-SF
12851918        ----IKVNDTIQIDLETGKITDFIKFDTGNLCMVTGGANLGRIGVITNRERH--P-G-SF
1350996         ----IKVNDTIQIDLETGKITDFIKFDTGNLCMVTGGANLGRIGVITNRERH--P-G-SF
57090063        ----IKVNDTIQIDLETGKITDFIKFDTGNLCMVTGGANLGRIGVITNREKH--P-G-SF
74179765        ----IKVNDTIQIDLETGKITDFIKFDTGNLCMVTGGANLGRIGVITNRERH--P-G-SF
62896517        ----IKVNDTIQIDLETGKITDFIKFDTGNLCMVTGGANLGRIGVITNRERH--P-G-SF
45360467        ----IKVNDTIQIDLETGKITDFIKFDTGNLCMVTGGANLGRIGVITNRERH--P-G-SF
126722859       ----IKVNDTIQIDLETGKITDFIKFDTGNLCMVTGGANLGRIGVITNRERH--P-G-SF
74136531        ----IKVNDTVQINLETGKITDFIKFDTGNLCMVTGGANLGRIGVITNREKH--P-G-SF
227229          ----IKVNDTIQIDLETGKITDFIKFDTGNLCMVTGGANLGRIGVITNRERH--P-G-SF
950115          ----IKVNDTIQIDLETGKITDFIKFDTGNLCMVTGGANLGRIGVITNRERH--P-G-SF
147899358       ----IKVNDTIQIDLETGKITDFIKFDTGNLCMVTGGANLGRIGVITNRERH--P-G-SF
126327259       ----IKVNDTVQIDLEAGKITDFIKFDTGNLCMVTGGANLGRIGVITNREKH--P-G-SF
119901281       ----IKVNDTIQIDLETGKITDFIKFDTGNLCMVTGGANLGRIGVITNRERH--P-G-SF
148229573       ----IKVNDTIQIDLETGKITDFIKFDTGNLCMVTGGANLGRIGVITNRERH--P-G-SF
114689109       ----IKVNDTIQIDLETGKITDFIKFDTGNLCMVTGGANLGRIGVITNRERH--P-G-SF
74136523        ----IKMNDTIQIDLETGKITDFIKFDTGNMCMVTGGANLGRIGVITNREKH--P-G-SF
119934106       ----IKVNDTIQIDLETGKITDFIKFDTGNLCMVTGGANLGRIGVITNRERH--P-G-SF
109083806       ----IKVNDTIQIDLETGKITDFIKFDTGNLCMVTGGANLGRIGVITNRERH--P-G-SF
57113929        ----IKVNDTVQIDLGTGKITSFIKFDTGNVCMVIGGANLGRVGVVTNRERH--P-G-SC
119895079       ----IKVNDTIQIDLETGKITDFIKFDTGNLCMVTGGANLGRIGVITNRERH--P-G-SF
29126987        ----IKVNDTVQISLDSGKITDAIKFDTGNLCMVTGGANLGRIGVITNRERH--P-G-SF
157819705       ----IKVNDTVQISLDSGKITDAIKFDTGNLCMVTGGANLGRIGIITNRERH--P-G-SF
53933236        ----IKVNDTIRIDLDTGKITDFIKFETGNMCMVTGGANLGRIGVITNREKH--P-G-SF
50403757        ----IKVNDTVRIDLGSGKITSFIKFDTGNVCMVIGGANLGRVGVITNRERH--P-G-SF
72533642        ----IKVNDTVQIDLGTGKIINFIKFDTGNLCMVIGGANLGRVGVITNRERH--P-G-SF
124300793       ----IKVNDTVRIDLETGKITEFIKFDTGNLCMVTGGANLGRIGVITNRERH--P-G-SF
4506727         ----IKVNDTVQIDLGTGKIINFIKFDTGNLCMVIGGANLGRVGVITNRERH--P-G-SF
50401280        ----IKANDTVRIDLETGKITEFIKFDTGNLCMVTGGANLGRIGVITNRERH--P-G-SF
133777261       ----IKVNDTVQIDLGTGKIINFIKFDTGNLCMVIGGANLGRVGVITNRERH--P-G-SF
119930339       ----IKVNDTIQIDLETGKIIDFIKFDTGNLCMVTGGANLGRIGVISNRERH--P-G-SF
119913177       ----IKVNDIIQIDLETSKITDFIKFDTGNLCMVTGGANLGRIGVITNRERH--P-G-SF
38503307        ----IKVNDTVQIDLGTGKIINFIKFDTGNVCMVIGGANLGRVGVITNRERH--P-G-SF
38503308        ----IKVNDTVQIDLGTGKIINFIKFDTGNLCMVIGGANLGRVGVITNRERH--P-G-SF
119930307       ----IKVNGTIQIDLETGKITDFIKFDTGNLCMVTGGASLGRIGVITNRERH--P-G-SF
62896747        ----IKVNDTVQIDLGTGKIINFIKFDTGNLCMVIGGANLGRVGVITNRERH--P-G-SF
88703062        ----IKVNDTVQIDLGTGKITSFIKFDTGNVCMVIAGANLGRVGVITNRERH--P-G-SC
57113861        ----IKVNDTVQIDLGTGKIINFIKFDTGNLCMVIGGANLGRVGVITNRERH--P-G-SF
148680472       ----IKVNDTIQIDLETGKVTDFIKFDTGNLCMVTGGANLGRIGVITNRERH--P-G-SF
124486640       ----IKVNDTIQIDLETGKVTDFIKFDTGNLCMVTGGANLGRIGVITNRERH--P-G-SF
28204689        ----IKVNDTVQIDLGTGKIINFIKFDTGNVCMVIGGANLGRVGVITNRERH--P-G-SF
28204681        ----IKVNDTVQIDLGTGKIINFIKFDTGNLCMVIGGANLGRVGVITNRERH--P-G-SF
167045794       ----IKVNDTIQIDLETGKITDFIRFDTGNLCMVTRGANLGRIGVITNRERH--P-G-SF
119902798       ----IKVNDTIQIDLETGKIIDFIKFDTGNLCMVTGGANLGRIGVISNRERH--P-G-SF
28204665        ----IKVNDTVQIDLGTGKIINFIKFDTGNLCMVIGGANLGRVGVITNRERH--P-G-SF
76660997        ----IKVNDTIQIDLETGKITDFMKFDTGNLCMVTGGASLGRIGVITSRERH--P-G-SF
149712494       ----IKVNDTIRIDLETGKITDFIKFDAGNVCMVTGGANLGRIGVITNRERY--P-G-SF
119923668       ----IKVNDTIQIDLETGKIIDFIKFDTGNLCMVTGGANLGRIGVISNRERH--L-G-SF
90820002        ----IKVNDTIQLDIASSKITDYIRFDSGNLCMITGGRNLGRVGTVVNRERH--P-G-SF
119923854       ----IKVNDTIQTDLETGKITDFIKFDTGNLCKVTGGANLGRIGVISNRERH--P-G-SF
70909479        ----VKVNDTIQVEIATSKILDFIKFDSGNLCMITGGRNLGRVGTVVNRERH--P-G-SS
109067331       ----IKVNDTIQIDLETTKITDFIKFDTGNLCMVTGGANLGRIGVITNRERH--P-G-SF
125979105       ----IHANDTVQVDIATGKITDYIKFDSGNLCMITGGRNLGRVGTVVNRERH--P-G-SF
24663668        ----IHANDSVQVDIASGKITDYIKFDSGNLCMITGGRNLGRVGTVVNRERH--P-G-SF
146285351       ----AKVNDTIQLDIATNKIIDIIRMDSGNLCMITGGRNLGRVGTVVNRERH--P-G-SF
148691968       ----IKVNVTIQIDLETGKVTDFIKFDTGNLCMVTGGANLGRIGVITNRERH--P-G-SF
170285559       ----IKVNDTIQLEIATSKILDSIRFEPGNLCMITGGRNLGRVGTVVNRERH--P-G-SF
112984078       ----IKVNDSIQLDIATTKIMDFIKFESGNLCMITGGRNLGRVGTIVSRERH--P-G-SF
91083095        ----IKVNDTIQLEIATSKILDFIKFESGNLCMITGGRNLGRVGTVVSRERH--P-G-SF
75029893        ----IKVNDTVKVDLATGKIDDYIKFDSGNVCMVTGGHNLGRVGVITSRERH--P-G-SF
74844658        ----IKVNDSVQLDIATSKIMDFIKFESGNLCMITGGRNLGRVGTIVSRERH--P-G-SF
66517407        ----IKVNDTIHLDIATGKILDSIRFDSGNLCMITGGRNLGRVGTVVSRERH--P-G-SF
70909489        ----IKVNDTIQLEIATSKIMDSIKFESGNLCMITGGRNLGRVGTVVNRERH--P-G-SF
70909487        ----IKVNDTIQLEIATSKILDFIKFDSGNLCMITGGRNLGRVGTVVSRERH--P-G-SV
156542863       ----IKVNDTIQLEIATGKILDSIRFDTGNLCMITGGRNLGRVGTVVNRERH--Q-G-SF
70909485        ----IKVNDTIQLEIATSKFLDFIKFESNNLCMITGGRNLGRVGTVVNRERH--P-G-SF
440853          ----IHANDSVQVDIASGKITDYIKFDSGNLCMITGGRNLGRVGTVVNRERH--P-G-SF
74007700        ----IKVNDTIQIDLETGKITDFIKFDTGNLCMVTGGANLGRIGVITNRERH--P-G-SF
74829226        ----IKVNDTIQLEIATNKILDSIKFDSGNLCMITGGRNLGRVGTVVSRERH--P-G-SF
22138108        ----IRANDTIKLDLEENKIVDFIKFDVGNVVMVTGGRNRGRVGVIKNREKH--K-G-SF
125593989       ----IKANDTIKIDLETNKIVDFIKFDVGNIVMVTGGRNTGRVGVIKSREKH--K-G-SF
17979233        ----IKPNDTIKLDLEANKIVEFIKFDVGNVVMVTGGRNRGRVGVIKNREKH--K-G-SF
118573852       ----IKVNDTIQLEIANSKIMDSIKFDSGNLCMITGGRNLGRVGTVVNRERH--P-G-SF
18415395        ----IKPNDTIKLDLEENKIVEFIKFDVGNVVMVTGGRNRGRVGVIKNREKH--K-G-SF
1173256         ----IKANDTIKLDLESNKIVDFIKFDVGNVVMVTGGRNRGRVGVIKNREKH--K-G-SF
118480993       ----IKANDTIKLDLESNKIVDFIKFGVGNVVMVTGGRNRGRVGVIKNREKH--K-G-SF
118484853       ----IKANDTIKLDLENNKIVDFIKFDVGNVVMVTGGRNRGRVGVIKNREKH--K-G-TF
```

```
15237195          ----IKPNDTIKLDLEANKIVEFIKFDVGNVVMVTGGRNRGRVGVIKNREKH--K-G-SF
18398393          ----IKPNDTIKLDLEENKIVEFIKFDVGNVVMVTGGRNRGRVGVIKNREKH--K-G-SF
116871421         ----IQVNDSVQVDITTGKITDSIKFDTGNVCMVTGGRNLGRVGLVTNRERH--P-G-SF
73759787          ----IKANDTIKLDLEANKIADFIKFDVGNVVMVTGGRNTGRVGVIKNREKH--K-G-SF
50550587          ----IKVNDTVKIDLATGKITSFVKFENGNIVMTTGGRNMGRVGTITHRERH--E-G-GF
45187482          ----IKVNDTVKVDLATGKITDFIKFDTGKLVYVTGGRNLGRIGVITHRERH--E-G-GF
109067333         ----IKVNDTIQIDLETTKITDFIKFDTGNLCMVTGGANLGRIGVITNRERH--P-G-SF
9759565           ----IKPNDTIKLDLEENKIVEFIKFDVGNVVMVTGGRNRGRVGVIKNREKH--K-G-SF
48376549          ----IKVNDTIQIDLETGKITDFIKFDTGNLCMVTGGANLGRIGVITNRERH--P-G-SF
149287204         ----IKVNDTVKVDQATGKIDDYIKFDSGNVCMVTGGHNLGRVGTIMSRERH--P-G-SF
82400118          ----IKANDTIKLDLESNKIVDFIKFDVGNVVMVTGGRNRGRVGVIKNREKH--K-G-SF
337930            ----IKVNDTIQIDLETGKITDFIKFDTGNLCMVTGGANLGRIGVITNRERH--P-G-SF
159145754         ----VKMNDTVQVDLSTGKIKDFIKFDSGNLCLITGGRNLGRVGVVTHREKH--P-G-SF
1173257           ----IKANDTIKLDLESNKIVDFIKFDVGNVVMVTGGRNRGRVGVIKNREKH--K-G-SF
19112469          ----IKVNDTIKLNLETNKIESFIKFDTSAQVMVTGGRNMGRVGTIVHREHH--L-G-SF
148695326         ----IKVNDTIQIDLETGKITDFIKFDTGNLCMVTGGANLGRIGVITNRERH--P-G-SF
50303511          ----IKVNDTVKVDLATGTITDFIKFDTGKLVYVTGGRNLGRVGTIVHRERH--E-G-GF
71000467          ----IKVNDTVKVDIATGKITDFVRFDTGVVCMVTGGRNMGRVGIITHRERH--D-G-GF
19113142          ----IKVNDTIKLNLETNKIESFIKFDTSAQVMVTGGRNMGRVGTIVHREHH--L-G-SF
19115086          ----IKVNDTIKLNLETNKIESFIKFDTSAQVMVTGGRNMGRVGTIVHREHH--L-G-SF
168027041         ----IKANDTVKINLETGKVVEFIKFDVGNIVMVTGGRNRGRIGVIHHREKH--K-G-SF
146413813         ----IRANDTVKIDLESGKISDFIKFDTGALVMVTGGRNLGRVGVITHREKH--E-G-GF
164429760         ----IKVNDTVKINLETGKIEDFVKFDTGAIAMVTGGRNMGRVGVITHRERH--D-G-GF
6321997           ----IKVNDTVKIDLASGKITDFIKFDAGKLVYVTGGRNLGRIGTIVHKERH--D-G-GF
3914899           ----IKANDTIKIDLETNKIVDFIKFDVGNVVMVTGGRNTGRVGVIKNREKH--K-G-SF
156362157         ----IKVNDTVVIDIKTGKVIDYIKFDTGNMAMVVGGRNMGRVGMVTHREKH--A-G-SF
119493023         ----IKVNDTVKVDIATGKITDFVRFDTGVVCMVTGGRNMGRVGVITHRERH--D-G-GF
126139561         ----IRANDTVKIDLATGKITDFIKFDTGRLVMVTGGRNLGRVGVIVHREKH--E-G-GF
145610060         ----IKVNDTVKINLDTGKITDFIKFDTGALAMVTAGNNMGRVGVITHRERH--D-G-GF
154273927         ----IKVNDTVKIDLATGKITDFIKFDTGAIAMATGGRNMGRVGVITHRERH--D-G-GF
126273970         ----IRANDTVKIDLATGKITDFIKFDTGRLVMVTGGRNLGRVGVIVHREKH--E-G-GF
22758868          ----AKVNDTIMVDIATGKMKEFIKFDSGNLCMITGGHNLGRVGVIQHRERH--P-G-SF
3724352           ----IKVNDTIKLNLETNKIESFIKFDTSAQVMVTGGRNMGRVGTIVHREHH--L-G-SF
119192028         ----IKVNDTVKIDIATGKITDFIKFDTGVICMATGGRNMGRVGVITHRERH--E-G-GF
44894458          ----IKANDTIKIDLETNKIVDFIKFDVGNVVMVTGGRNTGRVGVIKNREKH--K-G-SL
68477663          ----IRANDTVKIDLATGKIDDFIKFDTGRLVMVTGGRNLGRVGVIVHREKH--E-G-GF
145245281         ----IKVNDTVKIDIATGKIADFVKFDTGVVAMATGGRNMGRVGVVTHRERH--D-G-GF
116193489         ----IKVNDTVKIDLETGKATDFIKFDTGAIAMVTGGRNMGRVGVITHRERH--D-G-GF
125552065         ----IKANDTIKIDLETNKIMDFIKFDVGNVVMVTGGRNTGRVGVIKSREKH--K-G-SF
115463485         ----IKANDTIKIDLETNKIVDFIKFDVGNIVMVTGGRNTGRVGVIKSREKH--K-G-SF
158187698         ----VKVNDTIQVDIASGKIKEFIKFDSGNVCMITGGRNLGRVGTITHRERH--P-G-SF
48596901          ----IKANDTIKLDLETNKIVDFIKFDVGNVVMVTGGRNTGRVGVIKNREKH--K-G-TF
2345154           ----IKANDTIKIDLETNKIMDFIKFDVGNVVMVTGGRNTGRVGVIKNREKH--K-G-GF
157690730         ----IKSNDCIKVDIATNKITDYIKFESGNVAMVTGGRNLGRVGIIMHRGRH--P-G-SF
168014621         ----IKANDTVKINLETGKVVEFIKFDIGNIVMVTGGRNRGRIGVIQHREKH--K-G-SF
94468404          ----IHQNDSIQYDIATGKVMDVLKFEPGNLCMITGGRNLGRCGTVIQREKH--P-G-SF
108861826         ----IKPNDTIQVDIATGKVLNHIKFEPGNMCMVTGGRNLGRVGTITQWERH--P-G-SV
115436514         ----IKANDTIKIDLETNKIVDFIKFDVGNVVMVTGGRNTGRVGVIKNREKH--K-G-SF
44967243          ----IKVNDTVQIDLNTGKITDFIKFDTGNQCMVIGGANLGRVGVITNREKH--P-G-SF
115443689         ----IKANDTIKIDLETNKIVDFIKFDVGNVVMVTGGRNTGRVGVIKNREKH--K-G-SF
109509108         ----IHQNDSIQYDIATGKVMDVLKFEPGNLCMITGGRNLGRCGTVIQREKH--P-G-SF
168014095         ----IKANDTVKINLETGKVVEFIKFDIGNIVMVTGGRNRGRIGVIQHREKH--K-G-SF
121704880         ----IKVNDTVKVDIATGKITEFVRFDTGVVVMVTGGRNMGRVGVITHRERH--D-G-GF
168057168         ----IKANDTVKINLETGKVVEFIKFDIGNIVMVTGGRNRGRIGVIQHREKH--K-G-SF
50287861          ----IKVNDTVKVDLASGKITDYIKFDIGKLVYITGGRNLGRIGTIVHKERH--D-G-GF
167998342         ----IKANDTVKINLETGKVVEFIKFDIGNIVMVTGGRNRGRIGVIQHREKH--K-G-SF
61654708          ----INVNDTVRIDLSTGKITDSIKFDIGNLVMVTGGRNLGRVGIITNRERH--H-G-SF
29841450          ----IKPNDTIQVDIATGKVLNHIKFEPGNMCMVTGGRNLGRVGTITQWERH--P-G-SV
170942476         ----IKVNDTVKINLETGKIEDFIKFDTGAIAMVTGGRNMGRVGVITHRERH--D-G-GF
154290538         ----IKVNDTVKIDLATGKITDFIKFDTGVIAMATGGRNMGRVGVITHRERH--D-G-GF
168066840         ----IKANDTVKINLETGKVVEFIKFDIGNIVMVTGGRNRGRIGVIHHREKH--K-G-SF
170034084         ----IHQNDSIQYDIATGKVMDVLKFEPGNLCMITGGRNLGRCGTVIQREKH--P-G-SF
170574388         ----VKVNDTIVVDIATGKMSDYVKFDQGNLCMVTGGRNMGRVGIVGHREKH--P-G-SF
169595016         ----IKVNDTVKIDLSTGKVSDFIKFDTGVIVMVTGGRNMGRVGVITHRERH--D-G-GF
158286707         ----IHQHDSIQYDIATGKVLDVLKFEPGNLCMITGGRNLGRCGTVILREKH--P-G-SF
44967294          ----IKVNDTVQINLESGKITDMIKFDTGNLCMVTGGANLGRIGVITNRERH--P-G-SF
156035911         ----IKVNDTVKIDIATGKITDFIKFDTGVIAMATGGRNMGRVGVITHRERH--D-G-GF
116779177         ----VKANDTIKIDLETGKIKEFIKFDVGNVVMVTGGRNRGRVGVIKHREKH--K-G-SF
119632119         ----IKVNDTVQIDLGTGKITSFIKFDTGNVCMVIAGANLGRVGVITNRERH--P-G-SC
170086746         ----IQVNDTVKFDLEQGKITDYVKFDTGNIVMITGGRNMGRAGVIVHREKH--I-G-GF
116781974         ----VKANDTIKVDLETGKIKEFIKFDVGNVIMVTGGRNRGRVGVIKHREKH--K-G-SF
149239787         ----IKVNDSVKIDLATGKITDFLKFDTGRLVMVTGGRNMGRIGTIVHREKH--E-G-GF
28630198          ----IKVNDTVQIDLDTGKITDFIKFDTGNLCMVIGGANLGRIGVITNRERH--P-G-SF
50426545          ----IRANDSVKVDLATGKVTDFISFDTGRLVMVTGGRNMGRVGVITHREKH--E-G-GF
50423849          ----IRANDSVKVDLATGKITDFISFDTGRLVMVTGGRNMGRVGVITHREKH--E-G-GF
167526949         ----IKVNDTVKVDLDSGRITDFIKFDIGVLVLCTGGRNMGRVGTLMSRDKM--M-G-AT
159466042         ----IKANDSIMLDIETGKIKEFVKNDVGALVMVTGGHNAGRVGKIVHKEKH--K-G-SF
82907488          ----IKVNDNVQISLDSGKITDAIKFDTGNLGMVTGGANLGRTSVITNRERH--P-D-SF
169849885         ----IKVNDTVKFDLEQNKITDFVKFDTGNLVIVTGGRNMGRAGTIVHKEKH--V-G-GF
71015994          ----IKVNDTVRFDIDSQKILDFVAFDTGATVMVTGGRNQGRAGVITGKERH--L-G-GF
119592222         ----IKVNDTIQIDLETGKITDFIKFDTGNLCMVTGGANLGRIGVITNRERH--P-G-SF
145286314         ----IKVNDTVVIDLSSNKITDYVKFEAGNLAMITGGRNVGRVGVIGHREKL--P-G-AF
149635430         ----IKVNDTVQIDLETGKITDFIKFDTGNLCMVTGGANLGRIGVITNRERH--P-G-SF
```

```
95007264        ----IKAHDCIRLDLNTGKIVDTLKFEAGNMAMVTGGHNVGRVGVIVHRERH--L-G-GF
1350992         ----IRANDTVKIDLATGKIXSFIKFDTGRLVMVTGGRNLGRVGVIVHREKL--E-G-GF
47208976        ----IKVNDTVRIDLESGKITDFIKFDT--------GANLGRIGVITNRERH--P-G-SF
58259587        ----IKVNDTVKFDFVQNKIVDHIKFEPGNVVMVTGGRNMGRSGVIVHKERH--L-G-GF
28630195        ----IKENDTVQVNLENGKITDFIKFETGNLCMVTGGANLGRVGIITSRERH--P-G-SF
157337052       ----IKANDTIKLDLESNKITDFIKFDVGNVVMVTGGRNRGRVGVIKNREKR--K-G-SF
66823117        ----ISIHDTIKIDIESGKITAFIPFEVNNLCMIVGGHNLGRVGAVTHREKH--P-G-SF
17367482        ----IKANDTIKLDLETNKITDFIKFDVGNVVMVTGGRNRGRVGVIKNREKH--K-G-SF
17543386        ----VKVNDTIVFNISTQKITDSVKFEPGNLAYVTGGRNVGRVGIIGHRERL--P-G-AS
157756381       ----VKVNDTIVFNINTQKITDFVKFEPGNLAYVTGGRNVGRIGIIGHRERL--P-G-AS
28630193        ----IQVNDSVQVDITTGKITDSIKFDTGNVCMVTGGRNLGRVGLVTNRERH--P-G-SL
79327186        ----IKPNDTIKLDLEENKIVEFIKFDVGNVVMVTGGRNRGRVGVIKNREKH--K-G-SF
157357316       ----IKANDTVKLDLESNKIVDFIKFDVGNVVMVTGGRNRGRVGVIKSREKH--K-G-TF
10177019        ----IKPNDTIKLDLEANKIVEFIKFDVGNVVMVTGGRNRGRVGVIKNREKH--K-G-SF
145355247       ----IKEGDSVMVDIATNKITDFVKFDVGALCMITGGRNAGRVGVIQRREKH--I-G-SF
6598334         ----IKPNDTIKLDLEENKIVEFIKFDVGNVVMVTGGRNRGRVGVIKNREKH--K-G-SF
58257447        ----IKVNDTVKINLDTGKITDFIKFDTGALAMVTAGNNMGRVGVITHRERH--D-G-GF
71029346        ----VKAGDSLRVEVSTGKVLEFLKFEPGNLVMITGGHNVGRVGTVVSKEKH--P-G-SF
146418285       ----IRANDTVKIDLESGKISDFIKFDTGALVMVTGGRNLGRVGVITHREKH--E-G-GF
46128673        ----IKVNDTVKIDLATGKITDFIKFDTGAVVMVTGGRNMGRVGVITHRERH--D-G-GF
37779112        ----IKVNDTVRIDLDTGKITDFIKFDTANLCMVTGGANLGRIGVITNRERH--P-G-SF
67537248        ----IKVNDTVKVDIATGKITDFCRFDTGVVCMVTGGRNMGRVGVVTHRERH--D-G-GF
47777385        ----IKANDTIKIDLETNKIVDFIKFDVGNIVMVTGGRNTGRVGVIKSREKH--K-G-SF
68475117        ----IRANDTVKIDLATGKIDDFIKFDTGRLVMVTGGRNLGRVGVIVHREKH--E-G-GF
164656677       ----VRANDTVKFDLINNKMVDFIKFDTGAQVMITGGRNVGRAGQMIHRERH--H-G-GF
115402491       ----IRVNDTVKVDLATGKITDFVRFDTGVVCMVTGGRNMGRVGVVTHRERH--D-G-GF
156088951       ----VKPGDSLRIDLETGKVLEFFKFEVGNLVMITGGHNQGRVGTIVHKERH--P-G-SF
57047769        ----IKVNDTIQIDLETGKIMDFIKFDTGNLCMVTGGANLGRIGVITNQERY--P-G-SF
149244128       ----IKVNDSVKIDLATGKITDFLKFDTGRLVMVTGGRNMGRIGTIVHREKH--E-G-GF
145528524       ----IHVNDTLKIDLETGKIVDFIKEEPGNICYIIGGNNIGRVGLIQHRERH--L-G-SF
84997205        ----VKAGDSLRVEVSTGKVLEFLKFEPGNLVMITGGHNVGRVGTVVSKEKH--P-G-SF
126654348       ----VKANDTVKVDLATGKVIDFVKCEVGNMCMVTGGRSQGRVGTITHFERK--M-G-AQ
124803509       ----VKVNDTVRLDLETGKVLEHLKFQVGSLVMVTAGHSVGRVGVISSIDKN--M-G-TY
167389537       ----LQANDTIKLNLETGKIVDFVKFDIGNTAMMIGGNGMGRVGVIVKREVH--P-G-SF
55982009        ----IHQNDSIQYDIATGKVMDVLKFEPGNPCMITGGRNLGRCGTVIQREKH--P-G-SF
70946979        ----VKVNDTIRLDLESGKVLEHLKFQVGNMVMVTAGHSVGRVGTILSIDKN--I-G-TY
57107829        ----IKVNDTIQIDLDTGKITDFIKFDTGNLCMVTGGANLGRIGVITNRERH--P-G-SF
159114046       ----IKTNDTIKINLKTGKIDEWYKFDIGKIVMVTGGRNCGRIGTIQAIDKH--M-G-SY
156098302       ----VKVNDTVRLDLETGKVLEHLKFQIGSMVMVTAGHSVGRVGIISSIDKN--I-G-TY
67465453        ----LQANDTIKLNLETGKIVDFVKFDIGNTAMMIGGNGMGRVGVIVKREVH--P-G-SF
167391306       ----LQANDTIKLNLETGKIVDFVKFDIGNTAMMIGGNGMGRVGVIVKREVH--P-G-SF
68073433        ----VKVNDTIRLDLETGKVLEHLKFQVGNMVMVTAGHSVGRVGTILSIDKN--I-G-TY
2463335         ----TRPTSGKRHNQDRSGDQQELRLHQGLMLAMCHGDWRRNTAVWCDQQQGEAK-G-SF
73954159        ----IKMNDTIQIDLETGKITDFFKFDTGNLCMVTGGTNLGRIGVITNRENH--P-G-SF
145475847       ----IHVNDTLKIDLESGKVVEFLKEEPGNLCYIIGGNNIGRVGLIQHRERH--L-G-SF
115692154       ----FSTNSSAR--IHKGGLS----INQGHLCMITGGRNLGRVGTITHREKH--P-G-SF
145488765       ----IHVNDTLKIDLESGKVVEFLKEEPGNLCYIIGGNNIGRVGLIQHRERH--L-G-SF
118573853       ----IKIGDTLKYDLVNNKIENFAHLESGNVCYIQQGNNIGRVGIIQHIEKH--Q-G-SF
160550143       ----VKVNDTIQVNTQTGKIMDFIKFEGGNMCMITGGHNIGRVGQIVHVEKH--P-G-SF
146080976       ----TSIGDTIVYNVKEKKCVDLIKNRQGKAVIVTGGANRGRIGEIVKVECH--P-G-AF
145499397       ----IHVNDTLKIDLETGKVVDFLKEEPGNLCYIIGGNNIGRVGLIQHRERH--L-G-SF
82539137        ----VKVNDTIRLDLESGKVLEHLKFQVGNMVMVTAGHSVGRVGTILSIDKN--I-G-TY
56199522        ----IKINDTVQLDIATGKITDTIKFESGNLCMITGGRNLGRVGTVMNREKH--P-G-SF
118376768       ----IKIGDTLKYDLVNNKIENFAHLESGNVCYIQQGNNIGRVGIIQHIEKH--Q-G-SF
154333974       ----CSIGDTIVYNVNEKKCVDLIKNRQGKAVIVTGGANRGRIGEIVKVERH--P-G-AF
160331849       ----IKTNDTILFDLSEKKIIDFIKFEVGSLCIITGGPNVGRIGIVLQQGKN--F-L-EE
169802405       ----LQANDTIKLNLETGKIVDFVKFDIGNTAMMIGGNGMGRVGVIVKREVH--P-G-SF
167379757       ----LQANDTIKLNLETGKIVDFVKFDIGNTAMMIGGNGMGRVGVIVKREVH--P-G-SF
109124282       ----IKVNDTIQIDLETGKITA-LKFDTGNLCLVTGGGNWGRIGVITNRERH--P-V-SF
2500489         ----IKVNDTIQIDLETGKITDFIKFDTGNLCMVTGGANLGRIGVITNRERH--P-G-SF
157866320       ----TSIGDTIVYNVKEKKCVDLIKNRQGKAVIVTGGANRGRIGEIVKVECH--P-G-AF
146080974       ----TSIGDTIVYNVKEKKCVDLIKNRQGKAVIVTGGANRGRIGEIVKVECH--P-G-AF
123477532       ----VRVHDALKFNIKTGEVESVIKFKVGDVAMVTAGGNVGRVGTIQKIEKQ--M-A-SF
4432939         ----IKVNDTIQIDLETGKITDFIKFDTGNLCMVTGGANLGRIGVITNRERH--P-G-SF
71755051        ----TSRGDTLVYDVKEKKVLDLIKIGNGKVVMVTGGANRGRIGEIVSIERH--P-G-AF
123376214       ----VRVHDALKFNIKTGEVESVIKFKVGDVAMVTAGGNVGRVGTIQKIEKQ--M-A-SF
145495133       ----IHINDTLKIDLETGKVVDFIKEEPGNICYIVGGNNIGRVGLIQHRERH--L-G-SF
145500014       ----IHVNDTLKIELETGKIVDFLKEEPGNLCYIIGGNNIGRVGLIQHRERH--L-G-SF
71415246        ----THRGDTLVYNLKEKKVVDLIKSSNGKVVMVTGGANRGRIGEIMSIERH--P-G-AF
123439290       ----VRVHDALKFNIKTGEVESVIKFKVGDVAMVTAGGNVGRVGTIQKIEKQ--M-A-SF
162606302       ----IKSNDSIVLDLLNNKVIDFIKFSIGSLCVVIKGSNIGRIGVFINTIGG--Q-G-SD
67465619        ----LQANDTIKLNLETGKIVDFVKFDIGNTAMMIGGNGMGRVGVIVKREVH--P-G-SF
167393127       ----LQANDTIKLNLETGKIVDFVKFDIGNTAMMIGGNGMGRVGVIVKREVH--P-G-SF
148700270       ----IKVNDTIQIDLETGKITDFIKFDTGN------------------------------
70909483        ----IKVNDTIQLEIATSKILDFIKFESGNLCMITGGRN---------------------
119623175       ----IKVNDTVQIDLGTGKIINFIKFDTG-------------------------------
57641464        ----YKTSYTVIMQVPERQIVKVLPFEVGAYVFVTQGKNVARKGKIVEVRQF--PMG-WP
14591522        ----F-TSYTVLMKVPEREIMEVLPFEKGAYVFVTQGKNVARKGRIVEIKKF--PMG-WP
124028167       EAKPYKTLGTVKISIPEQQLLGYAPLEVGSLVIIFGGRNVGRVGRIVSIQPG--M-R-RR
15678046        ----FRVGDVVKLSIPEQEILERIPFEKDSLGLVTGGRHTGEIGKIKKINIT--R-S-SM
14520545        ----F-TSYTVLMKVPEREILEVLPFEKGAYVFVTQGKNVARKGRIVEIKKF--PMG-WP
```

```
                    |210  |    |220 |     |230   |     |240  |     |250 |
RPS4X           DVVHVKDA-NGNSFATRLSNIFVIG-KG-N-KPW-ISLPR-GKGIR-LTIAEERDKRLAA
RPS4Y1          DVVHVKDA-NGNSFATRLSNIFVIG-NG-N-KPW-ISLPR-GKGIR-LTVAEERDKRLAT
RPS4Y2          DVVHVKDA-NGNSFATRISNIFVIG-NG-N-KPW-ISLPR-GKGIR-LTIAEERDKRLAA
119592221       DVVHVKDA-NGNSFATRLSNIFVIG-KG-N-KPW-ISLPR-GKGIR-LTIAEERDKRLAA
4506725         DVVHVKDA-NGNSFATRLSNIFVIG-KG-N-KPW-ISLPR-GKGIR-LTIAEERDKRLAA
46048780        DVVHVKDA-NGNSFATRLSNIFVIG-KG-N-KPW-ISLPR-GKGIR-LTIAEERDKRLAA
12851918        DVVHVKDA-NGNSFATRLSNIFVIG-KG-N-KPW-ISLPR-GKGIR-LTIAEERDKRLAA
1350996         DVVHVKDA-NGNSFATRLSNIFVIG-KG-N-KPW-ISLPR-GKGIR-LTIAEERDKRLAA
57090063        DVVHVKDA-NGNSFATRLSNIFVIG-KG-N-KPW-ISLPR-GKGIR-LTIAEERDKRLAA
74179765        DVVHVKDA-NGNSFATRLSNIFVIG-KG-N-KPW-ISLPR-GKGIR-LTIAEERDKRLAA
62896517        DVVHVKDA-NGNSFATRLSNIFVIG-KG-N-KPW-ISLPR-GKGIR-LTIAEERDKRLAA
45360467        DVVHVKDA-NGNSFATRLSNIFVIG-KG-N-KPW-ISLPR-GKGIR-LTIAEERDKRLAA
126722859       DVVHVKDA-NGNSFATRLSNIFVIG-KG-N-KPW-ISLPR-GKGIR-LTIAEERDKRLAA
74136531        DVVHVKDA-NGNSFATRLSNIFVIG-KG-N-KPW-ISLPR-GKGIR-LTIAEERDKRLAA
227229          DVVHVKDR-NGNSFATRLSNIFVIG-KG-N-KPW-ISLPR-GKGIR-LTIAEERDKRLAA
950115          DVVHVKDA-NGNSFATRLSNIFVIG-KG-N-KPW-ISLPR-GKGIR-LTIAEERDKRLAA
147899358       DVVHVKDA-NGNNFATRLSNIFVIG-KG-N-KPW-ISLPR-GKGIR-LTIAEERDKRLAA
126327259       DVVHVKDA-NGNSFATRLSNIFVIG-KG-N-KPW-ISLPR-GKGIR-LTIAEERDKRLAA
119901281       DVVHVKDA-NGNSFATRLSNIFVIG-KG-N-KPW-ISLPR-GKGIR-LTIAEERDKRLVA
148229573       DVVHVKDA-NGNNFATRLSNIFVIG-KG-N-KPW-ISLPR-GKGIR-LTIAEERDKRLAA
114689109       DVVHVKDA-NGNSFATRLSNIFVIG-KG-N-KPW-ISLPR-GKGIR-LTIAEERDKRLAA
74136523        DVVHVKDA-NGNSFATRLSNIFVIG-KG-N-KPW-ISLPR-GKGIR-LTIAEERDKRLAA
119934106       DVVHVKDA-NGNSFATRLSNIFVIG-KG-N-KPW-ISLPR-GKGIR-LTIAEERDKRLAA
109083806       DVVHVKDA-NGNSFATQLSNIFVIG-KG-N-KPW-ISLPR-GKGIR-LTIAEERDKRLAA
57113929        DVVHVKDA-NGNSFATRISNIFVIG-NG-N-KPW-ISLPR-GKGIR-LTIAEERDKRLAA
119895079       DVVHVKDA-NGNSFATRLSNIFVTG-KG-N-KPW-ISLPR-GKGIR-LTIAEERDKRLAA
29126987        DVVHVKDA-NGNGFATRLSNIFVIG-KG-N-KPW-ISLPR-GKGIR-LTIAEERDKRLAA
157819705       DVVHVKDA-NGNGFATRLSNIFVIG-KG-N-KPW-ISLPR-GKGIR-LTIAEERDKRLAA
53933236        DVVHVKDS-IGNSFATRLSNIFVIG-KG-N-KPW-VSLPR-GKGVR-LTIAEERDKRLAA
50403757        DVVHVKDA-SGNSFATRISNIFVIG-NG-N-KPW-ISLPR-GKGIR-LTIAEERDKRLAA
72533642        DVVHVKDA-NGNSFATRLSNIFVIG-NG-N-KPW-ISLPR-GKGIR-LTVAEERDKRLAA
124300793       DVVHVKDS-TGNSFATRLSNIFVIG-KG-N-KPW-VSLPR-GKGIR-LTIAEERDKRLAA
4506727         DVVHVKDA-NGNSFATRLSNIFVIG-NG-N-KPW-ISLPR-GKGIR-LTVAEERDKRLAT
50401280        DVVHVKDS-TGNSFATRLSNIFVIG-KG-N-KAW-VSIPR-GKGIR-LTIAEERDKRLAA
133777261       DVVHVKDA-NGNSFATRLSNIFVIG-NG-N-KPW-ISLPR-GKGIR-LTVAEERDKRLAA
119930339       DVVHVKDA-NGNSFATRLSNIFIIG-KG-N-KPW-ISLPR-GKGIR-LTIAEERDKRLAA
119913177       DVVHVKDA-NGNSFATRLSNIFIIG-KG-N-KPW-TSLPC-GKGIC-LTIAEERDKRLAA
38503307        DVVHVKDA-NGNSFATRISNIFVIG-NG-N-KPW-ISLPR-GKGIR-LTIAEERDKRLAT
38503308        DVVHVKDA-NGNSFATRISNIFVIG-NG-N-KPW-ISLPR-GKGIR-LTVAEERDKRLAT
119930307       DVVHVKDA-NGNSFATRLSNIFVFG-KG-N-KPW-ISLPR-GKGIC-LTIAEERDKRLAA
62896747        GVVHVKDA-NGNSFATRLSNIFVIG-NG-N-KPW-ISLPR-GKGIR-LTVAEERDKRLAT
88703062        DVVHVKDA-NGNSFATRISNIFVIG-NG-N-KPW-ISLPR-GKGIR-LTIAEERDKRLAA
57113861        DVVHVKDA-NGNSFATRLSNIFVIG-NG-N-KPW-ISLPR-GKGIR-LTVAAERDKRLAT
148680472       DVVHVKDA-SGNSFATRLSNIFVIG-KG-N-KPW-ISLPR-GKGVR-LTIAEERDKRLAA
124486640       DVVHVKDA-SGNSFATRLSNIFVIG-KG-N-KPW-ISLPR-GKGVR-LTIAEERDKRLAA
28204689        DVVHVKDA-NGNSFATRISNIFVIG-NG-N-KPW-ISLPR-GKGIR-LTIAEERDKRLAT
28204681        DVVHVKDA-NGNSFATRISNIFVIG-NG-N-KPW-ISLPR-GKGIR-LTVAEERDKRLAT
167045794       NVVHVKDA-NGNSFATQLSNIFVIG-KG-N-KPW-ISLPR-GKGIR-LTIAEERDKRLVA
119902798       DVVHVKDA-NGNSFATRLSNIFIIG-KG-N-KPW-ISLPR-GKGIR-LTIAEERDKRLAA
28204665        DVVHVKDA-NGNSFATRLSNIFVIG-NG-N-KPW-ISLPR-GKGIR-LTVAAERDKRLAT
76660997        DVVHVKDA-NGNSFATRLSDIFVIG-KG-N-KPW-ISLPH-GKGIR-LTIAEERDKRLAV
149712494       EVVHVKDA-NGNSFATRLSNIFVIG-KG-N-KPW-ISLPR-GKGIR-LTIAEERDVRLAT
119923668       DVVHVKDA-NGNSFATWLSNIFIIG-KG-N-KPW-ISLPR-GKGIR-LTIAEERDKRLAA
90820002        DIVHIKDT-NGHTFATRLVNVFIIG-KG-T-KPY-VSLPK-GKGVK-LSIAEERDKRLAA
119923854       DVVHVKDA-NGNSFATWLSNIFIIG-KG-N-KPW-ISLPR-GKGIC-LTIAEERDKRLAA
70909479        DIVHIKDA-NGHTFATRLNNVFIIG-KG-N-KAL-VSLPR-GKGVK-LSIAEERDKRLAA
109067331       DVVHVKDA-NGNSFATRLSNIFVID-KG-N-KPW-ISLPQ-GKGIR-LTIAEE-------
125979105       DIVHIKDS-QGHVFATRLTNVFIIG-KG-S-KPY-ISLPK-GKGVK-LSISEERDKRLAA
24663668        DIVHIKDS-QGHVFATRLTNVFIIG-KG-N-KPY-ISLPK-GKGVK-LSIAEERDKRLAA
146285351       DIVHVKDA-TGHTFATRLNNVFIIG-KG-S-KAY-VSIPK-GKGIK-LSIAEERDRRLAA
148691968       DVVHVKDA-NGKSFATRLSNIFVIG-KG-N-KSW-ISLPR-GKGIR-LTIAEERQEACGQ
170285559       DICHIKDS-QGHTFATRLNNVFIIG-KG-A-KPY-VSLPR-GKGIK-LSIAEERDKRLAA
112984078       DIVHIKDS-TGHTFATRLNNVFIIG-KG-T-KAY-ISLPR-GKGIR-LTIAEERDKRIAA
91083095        DIVHIKDA-NGHTFATRLNNVFIIG-KG-S-KPY-VSLPR-GKGVK-LSIAEERDKRLAA
75029893        DIVHVKDS-QGHSFATRLNYIFVIG-KS-T-KPY-ISLPR-GKGIK-LSVAEERDRRLQA
74844658        DIVHIKDS-TGHTFATRLNNVFIIG-KG-T-KAY-ISLPR-GKGVR-LTIAEERDKRIAA
66517407        DICHIKDS-QGHTFATRLNNVFIIG-KG-T-KPY-ISLPR-DKGVK-LSIAEERDKRLAA
70909489        DIVHIKDS-QGHIFATRLNNVFIIG-KG-S-KAY-VSLPR-GKGVK-LSIAEERDKRLAA
70909487        DIVHVKDA-NGHTFATRLNNVFIIG-KG-S-KPY-VSLPR-GKGVK-LSIAEERDKRLAA
156542863       DICHIKDS-QGHTFATRLHNVFIIG-KG-T-KPY-VSLPR-GNGVK-LTIAEERDKRLAA
70909485        DIVHIKDA-NGHVFATRLNNVFIIG-KG-S-KAF-VSLPR-GKGVK-LSIAEERDKRLAA
440853          DIVHIKDS-QGHVFATRLTNVFIIG-KG-N-KPY-ISLPK-GKGVK-LSIAEERDKRLAA
74007700        DVVHVKDA-NGNSFATRLSNIFVIG-KG-N-KPW-ISLPR-GKGIR-LTIAEERDKRLAA
74829226        DICHIKDS-QGHTFATRMNNVFIIG-KG-S-KAY-VSLPR-GKGVK-LSIAEERDKRLAA
22138108        ETIHVQDS-TGHEFATRMGNVFTIG-KG-T-KPW-ISLPK-GKGIK-LSIIEEARKRIAA
125593989       ETIHVEDA-LGHQFATRMGNVFTIG-KE-R-KPW-VSLPK-GKGIK-LSIIEEARKRNAE
17979233        ETIHIQDS-TGHEFATRLGNVYTIG-KG-T-KPW-VSLPK-GKGIK-LTIIEEARKRLAS
118573852       DIVHIKDS-QGHTFANRLNNVFIIG-KG-S-KAY-VSLPR-GKGVK-LSIAEERDKRLAA
18415395        ETIHIQDS-TGHEFATRLGNVYTIG-KG-T-KPW-VSLPK-GKGIK-LTIIEEARKRLAS
1173256         ETIHVQDA-AGHEFATRLGNVFTIG-KG-T-KPW-VSLPK-RKGIK-LSIIEEARKRLAA
118480993       ETIHVQDA-TGHEFATRLGNVFTIG-KG-S-KPW-ISLPK-GKGIK-LSIIEEAKERLAA
118484853       ETIHVQDA-TGHEFATRLGNVFTIG-KG-T-KPW-ISLPK-GKGIK-LSIIEEAKKRLAA
```

15237195	ETIHIQDS-TGHEFATRLGNVYTIG-KG-T-KPW-VSLPK-GKGIK-LTIIEEARKRLAS
18398393	ETIHIQDS-TGHEFATRLGNVYTIG-KG-T-KPW-VSLPK-GKGIK-LTIIEEARKRLSA
116871421	DIVHVKDT-AGHTFATRLADIFIIG-RG-N-KPW-VSLPK-GKGIP-SVHRKERDRRLAL
73759787	ETVHIQDS-LGHEFATRLGNVFTIG-KG-T-KPW-VSLPK-GKGIK-LTIIEEARKRNVA
50550587	DIVHIKDA-LDNQFVTRLTNVFVIG-EG-N-KSL-ISLPK-GKGIK-LSIAEERDARRAK
45187482	DLVHIKDS-LENTFVTRLNNVFVIG-EQ-G-RPW-ISLPR-GKGIK-LSIAEERDRRRAQ
109067333	DVVHVKDA-NGNSFATRLSNIFVID-KG-N-KPW-ISLPQ-GKGIR-LTIAEE-------
9759565	ETIHIQDS-TGHEFATRLGNVYTIG-KG-T-KPW-VSLPK-GKGIK-LTIIEEARKRLAS
48376549	DVVHVKDA-NGNSFATRLSNIFVIG-KG-N-KPW-ISLPR-GKGIR-LTIAEERDKRLAA
149287204	DIVHVKDS-QGHTFATRLSYIFIIG-KG-T-KPY-ISLPR-GKGVK-LSVAEERDRRLQI
82400118	ETLHIQDS-QGHEFATRLGNVFTLG-KG-T-KPW-VSLPK-GKGIK-LTIIEEAKKRLAA
337930	DVVHVKDA-NGNSFATRLSNIFVIG-KG-N-KPW-ISLPR-GKGIR-LTIAEERDKRLAA
159145754	DIVHIKDA-VGHNFATRASNVFIIG-KG-N-KPW-ISLPK-GKGVK-LSIAEERDRRLMQ
1173257	ETLHIQDS-QGHEFATRLGNVFTLG-KG-T-KPW-VSLPK-GKGIK-LTIIEDARKRLAA
19112469	EIIHVKDA-LDREFATRLSNVFVIG-EA-G-KSW-ISLPK-GKGVK-LSITEERDRRRAL
148695326	DVVHVKDA-NGNSFATRLSNIFVIG-KG-N-KPW-ISLPR-GKGIR-LTIAEERDKRLAA
50303511	DLVHIKDS-LENTFVTRLNNVFVIG-EP-G-RPW-ISLPK-GKGIK-LTISEERDRRRAQ
71000467	NIVHIKDA-IDNSFATRESNVFVIG-Q--E-KPW-ISLPK-GKGVK-LTIAEERDRRRA-
19113142	EIIHVKDA-LDREFATRLSNVFVIG-ET-G-KSW-ISLPK-GKGVK-LSITEERDRRRAL
19115086	EIIHVKDA-LDREFATRLSNVFVIG-ET-G-KSW-ISLPK-GKGVK-LSITEERDRRRAL
168027041	DIIHVTDA-TGNQFATRMGNVYTIG-QG-T-KPW-ITLPR-GKGIK-LSIVEEAKKRKAT
146413813	DLVHIKDS-LENTFVTRLTNVFVIGTEA-G-KPW-VSLPK-GKGIK-LSISEERDRRRAQ
164429760	NIIHVKDA-LDNTFATRESNVFVIG--S-E-KPW-ISLPK-GKGVK-LSIAEERDRRRAN
6321997	DLVHIKDS-LDNTFVTRLNNVFVIG-EQ-G-KPY-ISLPK-GKGIK-LSIAEERDRRRAQ
3914899	ETIHVEDS-LGHQFATRMGNVFTIG-KG-N-KPW-VSLPK-GKGIK-LSIIEEQRKR---
156362157	DIVHVKDA-TGHQFATRLTNIFVIG-KG-N-KPY-VSLPK-GKGVR-LSIAEERDRRIAE
119493023	NIVHIKDA-IDNSFATRESNVFVIG-Q--E-KPW-ISLPK-GKGVK-LTIAEERDRRRA-
126139561	DLVHIKDS-LDNTFVTRLANVFVIGTEA-G-KPW-VSLPK-GKGIK-LSISEERDRRRAQ
145610060	NIVHLKDA-IDNTFTTRESNVFVIG--T-E-KPW-ISLPK-GKGVK-LTIAEERDRRRAQ
154273927	NIVHIKDA-IDNSFATRESNVFIIG-Q--D-KPW-ISLPK-GKGVK-LSIAEERDRRRAM
126273970	DLVHIKDS-LDNTFVTRLANVFVIGTEA-G-KPW-VSLPK-GKGIK-LSISEERDRRRTQ
22758868	DIVHIKDA-LGHTFATRLSYVFVIG-KG-N-KPW-ISLPR-GKGVK-LSIAEERDRRMAA
3724352	EIIHVKDA-LDREFATRLSNVFVIG-ET-G-KSW-ISLPK-GKGVK-LSITEERDRRRAL
119192028	GIVHIKDA-IDNTFATRESNVFVIG--H-E-KPW-VSLPK-GKGVK-LSIAEERDRRRAM
44894458	ETIHVEDS-LGHQFATRMGNVFTIG-KG-N-KPW-VSLPK-GKGIK-LSIIEEQRKR---
68477663	DLVHIKDA-LENTFVTRLSNVFVIGTEA-G-KPW-VSLPK-GKGIK-LSISEERDRRRAQ
145245281	NIVHIKDA-IDNTFATRESNVFVIG-Q--D-KPW-ISLPK-GKGVK-LSIAEERDRRRAL
116193489	NIVHLKDA-IDNTFTTRESNVFVIG--S-E-KPW-ISLPK-GKGVK-LTIAEERDRKRAA
125552065	ETIHVEDA-LGHQFATRMGNVFTIG-KE-R-KPW-VSLPK-GKGIK-LSIIEEARKRNAE
115463485	ETIHVEDA-LGHQFATRMGNVFTIG-KE-R-KPW-VSLPK-GKGIK-LSIIEEARKRNAE
158187698	DVVHIKDS-MGHTFATRLNNVFVIG-KG-L-KPW-VSLPG-GRGLK-LTIAEERDRRIAA
48596901	ETIHVEDA-QGHQFATRLGNVFTIG-KG-T-KPW-VTLPK-GKGIK-LTIIEEQRKR---
2345154	DTIHVEDS-MGHQFATRMGNVFTIG-KG-N-KPW-VSLPK-GKGIK-LSIIEEQRRR---
157690730	DIVHVKDA-AGHSFATRLNYIFTIG-KG-S-KPW-VSLPE-GKGIR-MTVAEERDKRIAA
168014621	DIIHVTDS-TGQQFATRMGNVFTVG-QG-T-KPW-ITLPR-GKGIK-LSIVEEAKKRTQA
94468404	EIVHVKDA-TGHVFATRLTNVFIIG-KA-T-KAF-ISLPK-GKGVK-LSIAEERDKRLAS
108861826	EMVHVRDS-TGHQFVTRLNNIFIIG-KS-N-QPW-ISLPK-NRGIR-LSIIEERDKRIRA
115436514	ETIHVEDA-LGHAFATRLGNVFTIG-KG-N-KPW-VSLPK-GKGIK-LSIIEEQRKR---
44967243	DVVHVKDA-NGNSFATRLSNIFVIG-KG-N-KPW-ISLPR-GKGIR-LTI----------
115443689	ETIHVEDA-LGHQFATRLGNVFTIG-KG-N-KPW-VSLPK-GKGIK-LSIIEEQRKR---
109509108	EIVHIKDS-TGHVFATRLTNVFIIG-KG-T-KAF-ISLPK-GKGVK-LSIAEERDKRLAS
168014095	DIIHVTDS-TGQQFATRMGNVFTVG-QG-T-KPW-ITLPR-GKGIK-LSIVEEAKKRTQA
121704880	NIVHIKDA-IDNTFATRESNVFVIG-Q--D-KPW-ISLPK-GKGVK-LSIAEERDRRRA-
168057168	DIIHVTDS-TGNQFATRMGNVFTIG-QG-T-KPW-ITLPR-GKGIK-LSIVEEAKKRKA-
50287861	DLVHVKDS-LDNTFVTRLNNVFVIG-EQ-G-KPY-ISLPK-GKGIK-LTIAEERDRRRAQ
167998342	DIIHVTDA-TGQQFATRMGNVFTVG-QG-T-KPW-ITLPR-GKGIK-LSIVEEAKKRGQA
61654708	DIIHVKDA-AGKSFATRMTNVFVIG-KG-N-KAM-VSIPR-QKGIR-LTIAEERDRRLEA
29841450	EMVHVRDS-TGHQFVTRLNNIFIIG-KS-N-KPW-ISLPK-SRGIR-LSIIEERDKRIRA
170942476	NIVHLKDA-IDNSFATRETNVFVIG-Q--D-KPW-ISLPK-GKGVK-LTIAEERDRKRA-
154290538	NIVHIKDA-IDNSFATRESNVFVIG--A-E-KPW-ISLPK-GKGVK-LTIAEERDRRRA-
168066840	DIIHVTDA-IGNQFATRMGNVFTIG-QG-T-KPW-ITLPR-GKGIK-LSIVEEAKKRKAT
170034084	EIVHIKDS-TGHVFATRLTNVFIIG-KG-T-KAF-ISLPK-GKGVK-LSIAEERDKRLAS
170574388	DIVHIKDA-ADHSFATRLNNVFIIG-KG-T-KSI-VSLPGPTKGVR-LTIAEERDRRLAQ
169595016	NIVHLKDA-VDNEFTTRETNVIVIG-K--E-KPW-ISLPK-GKGVK-LSIAEERDRRRA-
158286707	EIVHVKDT-TGHVFATRLSNVFIIG-KS-T-KAY-ISLPK-GKGVK-LTIAEERDRRLAN
44967294	DVVHVKDA-NGNSFATRLSNIFVIG-KG-N-KPW-VSLPR-GKGIR-LTI----------
156035911	NIVHIKDA-IDNSFATRESNVFVIG--A-E-KPW-ISLPK-GKGVK-LTIAEERDRRRA-
116779177	EIIHVQDA-AGQEFATRQGNVFTIG-KG-T-KPW-VSLPK-GKGVK-LSIIEEARKR---
119632119	DVVHVKDA-NGNSFATRISNIFVIG-NG-N-KPW-ISLPR-GKGIR-LTIAEERDKRLAA
170086746	DIVHVKDS-LDRTFATRVTNIFVIG-EG-I-KPW-ISLPK-GKGTK-LTISEERDVKRKQ
116781974	EIIHVQDA-AGQEFATRQGNVFTIG-KG-T-KPW-VSLPK-GKGVK-LSIIEEARKR---
149239787	DLVHIKDA-LERTFVTRLNNVFVIGEEA-G-KPW-VSLPK-GKGIK-LSITEERDR---A
28630198	DVVHVKDA-NGNSFATRLSNIFVIG-KG-N-KPW-VSLPR-GKGIR-LTI----------
50426545	DLVHIKDS-LENTFVTRLTNVFIVGTEA-G-KPH-ISLPK-GKGIK-LSISEERDRRRNQ
50423849	DLVHIKDS-LENTFVTRLTNVFIVGTEA-G-KPH-ISLPK-GKGIK-LSISEERDRRRNQ
167526949	DVVHVRDS-AGQQFTTLLSNVFAVG-SG-K-KPA-ISLPR-GKGVR-LTIAEERDRRLKD
159466042	EIVHIEDA-AGNRFATRLTNIFVIG-KE-G-KPL-ISLPK-GKGIR-LTIIQEQKKRYEA
82907488	DMVHMKDA-NGNGFATRLSNIFMIW-KG-K-KPW-ISLPR-GKGIR-LTIAEERDKRLAA
169849885	DIVHIKDS-LDRTFATRVSNVFVIG-EG-T-KAW-ISLPK-GKGLK-LTIAEERDVRRKR
71015994	DIVHIRDV-LGRDFSTRLSNVFVIG-TD-G-KPM-ISMPR-GGGVK-LTITEERDQRRRQ
119592222	DVVHVKDA-NGNSFATRLSNIFVIG-K---------------------------------
145286314	DIIHVKDA-AGHSFATRVSNVFVIG-KG-N-KAL-VSLPA-AKGIR-LTIAEERDKRLAQ
149635430	DVVHVKDA-NGNSFATRLSNIFVIG-K---------------------------------

95007264 DIIHLRDA-KNNEFATRISNVFVIG-KG-E-KAW-ISLPK-EKGIR-LSIMENRQVLLKK
1350992 DLVHIKDA-LENTFVTRLSNVFVIGTEA-G-KPW-VSLPK-GKGIK-LSISEERDRRXAQ
47208976 DVVHVKDT-TGNSFATRLSNIFVIG-KG-N-KPW-VSLPR-GKGIR-LTIAEERDKRLAA
58259587 DIVHVKDV-LDRTFATRLSNIFVIG-EG-S-KAQ-VSLPK-GKGVK-LSIA---------
28630195 DVVHVKDT-AGNTFATRLGNIFVIG-KA-Q-KAW-VSLPR-GKGIR-LTI----------
157337052 ETIHVQDA-TGHEFATRLGNVFIIG-KG-T-KPW-VTLPK-GKGIK-LSIIEEAQKRLAA
66823117 DIVHVTDT-AGHQFATRLSNVFIIG-KA-S-QTF-VSLPA-GKGVR-RSRVDERNAALK-
17367482 ETIHVQDA-TGHEFATRLGNVFTIG-KG-T-KPW-VSLPK-GKGIK-LTILEEAKKR---
17543386 DIIHIKDS-AGHSFATRISNVFVIG-KG-N-KAL-VSLPT-GAGIR-LSIAEERDKRMAQ
157756381 DIIHIKDA-AGHSFATRISNVFVIG-KG-N-KAL-VSLPS-GNGVR-LSIAEERDKRIAQ
28630193 DIVHVKDT-AGHTFATRLSNIFVIG-RG-N-KPW-VSLLK-GKGIH-LTI----------
79327186 ETIHIQDS-TGHEFATRLGNVYTIG-KG-T-KPW-VSLPK-GKGIK-LTIIEEARKRLAS
157357316 ETIHVQDA-TGHEFATRLGNVFTIG-KG-T-KPW-VTLPK-GKGIK-LSIIEEAKKRLAA
10177019 ETIHIQDS-TGHEFATRLGNVYTIG-KG-T-KPW-VSLPK-GKGIK-LTIIEEARKRLAS
145355247 EIVHLKDA-AGQEFATRATNVFAIG-TG-S-KPM-ISLPK-GKGIK-LSIVDER------
6598334 ETIHIQDS-TGHEFATRLGNVYTIG-KG-T-KPW-VSLPK-GKGIK-LTIIEEARKRLSA
58257447 NIVHLKDA-IDNTFTTRESNVFVIG--T-E-KPW-ISLPK-GKGVK-LTIAEERDRRRAQ
71029346 DLVHVKDS-QDNTFSTRSSNVFVIG-VG-T-KSY-VSLPY-ERGLR-KTIIEQRNERLAK
146418285 DLVHIKDS-LENTFVTRLTNVFVIGTEA-G-KPW-VSLPK-GKGIK-LSISEERDRRRAQ
46128673 NIVHVKDA-IDNSFATRESNVFIIG-Q--D-KPW-ISLPK-GKGVK-LTIAEERDRRRA-
37779112 DVVHVTDS-TGNNFATRLSNI-------------------------------------
67537248 NIVHVKDA-IDNTFATRESNVFVIG-Q--D-KPW-ISLPK-GKGVK-LSIAEERDRRRA-
47777385 ETIHVEDA-LGHQFATRMGNVFTIG-KE-R-KPW-VSLPK-GKGIK-LSIIEEARKRNAE
68475117 DLVHIKDA-LENTFVTRLSNVFVIGTEA-G-KPW-VSLPK-GKGIK-LSISEERDRRRAQ
164656677 DIVHVRDP-AGREFSTRLSNVFVIG-DH-E-KRW-ISMPR-GGGVK-LTITEERDLRLKK
115402491 NIVHVKDA-IDNSFATRESNVFVIG-Q--D-KPW-ISLPK-GKGVK-LSIAEERDRRRA-
156088951 DLIHVRDE-LGNNFCTRCSNVFVIG-VG-T-NNY-VSLPK-ERGIK-KGIIEDRADRLA-
57047769 NVVHVKDA-NGNSFAT---
149244128 DLVHIKDA-LERTFVTRLNNVFVIGEEA-G-KPW-VSLPK-GKGIK-LSITEERDR---A
145528524 DIVHVKDS-NGKHFSTRINNIFTIG-KG-K-KSL-ISLPD-DNGLY-LTALEKKQAR---
84997205 DLVHVKDS-QDSTFSTRSTNVFVIG-VG-T-KSY-VSLPA-ERGLR-KTIIEQRNERLAK
126654348 CIVTIRDQ-KGATFATLMKNIFVIG-EE-G-KPL-VTLPK-DKGIR-LSNVEDRNLRMKK
124803509 DIIHVKDS-RNKVFATRLSNVFVIG-DN-T-KPY-ISLPR-EKGIK-LDIIEERRNRLKA
167389537 EIVHIKDA-KGNTFTTRLNNVFVIG-KG-T-ETL-VNLPL-DKGIK-KPLLQQVNETIKK
55982009 EIVHVKDA-TGHVFATRSE---------------------------------------
70946979 DIIHVKDS-RGKVFATRLSNIFVIG-DA-T-KPY-ISLPR-EKGIK-LDIIEERRNRLKA
57107829 DVVHVKDA-NGNSFAT---
159114046 TMIRMKDT-EGTEFLTRLCNVFVIG-N--D-SPA-VAIPT-TKGIR-PDIIKNRELRLRS
156098302 DIIHVKDS-RGKIFATRLSNVFVIG-DN-S-KPY-ISLPR-EKGIK-LDIIEERRNKLKA
67465453 EIVHIKDA-KGNTFTTRLNNVFVIG-KG-T-ETL-VNLPL-DKGIK-KPLLQQVNETIKK
167391306 EIVHIKDA-KGNTFTTRLNNVFVIG-KG-T-ETL-VNLPL-DKGIK-KPLLQQVNETIKK
68073433 DIIHVKDS-RGKIFATRLSNIFVIG-DA-L-KPY-ISLPR-EKGIK-LDIIEERRNRLKA
2463335 ETIHVEDA-LGHQFATRLGNVFTIG-KG-N-KPW-VSLPK-GKGIK-LSIIE--------
73954159 DVVHVKDA-NGNSFATRLSNIFVIG-KG-N-IPW-ISLPH-GKGIR-FTIAEEKKR----
145475847 DIVHVKDS-NGKHFSTRINNIFTIG-KG-K-KSL-ISLPD-DNGLY-LTALEKKQAK---
115692154 EIVHIKDA-SDHTYATRLGNVFVIG-KA-N-KAY-VSLPK-GKGIR-LTIAEEREKRIAQ
145488765 DIVHVKDS-NGKHFSTRINNIFTIG-KG-K-KSL-ISLPD-DNGLY-LTALEKKQAK---
118573853 DICHVKDA-KGNAFATRLGNIFVLG-QG-K-KLY-IELPS-GDGVR-ETILEERKRK---
160550143 HIGHVKDA---
146080976 NIAHLKDA-SGAEFATRAANIFVIG-KDLN-HLQ-VTVPK-QQGLR-MNVIQEREERLIA
145499397 DIVHVKDS-NGKHFSTRINNIFTIG-KG-K-KSL-ISLPD-DNGLY-LTALEKKQAR---
82539137 DIIHVKDS-RGKVFATRLSNIFVIG-DN-T-KPY-ISLPR-EKGIK-LDIIEERRNRLKA
56199522 EIVHIQ---
118376768 DICHVKDA-KGNAFATRLGNIFVLG-QG-K-KSW-IELPS-GDGVR-ETILEERKRK---
154333974 DIAHLKDA-SGAEFATRAANIFVIG-KDLN-NLQ-VTVPK-QQGLR-MNVIQEREERLIA
160331849 EMIKLKDA-KGSEFSTKFSFIFVIG-KG-K-KSF-ISLPK-GKGIR-------------
169802405 EIVHIKDA-KGNTFTTRLNNVFVIG-KG-T-ETL-VNLPL-DKGIK-KPLLQQVNETIKK
167379757 EIVHIKDA-KGNTFTTRLNNVFVIG-KG-T-ETL-VNLPL-DKGIK-KPLLQQVNETIKK
109124282 DTFHVKDA-NSNSF----SIIFVID-KG-N-KPW-ISLP--------------------
2500489 DVVHVKDA---
157866320 NIAHLKDA-SGAEFATRAANIFVIG-KDLN-NLQ-VTVPK-QQGLR-MNVIQEREERLIA
146080974 NIAHLKDA-SGAEFATRAANIFVIG-KDLN-HLQ-VTVPK-QQGLR-MNVIQEREERLIA
123477532 DIVHLKDT-SGAVFATRIMNVFVIG-EN-E-HPL-ISLPA-REGVR-PSILE--------
4432939 DVVHVKDA---
71755051 DIARLKDA-SGHEFATRATNIFVIG-KD-M-SSVPVTLPK-QQGLR-INVIQEREEKLIA
123376214 DIVHLKDT-SGAVFATRIMNVFVIG-EN-E-HPL-ISLPA-REGVR-PSILE--------
145495133 DIVHVKDS-NGKHFSTRINNIFTIG-KG-K-KPL-ISLPD-DNGLY-LTALEKKQAR---
145500014 DIVHVKDS-NGKHFSTRINNIFTIG-KG-K-KSL-ISLPD-DNGLY-LTALEKKQAR---
71415246 DIARLKDA-AGHEFATRASNIFVIG-KD-M-QSVPVTLPK-QQGLR-INVIQEREEKLIA
123439290 DIVHLKDT-SGAVFATRIMNVFVIG-EN-E-HPL-ISLPA-REGVR-PSILE--------
162606302 SIVCLKDF-NGSSFSTQTKNLFVIG-KG-T-KSF-ISLPR-FRGLRTLTKSLEHD-----
67465619 EIVHIKDA-KGNTFTTRLNNVFVIG-KG-T-ETL-VNLPL-DKGIK-KPLLQQVNETIKK
167393127 EIVHIKDA-KGNTFTTRLNNVFVIG-KG-T-ETL-VNLPL-DKGIK-KPLLQQVNETIKK
148700270 ---
70909483 ---
119623175 ---
57641464 DVVTIEDE-NGELFDTLKEYAFVIG-K--D-KPE-ISLP--------------------
14591522 DVVTIEDE-EGELFDTLKEYAFVVG-K--D-KPK-ISLP--------------------
124028167 GIVTIEDA-RGEKIQTSLEYVFVIA-PP-NEEPW-ISLPE-G-----------------
15678046 PNTAVIETGAGKTFLTLKDYVFVIG-K--D-ESV-ISLPG-GK----------------
14520545 DVVTIEDE-EGELFDTLKEYAFVVG-R--D-KPR-ISLP--------------------

```
                 |260
RPS4X           -KQSSG
RPS4Y1          -KQSSG
RPS4Y2          -KQSSG
119592221       -KQSR-
4506725         -KQSSG
46048780        -KQSSG
12851918        -KQSSG
1350996         -KQSSG
57090063        -KQSSG
74179765        -KQSSG
62896517        -KQSSG
45360467        -KQSSG
126722859       -KQSSG
74136531        -KQSSG
227229          -KQSSG
950115          -KQSSG
147899358       -KQSSG
126327259       -KQSSG
119901281       -KQSSG
148229573       -KQSSG
114689109       -KQSSG
74136523        -KQNNG
119934106       -KQSSG
109083806       -KQSSG
57113929        -KQSSG
119895079       -KQSSG
29126987        -KQS--
157819705       -KQS--
53933236        -KQSS-
50403757        -KQSSG
72533642        -KQSSG
124300793       -KQGS-
4506727         -KQSSG
50401280        -KQSS-
133777261       -KQSSG
119930339       -KQSSG
119913177       -KQSSG
38503307        -KQSSG
38503308        -KQSSG
119930307       -KQSSG
62896747        -KQSSG
88703062        -KQSSG
57113861        -KQSSG
148680472       -KQNSG
124486640       -KQNSG
28204689        -KQSSG
28204681        -KQSSG
167045794       -KQSSG
119902798       -KQSSG
28204665        -KQSSG
76660997        -KQSSG
149712494       -KQSSG
119923668       -KQSSG
90820002        -KAASG
119923854       -KQSSG
70909479        -K----
109067331       ------
125979105       -K----
24663668        -K----
146285351       -KAASG
148691968       -TEQ--
170285559       -K----
112984078       -KVAA-
91083095        -K----
75029893        -KSQ--
74844658        -KVA--
66517407        -K----
70909489        -KAA--
70909487        -K----
156542863       -K----
70909485        -KS---
440853          -K----
74007700        -KQSSG
74829226        -KGA--
22138108        -QQATA
125593989       -AAAEA
17979233        -QQAA-
118573852       -K----
18415395        -QQAA-
1173256         -QNAA-
118480993       -SQAAA
118484853       -SQAAA
```

15237195	-QQAA-
18398393	-QQA--
116871421	-KAATA
73759787	-QSAT-
50550587	-QE---
45187482	-Q----
109067333	------
9759565	-QQAA-
48376549	-KQSSG
149287204	-KAQ--
82400118	-QSAT-
337930	-KQSSG
159145754	-KA---
1173257	-QSAT-
19112469	-K----
148695326	-KQSSG
50303511	------
71000467	------
19113142	-K----
19115086	-K----
168027041	LKQAA-
146413813	-Q----
164429760	-ALA--
6321997	-Q----
3914899	------
156362157	-KAK--
119493023	------
126139561	-Q----
145610060	------
154273927	-ALA--
126273970	-Q----
22758868	-KSS--
3724352	-K----
119192028	-ALA--
44894458	------
68477663	-Q----
145245281	-Q----
116193489	-TLAS-
125552065	-AAAEA
115463485	-AAAEA
158187698	-KNQ--
48596901	------
2345154	------
157690730	-KS---
168014621	-LKASA
94468404	-KAA--
108861826	-RR---
115436514	------
44967243	------
115443689	------
109509108	-KAA--
168014095	-LKAAA
121704880	------
168057168	------
50287861	-Q----
167998342	-LKAAA
61654708	-KQ---
29841450	-RR---
170942476	------
154290538	------
168066840	-EKQAA
170034084	-KAA--
170574388	-KRAA-
169595016	------
158286707	-KAA--
44967294	------
156035911	------
116779177	------
119632119	-KQSSG
170086746	-RA---
116781974	------
149239787	-RQQRG
28630198	------
50426545	-Q----
50423849	-Q----
167526949	-KAKAA
159466042	-GQA--
82907488	-KQS--
169849885	-AAA--
71015994	-KEARG
119592222	------
145286314	-KKA--
149635430	------

95007264	-QQ---
1350992	-Q----
47208976	-KQAS-
58259587	------
28630195	------
157337052	-QAAS-
66823117	------
17367482	------
17543386	-KH---
157756381	-KH---
28630193	------
79327186	-QQAA-
157357316	-QASA-
10177019	-QQAA-
145355247	------
6598334	-QQA--
58257447	------
71029346	------
146418285	-Q----
46128673	------
37779112	------
67537248	------
47777385	-AAAEA
68475117	-Q----
164656677	-QR---
115402491	------
156088951	------
57047769	------
149244128	-RQQRG
145528524	------
84997205	------
126654348	-HRN--
124803509	------
167389537	------
55982009	------
70946979	-QNN--
57107829	------
159114046	-IAKRG
156098302	-QNN--
67465453	------
167391306	------
68073433	-QNN--
2463335	------
73954159	------
145475847	------
115692154	-R----
145488765	------
118573853	------
160550143	------
146080976	-AEAR-
145499397	------
82539137	-QNN--
56199522	------
118376768	------
154333974	-AEAR-
160331849	------
169802405	------
167379757	------
109124282	------
2500489	------
157866320	-AEAR-
146080974	-AEAR-
123477532	------
4432939	------
71755051	-AETR-
123376214	------
145495133	------
145500014	------
71415246	-AEAR-
123439290	------
162606302	------
67465619	------
167393127	------
148700270	------
70909483	------
119623175	------
57641464	------
14591522	------
124028167	------
15678046	------
14520545	------
